# Association between *miR‐146a* rs2910164, *miR‐196a2* rs11614913, and *miR‐499* rs3746444 polymorphisms and the risk of esophageal carcinoma: A case–control study

**DOI:** 10.1002/cam4.4729

**Published:** 2022-05-02

**Authors:** Chao Liu, Wenhui Gao, Yijun Shi, Lu Lv, Weifeng Tang

**Affiliations:** ^1^ Department of Cardiothoracic Surgery Affiliated People's Hospital of Jiangsu University (Zhenjiang First People's Hospital) Jiangsu Province China; ^2^ School of Medicine Jiangsu University Jiangsu Province China; ^3^ Department of Cardiothoracic Surgery Nanjing Drum Tower Hospital Clinical College of Jiangsu University Jiangsu Province China

**Keywords:** ESCC, microRNA, polymorphism, susceptibility

## Abstract

MicroRNAs (miRNAs) are a group of small, non‐coding, and endogenous RNAs that regulate gene expression and over 50% of them are located at cancer‐related genomic regions or fragile sites. According to previous studies there is significant association of miRNA single nucleotide polymorphisms (SNPs) with tumorigenesis (e.g., esophageal cancer, hepatocellular cancer, gastric cancer, bladder cancer, breast cancer, lung cancer, and colon cancer), however, the conclusions have been inconsistent. To investigate the relationship between *miR‐146a* rs2910164 C > G, *miR‐196a2* rs11614913 T > C, and *miR‐499* rs3746444 A > G polymorphisms and the susceptibility to esophageal squamous cell cancer (ESCC) in the Chinese Han nationality, we recruited 829 cases and 1522 controls in our study. In this case–control study, our results suggest that the rs3746444 GG genotype increased ESCC risk [homozygote model: adjusted odds ratio (OR), 2.26; 95% CI, 1.33–3.83; *p* = 0.003, recessive model: adjusted OR, 2.34; 95% CI, 1.38–3.96; *p* = 0.002], which remained consistent after Bonferroni correction. There was no association of rs11614913 and rs2910164 polymorphisms with ESCC. After adjusting by age, sex, smoking, and drinking status and body mass index (BMI), the multiple logistic analysis suggested that rs11614913 T → C variation reduced ESCC susceptibility in females and in the ≥63 years old subgroups, while rs2910164 C → G variation increased ESCC risk in both two BMI subgroups.

## Graphical abstract

We genotyped and assayed the SNPs of rs11614913, rs2910164, and rs3746444 with propriety multiplex SNP genotyping system – SNPScan, which use the highly specific ligation reaction to discriminate alleles. Fluorescence signals are read out and genotypes are determined using the software, GeneMapper. We found that rs3746444 GG genotype increased ESCC risk.

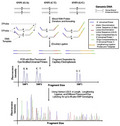

## 1 INTRODUCTION

Esophageal cancer is a major public health problem and social burden in China, and it is the fourth leading cause of cancer death with a 8.04% mortality rate in 2015.^1^ There are two main types of esophagus cancer: esophageal squamous cell cancer (ESCC) and esophageal adenocarcinoma (EAC). ESCC accounts for more than 90% of the cases in China^2^; however, since the 1970s, the incidence of EAC has significantly increased in the Western countries and has now overtaken ESCC to become the most frequent subtype of esophageal cancer. ESCC is still the main histological type with high mortality due to advanced stage at diagnosis in East Asia.^3^ Various environmental factors are involved in the incidence of ESCC in the Chinese populations, such as tobacco, alcohol, and lower BMI, but only a subset of individuals are susceptible to ESCC. This suggests that some genes might play a vital role in carcinogenesis of ESCC, and genetic factors could partly explain personal differences in ESCC occurrence.^4^

MicroRNAs (MiRNAs) comprise a novel group of ancient, small nonprotein‐coding and single‐stranded RNA negative regulatory molecules (19–25 nucleotides) that can inactivate target mRNAs and have been found in viruses, plants, and animals.^5^, ^6^, ^7^ They can target to more than 30% of protein‐coding genes,^8^, ^9^ accounting for 1% of the total protein‐coding genes.^10^, ^11^, ^12^ Generally, miRNAs resemble small‐interfering RNAs. They are considered to play critical roles in transcriptional and translational regulation via pairing with specific mRNAs,^13^, ^14^ resulting in either mRNAs degradation or protein translational repression,^15^ regulating target genes expression.^14^ One single miRNA may target tens of mRNAs. According to the overview of miRNAs, they are a part of cellular communication and a key molecular components of the cell in both normal and pathologic states.^13^ A study reported that over 50% of miRNA genes are located at tumor‐related genomic regions or fragile sites.^16^ Previous researches verified that some miRNAs were related to oncogenesis.^17^

Single nucleotide polymorphisms (SNPs) are the most common type of genetic variation, occurring approximately every 1000–2000 bases when comparing two human chromosomes, and there were 30,000–500,000 SNPs in a dense map that had been identified in a scan of the human genome for haplotypes associated with diseases.^18^, ^19^, ^20^, ^21^, ^22^, ^23^ They can change miRNA expression, maturation, and further influence the affinity between miRNAs and their target genes,^24^, ^25^ and lead to tumorigenesis.^26^ Considering the pathological and biological significance of miRNA, mutations in functional genes were activated in ESCC generation and development. Previous studies indicated that variation in rs11614913 T/C could reduce ESCC risk.^27^, ^28^, ^29^ A recent meta‐analysis identified that rs2910164 C > G SNP increased the risk of digestive system cancer (DSC).^30^ Yang^31^ found that a rs3746444 polymorphism could significantly increase the risk of DSC. To verify the potential relationship of rs11614913, rs2910164, and rs3746444 polymorphisms to ESCC susceptibility, we enrolled 2351 participants in our study.

## 2 MATERIALS AND METHODS

With Ethics Committee approval of Zhenjiang First People's Hospital (ID: K‐20210064‐W), 829 ESCC patients (638 males and 191 females) who had been treated at either the Department of Thoracic Surgery of the Zhenjiang First People's Hospital (Zhenjiang, China) or the Fujian Union Hospital (Fuzhou, China) were consecutively enrolled during the period of 2013 to 2018. All the patients with dysphagia alone or with other symptoms such as chest pain and weight loss and were diagnosed on the basis of flexible upper endoscopy with biopsy.^32^ Patients who previously had cancer, radiotherapy and chemotherapy, or metastasized cancer or cancer of unknown origins were excluded. We also recruited 1522 healthy subjects (1144 males and 378 females) as controls, all of whom were non‐cancer patients. We used a pre‐tested questionnaire to acquire the individual data of patients and related risk factors (e.g., sex, age, alcohol, and tobacco use) through trained interviewers. All participants were told the details of our investigation and signed the informed consent.

The blood samples were obtained from peripheral vessels and collected in tubes contained Ethylene Diamine Tetraacetic Acid (EDTA). We utilized the Promega kit (Promega, Madison, USA) to extract genomic DNA from lymphocytes according to the instructions of the manufacturer, and then amplified sample DNA by polymerase chain reaction (PCR). Three loci, including rs11614913, rs2910164, and rs3746444, were assayed with a custom‐by‐design 2x48‐Plex SNPscan™ Kit (Genesky Biotechnologies, Inc.,) according to the manufacturer's manual (http://www.geneskybiotech.com/en/index.php/Index/fuwuer/id/29) and previously published articles,^33^, ^34^, ^35^, ^36^ which was based on double ligation and multiplex fluorescence PCR. For quality control, we randomly selected 4% of the DNA samples and genotyped again with the same method by different laboratory staff according to our previous report,^37^ and the accuracy of each assay was confirmed.

Statistics were performed using SAS software (version 9.4). Differences between cases and controls in age were assessed with Student's *t*‐test. Differential of risk factors (such as smoking and drinking), gender distribution, other selected variables, and the genotype frequency in ESCC patients and controls was evaluated with a χ^2^ test. The crude/adjusted odds ratios (ORs) and the corresponding 95% confidential intervals (CIs) were used to estimate the association between rs2910164, rs11614913, and rs3746444 and the risk of ESCC. Haplotype frequencies of *microRNAs* were tested through the website (http://analysis.bio‐x.cn/myAnalysis.php) and data analyzed using GraphPad Prism 5 software. The deviation of the Hardy–Weinberg equilibrium (HWE) in the control group was tested with an online χ^2^ test (http://ihg.gsf.de/cgi‐bin/hw/hwa1.pl). Differences were considered to have statistical significance when *p*‐values were less than 0.05.

## 3 RESULTS

### 3.1 Characteristics of the study population

Total of 2351 individuals (829 patients with ESCC and 1522 healthy participants) were recruited in our study (Table 1). The ESCC cases were compared with the controls well‐matched for age and sex by using the chi‐square test (*p* = 0.305 and *p* = 0.331). There were significant differences in smoking, drinking status, and BMI between cases and controls (*p* < 0.001). The main data of the three polymorphic sites are summarized in Table 2. The successful genetic typing of all samples were 99.54% for these three SNPs. Genotype distribution of rs11614913, rs2910164, and rs3746444 are showed in Table 3. The rs2910164 CC, CG, GG frequencies were 38.28%, 47.72%, and 14.00% in the control group compared to 36.52%, 49.19%, and 14.29% in ESCC patients. The frequencies of rs11614913 TT, TC, CC were 28.65%, 52.08%, and 19.27% in control subjects compare to 31.30%, 49.44%, and 19.25% in ESCC patients; the frequencies of *miRNA‐499* rs3746444 AA, AG, GG were 71.75%, 26.07%, and 2.18% in controls compare to 72.46%, 23.33%, and 4.22% in patients with ESCC. The *p*‐value of HWE in controls were 0.578, 0.048, and 0.680, respectively (Table 2).

**TABLE 1:** Distribution of selected demographic variables and risk factors in cases and controls

Variable	Overall Cases (*n* = 829)		Overall Controls (*n* = 1522)		*p* value^a^
	*n*	%	*n*	%	
Age, mean (±SD)	62.55 ± 8.19		62.94 ± 9.06		0.305
Age (years)					
<63	391	47.17	750	49.28	0.328
≥63	438	52.83	772	50.72	
Sex					
Male	638	76.96	1144	75.16	0.331
Female	191	23.04	378	24.84	
Smoking status					**<0.001**
Never	385	46.44	1100	72.27	
Ever	444	53.56	422	27.73	
Alcohol use					**<0.001**
Never	570	68.76	1346	88.44	
Ever	259	31.24	176	11.56	
BMI					**<0.001**
<24	608	73.34	800	52.56	
≥24	221	26.66	722	47.44	
Lymph node status					
Positive	427	51.51			
Negative	40,	48.49			

*Note*: Bold values are statistically significant (*p* < 0.05).

^a^ Two‐sided chi‐square and Student's *t*‐test.

**TABLE 2:** Primary information for *microRNA* polymorphisms

Genotyped SNPs	Chr	Chr Pos (NCBI Build 37)	Region	MAF^a^ for Chinese in database	MAF in our controls (*n* = 1522)	*P* value for HWE^b^ test in our controls	Genotyping method	Genotyping value (%)
*hsa‐mir‐146a* rs2910164 C > G	5	159,912,418	3'UTR	0.43	0.38	0.578	SNPscan	99.54
*hsa‐mir‐196a2* rs11614913 T > C	12	54,385,599	3'UTR	0.45	0.45	0.048	SNPscan	99.54
*hsa‐mir‐499* rs3746444 A > G	20	33,578,251	Intron	0.15	0.15	0.680	SNPscan	99.54

Abbreviations: Chr, chromosome; mir, miRNA; SNPs, single nucleotide polymorphisms.

^a^ MAF, minor allele frequency.

^b^ HWE, Hardy–Weinberg equilibrium.

**TABLE 3:** Logistic regression analyses of associations between *miRNA‐146a* rs2910164 C > G*, miRNA‐196a2* rs11614913 T > C, and *miRNA‐499* rs3746444 A > G polymorphisms and risk of ESCC

Genotype	Cases (*n* = 829)		Controls (*n* = 1522)		Crude OR (95% CI)	*p*	Adjusted OR^a^ (95% CI)	*p*
*hsa‐mir‐146a* rs2910164 C > G								
CC	294	36.52	580	38.28	1.00			
CG	396	49.19	723	47.72	1.08 (0.90–1.30)	0.415	1.03 (0.85–1.26)	0.750
GG	115	14.29	212	14.00	1.07 (0.82–1.40)	0.619	1.16 (0.87–1.54)	0.307
CG + GG	511	63.48	935	61.72	1.08 (0.90–1.29)	0.405	1.06 (0.88–1.28)	0.545
CC + CG	690	85.71	1303	86.01	1.00			
GG	115	14.29	212	13.99	1.02 (0.80–1.31)	0.847	1.14 (0.88–1.48)	0.330
G allele	626	38.88	1147	37.68				
*hsa‐mir‐196a2* rs11614913 T > C								
TT	252	31.30	434	28.65	1.00			
TC	398	49.44	789	52.08	0.87 (0.71–1.06)	0.161	0.84 (0.68–1.04)	0.111
CC	155	19.25	292	19.27	0.91 (0.71–1.17)	0.480	0.89 (0.68–1.16)	0.374
TC + CC	553	68.70	1081	71.03	0.88 (0.73–1.06)	0.182	0.85 (0.70–1.04)	0.122
TT + TC	650	80.75	1223	80.35	1.00			
CC	155	19.25	292	19.27	1.00 (0.80–1.24)	0.991	0.99 (0.78–1.25)	0.912
C allele	708	42.70	1373	45.11				
*hsa‐mir‐499* rs3746444 A > G								
AA	584	72.46	1087	71.75	1.00			
AG	188	23.33	395	26.07	0.87 (0.73–1.08)	0.237	0.87 (0.70–1.08)	0.206
GG	34	4.22	33	2.18	**1.92 (1.18–3.13)**	**0.009**	**2.26 (1.33–3.83)**	**0.003**
AG + GG	222	27.54	428	28.25	0.97 (0.80–1.17)	0.719	0.97 (0.79–1.19)	0.739
AA+AG	772	95.78	1482	97.82	1.00			
GG	34	4.22	33	2.18	**1.98 (1.22–3.22)**	**0.006**	**2.34 (1.38–3.96)**	**0.002**
G allele	256	15.44	461	15.14				

Abbreviation: OR, odds ratio.

^a^ Adjusted for age, sex, smoking, alcohol use, and BMI in a logistic model. Bold values are statistically significant (*p* < 0.05).

### 3.2 Relationship between the miRNA loci and ESCC susceptibility

The genetic type distribution and allele frequencies of rs2910164 and rs11614913 polymorphisms in ESCC cases were not significantly different from the controls, while the rs3746444 GG genotype related to ESCC risk (crude OR = 1.92; 95% CI,1.18–3.13; *p* = 0.009) when compared to the rs3746444 AA genotype, and the frequency of rs3746444 GG also had a close association with an increased risk of ESCC when the rs3746444 AA/AG genotype as reference (crude OR = 1.98; 95% CI,1.22–3.22; *p* = 0.006). After adjustment for gender, age, tobacco using, BMI, and alcohol consumption, there was a significant connection between the rs3746444 GG genotype and increased risk of ESCC as well (GG vs. AA: *p* = 0.003; GG vs. AA/AG: *p* = 0.002, Table 3).

### 3.3 Relationship between the miRNA loci and ESCC risk in different subgroups

We analyzed the genotype frequencies of rs11614913, rs2910164, and rs3746444 SNPs in different age, sex, drinking, smoking, and BMI groups (Tables 4, 5, 6). After adjusting the sex, age, alcohol consumption, smoking status, and BMI, multivariate regression analysis showed that the rs2910164 C → G variation increased ESCC risk in the BMI group (BMI ≥ 24 kg/m^2^ subgroup: CG vs. CC: *p* = 0.047; BMI < 24 kg/m^2^ subgroup: GG vs. CC/CG: *p* = 0.019 [Table 4]). The rs11614913 T → C variation decreased ESCC risk both in sex and age groups (female subgroup: TC vs. TT: *p* = 0.018; CC vs. TT: *p* = 0.039; TC/CC vs. TT: *p* = 0.011; age ≥ 63 years subgroup: TC vs. TT: *p* = 0.048 [Table 5]). For rs3746444, the genotype could influence ESCC risk in all groups (male subgroup: GG vs. AA: *p* = 0.045 and GG vs. AA/AG: *p* = 0.032; female subgroup: GG vs. AA: *p* = 0.018 and GG vs. AA/AG: *p* = 0.016; <63 years subgroup: GG vs AA: *p* = 0.0004 and GG vs. AA/AG: *p* = 0.0002; non‐smoking subgroup: GG vs. AA: *p* = 0.018 and GG vs. AA/AG: *p* = 0.015; non‐drinking subgroup: GG vs. AA: *p* = 0.001 and GG vs. AA/AG: *p* = 0.001; BMI ≥ 24 kg/m^2^ subgroup: GG vs. AA: *p* = 0.001 and GG vs. AA/AG: *p* = 0.001 [Table 6]), but decreased the risk of ESCC in the drinking subgroup (AG vs. AA: *p* = 0.018 and AG/GG vs. AA: *p* = 0.026 [Table 6]).

**TABLE 4:** Stratified analyses between *miRNA‐146a* rs2910164 C > G polymorphism and ESCC risk by sex, age, BMI, smoking status, and alcohol consumption

Variable	*miRNA‐146a*rs2910164 C > G (case/control)^a^			Adjusted OR^b^ (95% CI); *p*			
	CC	CG	GG	Additive model	Homozygote model	Dominant model	Recessive model
Sex							
Male	227/440	304/541	85/156	1.06 (0.84–1.34) *p*: 0.631	1.18 (0.84–1.65) *p*: 0.340	1.08 (0.87–1.35) *p*: 0.475	1.14 (0.84–1.56) *p*: 0.405
Female	67/140	92/182	30/56	1.03 (0.69–1.53) *p*: 0.893	1.16 (0.67–2.01) *p*: 0.605	1.06 (0.72–1.54) *p*: 0.773	1.14 (0.69–1.89) *p*: 0.612
Age							
<63	147/295	179/344	54/105	1.03 (0.77–1.39) *p*: 0.838	1.18 (0.77–1.81) *p*: 0.454	1.06 (0.80–1.41) *p*: 0.670	1.16 (0.78–1.73) *p*: 0.468
≥63	147/285	217/379	61/107	1.06 (0.80–1.39) *p*: 0.689	1.16 (0.78–1.71) *p*: 0.464	1.08 (0.83–1.40) *p*: 0.569	1.12 (0.79–1.60) *p*: 0.533
Smoking status							
Never	134/421	180/513	64/162	1.06 (0.81–1.38) *p*: 0.672	1.24 (0.87–1.78) *p*: 0.233	1.10 (0.86–1.42) *p*: 0.444	1.21 (0.87–1.67) *p*: 0.263
Ever	160/159	216/210	51/50	1.03 (0.75–1.40) *p*: 0.871	1.08 (0.67–1.74) *p*: 0.763	1.04 (0.77–1.39) *p*: 0.819	1.06 (0.68–1.65) *p*: 0.800
Alcohol consumption							
Never	198/522	270/631	84/188	1.10 (0.88–1.37) *p*: 0.418	1.24 (0.91–1.70) *p*: 0.178	1.13 (0.91–1.40) *p*: 0.263	1.18 (0.88–1.58) *p*: 0.263
Ever	96/58	126/92	31/24	0.82 (0.52–1.30) *p*: 0.396	0.83 (0.42–1.66)	0.82 (0.53–1.28) *p*: 0.383	0.94 (0.50–1.76)
					*p*: 0.596		*p*: 0.834
BMI (kg/m^2^)							
<24	223/300	277/397	89/99	0.88 (0.69–1.12) *p*: 0.299	1.38 (0.96–1.97) *p*: 0.078	0.97 (0.77–1.22) *p*: 0.792	**1.48 (1.07–2.06)** ***p*: 0.019**
≥24	71/280	119/326	26/113	**1.42 (1.01–2.00)** ***p*: 0.047**	0.86 (0.51–1.44) *p*: 0.560	1.27 (0.91–1.77) *p*: 0.154	0.70 (0.44–1.21) *p*: 0.138

*Note*: Bold values are statistically significant (*p* < 0.05). ESCC, esophageal squamous cell cancer; OR, odds ratio.

^a^ The genotyping was successful in 805 (97.10%) ESCC cases, and 1515 (99.54%) controls for *miRNA‐146a* rs2910164 C > G.

^b^ Adjusted for age, sex, BMI, smoking status, and alcohol consumption (besides stratified factors accordingly) in a logistic regression model.

**TABLE 5:** Stratified analyses between *miRNA‐196a2* rs11614913 T > C polymorphism and ESCC risk by sex, age, BMI, smoking status, and alcohol consumption

Variable	*miRNA‐196a2* rs11614913 T > C (case/control)^a^			Adjusted OR^b^ (95% CI); *p*			
	TT	TC	CC	additive model	homozygote model	Dominant model	Recessive model
Sex							
Male	193/346	303/578	120/213	0.92 (0.72–1.17) *p*: 0.482	1.00 (0.73–1.36) *p*: 0.993	0.94 (0.75–1.18) *p*: 0.588	1.05 (0.80–1.38) *p*: 0.704
Female	59/88	95/211	35/79	**0.59 (0.39–0.92)** ***p*: 0.018**	**0.56 (0.33–0.97)** ***p*: 0.039**	**0.59 (0.39–0.88)** ***p*: 0.011**	0.80 (0.51–1.27) *p*: 0.345
Age							
<63	121/228	193/373	66/143	0.95 (0.69–1.29) *p*: 0.730	0.90 (0.60–1.35) *p*: 0.625	0.94 (0.70–1.26) *p*: 0.656	0.94 (0.66–1.34) *p*: 0.712
≥63	131/206	205/416	89/149	**0.75 (0.56–1.00)** ***p*: 0.048**	0.86 (0.60–1.23) *p*: 0.402	0.78 (0.59–1.02) *p*: 0.070	1.04 (0.76–1.41) *p*: 0.826
Smoking status							
Never	116/314	192/563	70/219	0.88 (0.66–1.16) *p*: 0.347	0.83 (0.58–1.18) *p*: 0.289	0.86 (0.66–1.12) *p*: 0.267	0.90 (0.66–1.22) *p*: 0.496
Ever	136/120	206/226	85/73	0.77 (0.55–1.06) *p*: 0.112	0.99 (0.65–1.52) *p*: 0.978	0.82 (0.60–1.12) *p*: 0.217	1.18 (0.81–1.70) *p*: 0.388
Alcohol consumption							
Never	178/379	268/702	106/260	0.79 (0.63–1.00) *p*: 0.052	0.84 (0.62–1.13) *p*: 0.237	0.80 (0.64–1.00) *p*: 0.054	0.97 (0.75–1.26) *p*: 0.802
Ever	74/55	130/87	49/32	1.02 (0.63–1.66) *p*: 0.941	1.12 (0.60–2.07) *p*: 0.721	1.05 (0.66–1.66) *p*: 0.850	1.11 (0.65–1.89) *p*: 0.712
BMI (kg/m^2^)							
<24	184/233	291/405	114/158	0.88 (0.68–1.15) *p*: 0.345	0.87 (0.63–1.21) *p*: 0.421	0.88 (0.69–1.13) *p*: 0.308	0.95 (0.71–1.26) *p*: 0.700
≥24	68/201	107/384	41/134	0.77 (0.54–1.11) *p*: 0.158	0.91 (0.57–1.44) *p*: 0.679	0.81 (0.57–1.13) *p*: 0.214	1.07 (0.72–1.60) *p*: 0.740

*Note*: Bold values are statistically significant (*p* < 0.05). ESCC, esophageal squamous cell cancer; OR, odds ratio.

^a^ The genotyping was successful in 805 (97.10%) ESCC cases, and 1515 (99.54%) controls for *miRNA‐196a2* rs11614913.

^b^ Adjusted for age, sex, BMI, smoking status, and alcohol consumption (besides stratified factors accordingly) in a logistic regression model.

**TABLE 6:** Stratified analyses between *miRNA‐499* rs3746444 A > G polymorphism and ESCC risk by sex, age, smoking status, and alcohol consumption

Variable	*miRNA‐499* rs3746444 A > G (case/control)^a^			Adjusted OR^b^ (95% CI); *p*			
	AA	AG	GG	Additive model	Homozygote model	Dominant model	Recessive model
Sex							
Male	451/814	141/294	25/29	0.85 (0.66–1.10) *p*: 0.215	**1.85 (1.01–3.36)** ***p*: 0.045**	0.93 (0.74–1.18) *p*: 0.568	**1.92 (1.06–3.48)** ***p*: 0.032**
Female	133/273	47/101	9/4	0.93 (0.61–1.41) *p*: 0.729	**4.64 (1.30–16.55)** ***p*: 0.018**	1.06 (0.71–1.58) *p*: 0.782	**4.73 (1.33–16.80)** ***p*: 0.016**
Age							
<63	284/536	74/192	22/16	0.76 (0.54–1.06) *p*: 0.100	**3.72 (1.80–7.70)** ***p*: 0.0004**	0.94 (0.69–1.28) *p*: 0.686	**3.98 (1.93–8.21)** ***P*: 0.0002**
≥63	300/551	114/203	12/17	0.99 (0.75–1.31) *p*: 0.938	1.27 (0.57–2.83) *p*: 0.553	1.01 (0.77–1.33) *p*: 0.941	1.28 (0.58–2.82) *p*: 0.546
Smoking status							
Never	272/794	89/276	17/26	0.93 (0.70–1.23) *p*: 0.590	**2.20 (1.15–4.22)** ***p*: 0.018**	1.02 (0.78–1.34) *p*: 0.864	**2.24 (1.17–4.28)** ***p*: 0.015**
Ever	312/293	99/119	17/7	0.83 (0.60–1.15) *p*: 0.257	2.39 (0.93–6.11) *p*: 0.069	0.91 (0.67–1.25) *p*: 0.572	2.52 (1.00–6.41) *p*: 0.053
Alcohol consumption							
Never	386/970	140/341	27/30	0.99 (0.78–1.26) *p*: 0.932	**2.55 (1.45–4.46)** ***p*: 0.001**	1.10 (0.88–1.38) *p*: 0.395	**2.55 (1.46–4.46)** ***p*: 0.001**
Ever	198/117	48/54	7/3	**0.56 (0.34–0.90)** ***p*: 0.018**	1.12 (0.24–5.18) *p*: 0.883	**0.59 (0.37–0.94)** ***p*: 0.026**	1.31 (0.28–6.03) *p*: 0.733
BMI (kg/m^2^)							
<24	428/575	139/200	22/21	0.92 (0.70–1.19) *p*: 0.514	1.61 (0.84–3.08) *p*: 0.154	0.98 (0.76–1.26) *p*: 0.847	1.64 (0.86–3.14) *p*: 0.134
≥24	156/512	49/195	12/12	0.80 (0.55–1.15) *p*: 0.229	**4.17 (1.76–9.87)** ***p*: 0.001**	0.96 (0.68–1.35) *p*: 0.798	**4.42 (1.88–10.41)** ***p*: 0.0007**

*Note*: Bold values are statistically significant (*p* < 0.05).

Abbreviations: ESCC, esophageal squamous cell cancer; OR, odds ratio.

^a^ The genotyping was successful in 806 (97.23%) ESCC cases, and 1515 (99.54%) controls for *miRNA‐499* rs3746444 A > G.

^b^ Adjusted for age, sex, BMI, smoking status, and alcohol consumption (besides stratified factors accordingly) in a logistic regression model.

### 3.4 Relationship between the miRNA SNP haplotypes and ESCC risk

We constructed eight haplotypes by using an expectation–maximization algorithm on website (http://analysis.bio‐x.cn/myAnalysis.php) (Table 7) according to Yongyong Shi.^38^ When T_rs11614913_C_rs2910164_A_rs3746444_ was used as a reference (because it was the haplotype with highest frequency), a relationship of seven other haplotypes frequencies to the risk of ESCC was not observed (all *p* values >0.05).

**TABLE 7:** *miRNA* haplotype frequencies (%) in cases and controls and risk of ESCC

Haplotypes	case (*n* = 829)		control (*n* = 1522)		Crude OR (95% CI)	*p*
	*n*	%	*n*	%		
T_rs11614913_C_rs2910164_A_rs3746444_	486	29.31	862	28.32	Reference	
C_rs11614913_C_rs2910164_A_rs3746444_	355	21.41	743	24.41	0.85 (0.80–1.00)	0.054
T_rs11614913_G_rs2910164_A_rs3746444_	296	17.85	544	17.87	0.97 (0.87–1.10)	0.699
C_rs11614913_G_rs2910164_A_rs3746444_	217	13.09	420	13.80	0.92 (0.83–1.08)	0.387
C_rs11614913_C_rs2910164_G_rs3746444_	77	4.64	128	4.20	1.07 (0.86–1.26)	0.676
T_rs11614913_C_rs2910164_G_rs3746444_	66	3.98	150	4.93	0.78 (0.68–1.05)	0.117
C_rs11614913_G_rs2910164_G_rs3746444_	59	3.56	82	5.39	1.28 (0.94–1.43)	0.174
T_rs11614913_G_rs2910164_G_rs3746444_	54	3.26	101	3.32	0.95 (0.77–1.21)	0.765

### 3.5 Relationship between the miRNA loci and lymph node metastases in ESCC cases

Association between miRNAs loci and lymph node metastases in ESCC cases fell short of statistical significance in our research (Table 8).

**TABLE 8:** Logistic regression analyses of association between *miRNA‐146a* rs2910164 C > G*, miRNA‐196a2* rs11614913 T > C, and *miRNA‐499* rs3746444 A > G polymorphisms and lymph node status in ESCC patients

Genotype	Positive (*n* = 427)		Negative (*n* = 402)		Crude OR (95% CI)	*p*	Adjusted OR^a^ (95% CI)	*p*
	*n*	%	*n*	%				
*miRNA‐146a* rs2910164 C > G								
CC	148	34.66	146	36.32	1.00		1.00	
CG	215	50.35	181	45.02	1.17 (0.87–1.59)	0.304	1.23 (0.90–1.67)	0.193
GG	52	12.18	63	15.67	0.81 (0.53–1.26)	0.352	0.83 (0.53–1.29)	0.399
CG + GG	267	62.53	244	60.70	1.08 (0.81–1.44)	0.602	1.12 (0.84–1.50)	0.441
CC + CG	363	85.01	327	81.34	1.00		1.00	
GG	52	12.18	78	19.40	0.74 (0.50–1.11)	0.144	0.74 (0.49–1.10)	0.138
G allele	319	37.35	307	38.18				
*miRNA‐196a2* rs11614913 T > C								
TT	130	30.44	122	30.35	1.00		1.00	
TC	208	48.72	190	47.26	1.03 (0.75–1.41)	0.867	1.03 (0.75–1.42)	0.847
CC	77	18.03	78	19.40	0.93 (0.62–1.38)	0.708	0.93 (0.62–1.39)	0.717
TC + CC	285	66.74	268	66.67	1.00 (0.74–1.35)	0.989	1.00 (0.74–1.36)	0.992
TT + TC	338	79.16	312	77.61	1.00		1.00	
CC	77	18.03	78	19.40	0.91 (0.64–1.29)	0.603	0.91 (0.64–1.30)	0.604
Callele	362	42.39	346	43.03				
*miRNA‐499* rs3746444 A > G								
AA	299	70.02	285	70.90	1.00		1.00	
AG	95	22.25	93	23.13	0.97 (0.70–1.35)	0.874	1.00 (0.72–1.40)	0.999
GG	21	4.92	13	3.23	1.54 (0.76–3.13)	0.234	1.62 (0.78–3.35)	0.196
AG + GG	116	27.17	106	26.37	1.04 (0.77–1.42)	0.789	1.07 (0.78–1.47)	0.656
AA+AG	394	92.27	378	94.03	1.00		1.00	
GG	21	4.92	13	3.23	1.55 (0.77–3.14)	0.224	1.62 (0.78–3.33)	0.194
G allele	137	16.04	119	14.80				

Abbreviations: ESCC, Esophageal squamous cell carcinoma; OR, odds ratio.

^a^ Adjusted for age, sex, alcohol use, smoking status, and BMI.

## 4 DISCUSSION

Previous studies confirmed that *miR‐146a* rs2910164, *miR‐196a2* rs11614913, and *miR‐499* rs3746444 polymorphisms could increase the risk of various cancers, such as esophagogastric junction adenocarcinoma, breast cancer, lung cancer, cervical cancer, and non‐Hodgkin lymphoma.^35^, ^39^, ^40^, ^41^, ^42^, ^43^ However, the conclusions of published reports have been inconsistent regarding esophageal cancer. We analyzed 2351 specimens to evaluate the relationship of *miR‐146a* rs2910164, *miR‐196a2* rs11614913, and *miR‐499* rs3746444 polymorphisms to ESCC susceptibility. Our results showed that the rs3746444 G allele significantly increased ESCC risk, while rs2910164 and rs11614913 SNPs were not related to ESCC in overall result. Moreover, for subgroup analyses, multivariate regression analysis revealed that the rs3746444 GG genotype increased ESCC risk in male, female, age < 63 years, never smoked, never drink, and BMI ≥24 kg/m^2^ subgroups but decreased ESCC risk in the subgroup of alcohol consumption. The rs2910164 C > G polymorphism increased ESCC risk in the BMI <24 kg/m^2^, ≥24 kg/m^2^ subgroups, and the rs11614913 T > C SNP decreased ESCC risk in the female and age ≥ 63 years subgroups. However, we should interpret the results with more cautions, and follow‐up experiments with more specimens and gene–environment factors should be performed.

*MiRNA‐499* is located on chromosome 20, and rs3746444 is in the seed sequence of *miR‐499a‐3p*, which may reduce the expression of *miRNA‐499a*.^44^ The *SRY‐sex‐determining region box (SOX)* is a target gene of *miRNA‐499* that suppresses cell growth and is regarded as an antitumor factor.^45^ Li^46^ reported that *SOX‐6* overexpression could inhibit the proliferation induced by the rs3746444 A/G polymorphism, causing the activation of the Wnt/β pathway, which is involved in oncogenesis and progression. A polymorphism of rs3746444 in the *miR‐499* gene has been implicated in tumorigenesis through regulating the expression level of the SOX gene. Numerous studies have reported that *SOX‐6* expression was deregulated in ESCC, oral squamous cell cancer, and pancreatic cancer.^45^, ^47^, ^48^ A recent meta‐analysis demonstrated that a SNP of rs3746444 significantly increased DSC risk,^31^ which was consistent with our result that the rs3746444 A to G variation could increase the risk of ESCC. Shen^49^ performed a population‐based study including 1400 ESCC patients and 2185 controls and found that the rs3746444 polymorphism increased the risk of ESCC both in nonsmoking and nondrinking subgroups. However, both Umar^50^ and Wei^28^ reported that there was no correlation between rs3746444 SNP and ESCC risk.

The rs2910264 polymorphism with G to C variation occurs at the first nucleotide of the *miRNA‐146a* precursor, decreasing mature *miRNA‐146a* and *pre‐miRNA‐146a* production and increasing reported gene expression; the downregulated expression of both *miRNA‐146a* precursor and mature *miRNA‐146a* could be reversed when C was mutated back to G.^51^, ^52^ A recent meta‐analysis observed the relationship between the rs2910264 C/G locus and cancers, considering that the rs2910164 G allele was a risk of DSC.^30^ Hao^53^ analyzed the data collected from 38 independent studies and concluded that the rs2910164 polymorphism increased the risk of lung cancer and nasopharyngeal cancer, while Wang^41^ also reported that rs2910164 polymorphism was a risk factor of lung cancer. Additionally, association of rs2910164 C → G variation to ovarian cancer has been observed.^54^ However, there was no relationship of rs2910164 polymorphism and ESCC overall,^29^ which is consistent with our study, but we also verified that the rs2910164 C to G mutation could increase the risk of ESCC in both the BMI <24 and ≥ 24 subgroups.

*MiRNA‐196* is located on the *homeobox (HOX)* genes.^55^ Three genes of *miRNA‐196a* have been found so far, named *miRNA‐196a‐1*, *miRNA‐196a‐2*, and *miRNA‐196b*, respectively. *MiRNA‐196a2* is located in the region between *HOXC‐10* and *HOXC‐9* on chromosome 12,^56^ composing two mature miRNAs (*miRNA‐196a* and miRNA‐196a*), and rs11614913 is located on miRNA‐196a* mature sequence, not only influencing the mature *miRNA‐196a2* level, but also affecting target gene expression.^57^ According to the study of Hoffman,^57^ the level of mature *miRNA‐196a2* transfected with *pre‐miRNA‐196a2‐C* was higher than that transfected with *pre‐miRNA‐196a2‐T*. Previous studies reported that the homozygote genotype of rs11614913 could reduce the risk of ESCC.^27^, ^28^, ^29^ A meta‐analysis confirmed that the rs11614913 SNP had a negative correlation with cancer in overall result.^58^ Our study showed that the rs11614913 T to C variation could reduce the risk of ESCC in the female subgroup.

Haplotype analysis has stronger statistic power than single SNP analysis,^59^, ^60^ however, there were few studies about the association between SNP haplotypes and ESCC, and there were no studies about haplotypes of these three SNPs on esophageal cancer risk. We constructed a total of eight haplotype combinations and did not observe positive results. Panagiotis Dikeakos^61^ performed a haplotype analysis of *miR‐146a, miR‐149, and miR‐196a2*, and found that C_rs2910164_C_rs2292832_C_rs11614913_ and G_rs2910164_T_rs2292832_C_rs11614913_ increased the risk of GC, while C_rs2910164_T_rs2292832_T_rs11614913_ and C_rs2910164_C_rs2292832_T_rs11614913_ reduced the GC risk. Mohan Damodaran^62^ analyzed eight SNPs in microRNA and verified that a polymorphism of rs11614913 could increase prostate cancer risk. The SNP of rs2910164 did not link with prostate cancer, whereas the haplotype analysis showed that A_rs73318382_G_rs57095329_G_rs2910164_C_rs11614913_G_rs41275794_T_rs12976445_G_rs10404453_G_rs1297533_ reduced the risk of prostate cancer, indicating locus–locus interaction of SNPs and gene–gene interaction on cancer risk.

There were some limitations in our study. Primarily, other variants in miRNA genes were not estimated. Second, due to the insufficient sample size, we did not perform a replication of the study. Finally, recruiting controls from hospitals instead of from communities may not accurately represent the whole Chinese populations. Some potential influencing factors were not analyzed and this might reduce the reliability of results.

In summary, our study identified the association between miRNA loci and ESCC in a Chinese Han population. Well‐designed case–control studies including a larger sample size are required to verify these main results and validate the potential gene–gene interaction and gene–environment factors which is implicated in polymorphisms of the rs2910164 in the *miR‐146a* gene, rs11614913 in the *miR‐196a* gene, and rs3746444 in the *miR‐499* gene and susceptibility to ESCC.

## CONFLICT OF INTEREST

The authors declared that they have no conflict of interest to this work. We declare that we do not have any commercial or associative interest that represents a conflict of interest in connection with the work submitted.

## AUTHOR CONTRIBUTION

Weifeng Tang: Conception and design of experiment, revising the manuscript. Chao Liu: Analysis of data, drafting the manuscript. Wenhui Gao: Collection of specimens, extraction of DNA. Lu Lv: Acquisition of data, extraction of DNA. Yijun Shi: Genotyping.

## ETHICS STATEMENT

The study was approved by the Ethics Committee of Zhenjiang First People's Hospital (ID: K‐20210064‐W) Zhenjiang, China. All patients provided written informed consent.

## Supporting information


Appendix S1

## Data Availability

Full data are available via an online supplementary material. Supplementary files summarizes the detailed data of genotypes and the PCR program.
