# Supplementary material for: Association between miR‐146a rs2910164, miR‐196a2 rs11614913, and miR‐499 rs3746444 polymorphisms and the risk of esophageal carcinoma: A case–control study
Source: Cancer Med. 2022 May 2;11(21):3949–59. doi: 10.1002/cam4.4729 (PMC9636501; doi:10.1002/cam4.4729)
Supplement: Supplementary file 1 — Appendix S1 [file CAM4-11-3949-s001.pdf]

Table S1 raw data record

Subjects	sex (male:1, female:2)	age (year)	smoking (yes:1; no:2)	drinking (yes:1, no:0)	BMI (≥24:1, <24:0)	rs11614913 genotypes	rs2910164 genotypes	rs3746444 genotypes
case-001	2	78	0	0	0	C/C	G/C	A/A
case-002	1	78	1	1	0	C/T	G/C	A/A
case-003	1	78	1	1	0	T/T	C/C	A/A
case-004	1	78	1	0	0	T/T	G/C	A/A
case-005	1	78	0	0	0	T/T	G/C	G/A
case-006	1	78	0	0	0	C/T	C/C	A/A
case-007	2	78	0	0	1	C/C	C/C	G/A
case-008	1	78	0	0	0	T/T	C/C	A/A
case-009	2	78	0	0	0	T/T	G/C	G/A
case-010	1	78	0	0	0	C/T	C/C	A/A
case-011	1	78	1	0	0	C/T	G/C	G/A
case-012	1	78	0	0	0	C/T	C/C	G/A
case-013	1	78	1	1	0	C/C	C/C	G/A
case-014	1	78	1	1	0	T/T	C/C	A/A
case-015	1	78	0	0	1	T/T	G/C	A/A
case-016	2	78	0	0	0	T/T	G/G	A/A
case-017	1	78	0	0	1	C/T	G/C	A/A
case-018	1	78	1	1	0	T/T	C/C	A/A
case-019	1	78	0	0	0	C/T	G/C	G/G
case-020	2	78	0	0	0	T/T	C/C	G/A
case-021	1	78	1	0	0	C/T	G/C	G/A
case-022	1	78	1	0	1	C/T	G/C	A/A
case-023	1	78	1	1	0	C/C	C/C	A/A
case-024	2	78	0	0	0	T/T	G/G	G/A
case-025	1	78	1	1	0	C/T	C/C	A/A
case-026	1	78	0	0	1	C/T	C/C	A/A
case-027	1	78	0	0	0	C/C	C/C	G/A
case-028	1	78	1	1	1	C/C	G/G	G/A
case-029	2	78	0	0	1	C/T	C/C	A/A
case-030	2	78	0	0	0	C/T	G/C	A/A
case-031	1	78	1	1	0	C/T	C/C	A/A
case-032	1	78	0	1	0	C/C	G/C	A/A
case-033	2	78	0	0	0	T/T	G/C	A/A
case-034	1	78	1	1	0	T/T	C/C	A/A
case-035	1	78	1	1	0	C/C	C/C	A/A

case-036	1	78	1	0	1	C/T	C/C	G/A
case-037	1	78	1	0	0	C/T	G/C	G/A
case-038	1	78	1	1	0	C/T	G/C	A/A
case-039	1	78	0	0	0	C/T	C/C	A/A
case-040	1	78	1	1	0	C/T	C/C	A/A
case-041	1	78	0	0	1	C/T	G/C	A/A
case-042	1	78	1	0	1	C/T	G/C	G/A
case-043	2	78	0	0	1	C/T	G/C	A/A
case-044	2	78	0	0	1	T/T	C/C	A/A
case-045	1	78	1	0	0	T/T	G/C	A/A
case-046	1	78	1	0	0	T/T	G/G	A/A
case-047	2	78	0	0	0	C/T	G/C	A/A
case-048	2	78	0	0	0	T/T	C/C	A/A
case-049	1	78	0	0	1	C/T	G/C	A/A
case-050	1	78	1	1	0	C/T	C/C	G/A
case-051	1	78	1	0	1	C/C	C/C	A/A
case-052	1	78	1	1	0	C/T	G/C	A/A
case-053	1	78	1	1	0	T/T	G/C	A/A
case-054	1	78	0	0	0	T/T	C/C	A/A
case-055	1	78	0	0	0	C/T	G/G	G/A
case-056	1	78	1	1	0	C/C	C/C	G/A
case-057	1	78	1	0	0	C/C	G/G	A/A
case-058	1	78	0	0	1	C/T	G/C	G/A
case-059	1	78	1	0	0	C/T	C/C	A/A
case-060	1	78	1	1	0	C/T	C/C	G/A
case-061	1	78	1	1	1	C/T	G/C	A/A
case-062	1	78	0	0	0	C/T	C/C	A/A
case-063	2	78	0	0	0	C/T	G/C	A/A
case-064	1	78	1	1	0	T/T	C/C	G/A
case-065	1	78	1	1	0	C/C	G/C	G/A
case-066	1	78	1	1	0	T/T	G/C	A/A
case-067	1	78	0	0	1	C/T	G/C	G/A
case-068	1	78	1	0	0	T/T	G/C	A/A
case-069	1	78	0	0	0	C/T	C/C	G/G
case-070	2	78	0	0	0	T/T	G/C	A/A
case-071	2	78	0	0	0	C/T	G/G	G/A
case-072	2	78	0	0	1	C/C	G/C	A/A

case-073	1	78	1	0	0	C/T	G/C	A/A
case-074	1	78	1	0	0	T/T	C/C	A/A
case-075	2	78	0	0	0	T/T	G/C	A/A
case-076	1	78	1	1	0	T/T	G/G	A/A
case-077	1	78	1	1	0	T/T	G/C	A/A
case-078	1	78	1	0	1	C/T	G/C	G/G
case-079	1	78	1	1	0	C/T	C/C	A/A
case-080	1	78	1	0	1	T/T	G/C	G/A
case-081	1	78	1	1	0	T/T	C/C	A/A
case-082	1	78	1	1	0	T/T	C/C	A/A
case-083	1	78	1	1	0	C/T	G/C	A/A
case-084	2	78	0	0	0	C/T	G/G	A/A
case-085	2	78	0	0	1	C/C	C/C	A/A
case-086	1	78	1	1	0	C/T	G/G	A/A
case-087	1	78	1	0	1	C/T	G/C	A/A
case-088	1	78	1	0	0	C/C	C/C	A/A
case-089	1	78	1	1	0	T/T	G/C	A/A
case-090	1	78	0	0	0	T/T	G/G	G/G
case-091	1	78	1	1	0	T/T	G/C	A/A
case-092	1	78	1	0	1	T/T	G/C	A/A
case-093	1	78	1	0	0	C/C	C/C	A/A
case-094	2	78	0	0	0	C/C	C/C	A/A
case-095	1	78	1	0	0	T/T	G/C	A/A
case-096	1	78	1	0	0	C/T	G/C	G/G
case-097	1	78	1	0	0	C/T	C/C	A/A
case-098	1	78	1	0	1	C/T	G/C	A/A
case-099	1	78	1	0	1	C/C	G/C	G/A
case-100	1	78	0	0	1	T/T	G/C	G/A
case-101	2	78	0	0	0	C/T	C/C	G/G
case-102	1	78	0	0	0	T/T	C/C	A/A
case-103	2	78	0	0	0	C/T	G/C	A/A
case-104	1	78	1	1	0	C/T	C/C	A/A
case-105	2	78	0	0	0	C/T	C/C	A/A
case-106	1	78	1	0	1	T/T	G/C	A/A
case-107	1	78	1	0	0	C/T	C/C	A/A
case-108	2	78	0	0	0	C/C	G/G	A/A
case-109	1	78	1	1	1	C/T	G/G	G/A

case-110	2	78	0	0	0	C/C	G/C	G/A
case-111	1	78	0	0	0	T/T	G/G	G/A
case-112	1	78	0	1	0	C/T	G/C	A/A
case-113	1	78	0	0	0	C/T	C/C	A/A
case-114	1	78	0	0	0	C/T	G/C	A/A
case-115	1	78	1	0	1	C/T	G/C	A/A
case-116	1	78	1	1	0	C/T	C/C	A/A
case-117	2	78	0	0	0	C/C	G/C	A/A
case-118	1	78	0	0	0	C/T	G/G	A/A
case-119	1	78	1	1	0	C/C	G/C	G/A
case-120	1	78	0	0	0	C/T	G/C	A/A
case-121	2	78	0	0	0	C/C	G/C	A/A
case-122	1	78	0	0	0	C/T	G/C	A/A
case-123	1	78	1	0	1	T/T	G/C	A/A
case-124	1	78	0	0	1	C/T	G/G	A/A
case-125	1	78	1	1	0	C/T	C/C	A/A
case-126	1	78	0	0	1	C/C	G/C	A/A
case-127	1	78	1	1	0	T/T	C/C	A/A
case-128	1	78	0	0	0	C/T	C/C	G/A
case-129	1	78	1	0	0	T/T	G/G	A/A
case-130	1	78	1	0	0	C/C	C/C	A/A
case-131	2	78	0	0	0	C/T	C/C	G/A
case-132	2	78	0	0	0	T/T	G/C	A/A
case-133	1	78	1	0	1	T/T	G/C	A/A
case-134	1	78	0	0	1	C/C	G/C	A/A
case-135	1	78	1	0	1	T/T	G/C	A/A
case-136	2	78	0	0	0	C/T	G/C	G/A
case-137	2	78	0	0	1	C/T	G/G	A/A
case-138	2	78	0	0	1	C/T	G/C	A/A
case-139	1	78	1	1	0	T/T	C/C	A/A
case-140	2	78	0	0	0	C/T	C/C	A/A
case-141	1	78	1	1	1	C/T	C/C	A/A
case-142	1	78	1	1	0	C/T	G/C	A/A
case-143	1	78	0	0	0	C/T	G/G	A/A
case-144	2	78	0	0	0	T/T	G/C	A/A
case-145	1	78	1	0	1	T/T	G/C	A/A
case-146	2	78	0	0	0	T/T	G/C	A/A

case-147	2	78	0	0	0	?	?	?
case-148	1	78	1	1	0	C/C	G/C	G/A
case-149	1	78	0	0	0	?	?	?
case-150	2	78	0	0	0	C/T	C/C	A/A
case-151	1	78	0	0	1	C/C	C/C	A/A
case-152	1	78	0	0	1	C/C	G/C	A/A
case-153	2	78	0	0	0	C/T	G/C	A/A
case-154	1	78	1	0	0	C/T	C/C	A/A
case-155	1	78	1	1	0	C/C	G/C	A/A
case-156	1	78	1	1	1	C/C	C/C	A/A
case-157	2	78	0	0	0	C/T	C/C	G/A
case-158	1	78	0	1	0	T/T	G/C	A/A
case-159	1	78	0	0	0	T/T	G/C	A/A
case-160	1	78	1	1	0	C/C	C/C	A/A
case-161	1	78	1	0	1	T/T	C/C	A/A
case-162	1	78	0	0	1	T/T	G/C	A/A
case-163	1	78	0	0	1	C/C	C/C	A/A
case-164	2	78	0	0	1	C/T	G/C	G/A
case-165	1	78	1	1	0	T/T	G/C	A/A
case-166	2	78	0	0	0	T/T	G/C	A/A
case-167	1	78	1	1	0	C/C	G/C	A/A
case-168	2	78	0	0	0	C/T	C/C	G/A
case-169	2	78	0	0	0	T/T	C/C	A/A
case-170	1	78	1	1	0	C/T	G/G	G/A
case-171	1	78	1	1	1	C/T	C/C	A/A
case-172	1	78	0	0	0	C/C	C/C	A/A
case-173	1	78	1	1	0	C/C	G/C	A/A
case-174	1	78	1	1	0	C/C	C/C	G/A
case-175	1	78	0	0	1	C/C	G/C	G/G
case-176	1	78	0	0	1	C/T	G/G	G/A
case-177	1	78	1	1	1	T/T	G/C	G/A
case-178	2	78	0	0	0	C/T	C/C	A/A
case-179	1	78	1	1	0	C/T	G/G	A/A
case-180	1	78	0	0	0	C/T	G/C	A/A
case-181	2	78	0	0	0	C/C	C/C	A/A
case-182	1	78	1	0	1	?	?	A/A
case-183	1	78	1	0	0	C/T	C/C	A/A

case-184	2	78	~~0~~	~~0~~	1	C/T	C/C	A/A
case-185	1	78	0	0	1	T/T	C/C	G/A
case-186	1	78	0	1	0	T/T	G/C	A/A
case-187	1	78	1	1	0	C/T	C/C	A/A
case-188	1	78	0	0	1	C/T	G/C	A/A
case-189	2	78	0	0	0	T/T	G/C	G/A
case-190	1	78	0	1	0	T/T	G/G	A/A
case-191	1	78	1	1	0	C/T	G/C	A/A
case-192	1	78	1	0	0	C/T	G/C	A/A
case-193	2	78	0	0	0	C/T	G/C	A/A
case-194	1	78	0	0	0	C/C	G/C	G/A
case-195	1	78	0	1	0	T/T	G/C	A/A
case-196	1	78	0	0	0	C/T	G/G	A/A
case-197	2	78	0	0	0	C/T	G/C	G/A
case-198	1	78	1	0	0	C/T	G/C	A/A
case-199	1	78	1	1	0	C/C	G/C	G/A
case-200	2	78	0	0	0	C/T	G/C	G/A
case-201	1	78	1	**0**	0	?	?	?
case-202	2	78	0	0	0	C/T	C/C	A/A
case-203	2	78	0	0	0	C/T	G/C	G/A
case-204	1	78	1	0	0	C/T	C/C	G/A
case-205	1	78	0	0	1	C/T	G/C	A/A
case-206	2	78	0	0	1	C/T	G/G	G/G
case-207	1	78	0	0	1	C/T	G/C	A/A
case-208	1	78	1	0	0	C/T	G/C	A/A
case-209	1	78	0	0	0	C/T	G/G	G/A
case-210	1	78	0	0	0	T/T	G/C	A/A
case-211	2	78	0	0	0	C/C	G/G	A/A
case-212	2	78	0	0	0	C/T	C/C	A/A
case-213	1	78	1	0	0	T/T	G/C	G/A
case-214	1	78	1	0	0	T/T	C/C	G/A
case-215	1	78	0	0	1	C/T	G/G	A/A
case-216	1	78	0	1	0	C/C	C/C	A/A
case-217	2	78	0	0	1	C/T	G/C	G/A
case-218	1	78	1	1	0	C/C	G/G	A/A
case-219	1	78	1	1	0	C/T	C/C	G/A
case-220	2	78	0	0	0	C/C	G/C	G/A

case-221	2	78	0	0	0	C/T	G/G	A/A
case-222	2	78	0	0	1	T/T	C/C	A/A
case-223	2	78	0	0	1	C/T	G/C	A/A
case-224	1	78	0	1	0	C/C	G/G	A/A
case-225	1	78	0	0	0	C/T	G/C	G/A
case-226	1	78	1	0	1	T/T	C/C	A/A
case-227	2	78	0	0	1	C/C	C/C	A/A
case-228	1	78	0	0	0	C/T	G/C	A/A
case-229	1	78	1	1	0	C/T	G/C	G/A
case-230	1	78	0	0	1	C/T	C/C	A/A
case-231	1	78	0	0	1	C/T	G/C	A/A
case-232	1	78	1	1	1	C/T	G/C	A/A
case-233	2	78	0	0	0	T/T	C/C	A/A
case-234	1	78	1	0	1	C/T	G/C	G/A
case-235	1	78	1	1	1	T/T	G/G	A/A
case-236	1	78	1	0	0	T/T	C/C	A/A
case-237	1	78	1	1	1	C/T	G/G	A/A
case-238	1	78	1	1	0	C/T	G/C	A/A
case-239	1	78	1	1	0	C/C	C/C	G/A
case-240	1	78	1	0	0	C/C	C/C	A/A
case-241	1	78	1	1	0	T/T	C/C	A/A
case-242	1	78	1	0	0	C/C	C/C	A/A
case-243	1	78	0	0	1	T/T	C/C	A/A
case-244	1	78	1	0	0	C/C	G/G	G/A
case-245	1	78	1	1	0	C/T	C/C	A/A
case-246	1	78	1	1	0	C/C	G/C	A/A
case-247	1	78	1	0	0	T/T	C/C	A/A
case-248	1	78	1	0	0	C/C	G/C	A/A
case-249	1	78	1	1	0	C/T	G/C	A/A
case-250	1	78	1	0	1	T/T	C/C	A/A
case-251	1	78	0	1	1	T/T	C/C	A/A
case-252	1	78	1	1	1	C/T	C/C	A/A
case-253	1	78	1	0	0	C/C	G/C	A/A
case-254	1	78	0	0	1	T/T	C/C	G/A
case-255	2	78	0	0	0	T/T	G/C	A/A
case-256	1	78	1	1	1	T/T	G/C	G/A
case-257	2	78	0	0	0	C/T	C/C	G/G

case-258	1	78	1	1	1	T/T	G/C	A/A
case-259	1	78	1	1	0	C/T	G/C	A/A
case-260	1	78	1	1	0	C/C	C/C	A/A
case-261	2	78	0	0	0	C/T	C/C	A/A
case-262	1	78	0	0	1	T/T	G/C	A/A
case-263	1	78	1	1	0	T/T	G/C	A/A
case-264	1	78	0	0	0	C/T	C/C	G/A
case-265	1	78	1	1	1	T/T	G/C	A/A
case-266	1	78	0	0	1	C/C	C/C	A/A
case-267	1	78	0	0	0	C/T	C/C	A/A
case-268	1	78	0	0	0	T/T	C/C	A/A
case-269	1	78	1	0	0	C/T	G/C	A/A
case-270	1	78	1	1	1	T/T	G/C	G/A
case-271	2	78	0	0	0	C/T	G/G	A/A
case-272	1	78	1	1	0	C/C	C/C	A/A
case-273	1	78	0	0	0	T/T	C/C	A/A
case-274	1	78	1	0	0	C/T	G/G	G/A
case-275	1	78	1	1	0	C/T	G/C	G/A
case-276	1	78	0	0	1	T/T	G/G	G/A
case-277	2	78	0	0	0	C/T	G/C	A/A
case-278	1	78	1	0	0	C/T	C/C	A/A
case-279	1	78	1	0	1	T/T	G/C	A/A
case-280	2	78	0	0	0	T/T	C/C	A/A
case-281	1	78	1	0	0	C/T	G/C	A/A
case-282	1	78	1	0	0	C/C	G/C	G/A
case-283	1	78	1	1	0	C/C	C/C	A/A
case-284	2	78	0	0	0	C/T	C/C	G/G
case-285	1	78	1	1	0	C/T	C/C	G/A
case-286	1	78	1	1	0	C/T	G/G	A/A
case-287	1	78	1	0	1	C/T	G/G	G/A
case-288	1	78	1	1	0	C/T	G/C	A/A
case-289	2	78	0	0	1	C/T	C/C	G/A
case-290	1	78	0	0	1	C/T	G/C	A/A
case-291	1	78	1	1	1	C/T	G/G	A/A
case-292	1	78	1	1	0	T/T	C/C	A/A
case-293	1	78	0	1	0	C/T	G/C	A/A
case-294	1	78	0	0	0	T/T	G/G	G/A

case-295	1	78	1	1	1	C/C	G/C	A/A
case-296	1	78	0	0	0	C/T	G/C	A/A
case-297	2	78	0	0	1	C/T	G/G	A/A
case-298	2	78	0	0	0	T/T	G/C	A/A
case-299	1	78	0	0	0	T/T	C/C	A/A
case-300	1	78	1	1	0	C/T	G/G	A/A
case-301	1	78	0	0	0	C/C	G/G	A/A
case-302	1	78	1	1	1	C/T	C/C	G/A
case-303	2	78	0	0	1	C/C	G/C	A/A
case-304	2	78	0	0	0	T/T	G/C	A/A
case-305	1	78	0	0	0	T/T	C/C	A/A
case-306	1	78	1	1	1	C/T	G/C	G/G
case-307	1	78	1	0	0	T/T	G/G	A/A
case-308	1	78	1	0	0	T/T	G/G	A/A
case-309	2	78	0	0	1	C/T	G/G	A/A
case-310	2	78	0	0	0	T/T	G/C	A/A
case-311	1	78	1	1	0	C/T	C/C	A/A
case-312	1	78	1	1	0	T/T	G/C	A/A
case-313	1	78	1	1	0	C/T	G/C	A/A
case-314	2	78	0	0	1	C/T	G/C	A/A
case-315	1	78	1	1	0	C/T	G/G	G/G
case-316	2	78	0	0	0	C/T	G/G	A/A
case-317	1	78	1	0	0	C/T	G/C	A/A
case-318	1	78	1	1	0	T/T	G/C	A/A
case-319	2	78	0	0	1	T/T	C/C	A/A
case-320	2	78	0	0	0	C/T	G/G	A/A
case-321	2	78	0	0	1	C/C	C/C	G/G
case-322	1	78	1	1	0	C/C	G/C	A/A
case-323	1	78	0	0	0	C/T	G/G	A/A
case-324	1	78	1	1	0	C/T	G/C	A/A
case-325	1	78	1	1	1	C/T	G/C	A/A
case-326	2	78	0	0	1	C/T	G/C	A/A
case-327	1	78	1	1	0	T/T	G/G	A/A
case-328	1	78	1	1	0	T/T	G/C	A/A
case-329	2	78	0	0	1	T/T	C/C	A/A
case-330	1	78	0	0	0	C/T	C/C	A/A
case-331	1	78	1	0	0	T/T	C/C	A/A

case-332	2	78	0	0	0	T/T	C/C	A/A
case-333	1	78	1	1	0	C/T	G/C	G/A
case-334	2	78	0	0	0	T/T	G/G	A/A
case-335	1	78	1	1	0	T/T	C/C	A/A
case-336	1	78	1	0	0	T/T	G/C	G/A
case-337	1	78	1	1	1	C/T	C/C	A/A
case-338	1	78	1	1	0	C/T	G/G	A/A
case-339	1	78	1	1	0	C/C	G/C	A/A
case-340	1	78	0	0	0	C/T	G/G	G/G
case-341	1	78	0	1	1	T/T	G/C	A/A
case-342	1	78	0	0	0	C/T	C/C	G/A
case-343	2	78	0	0	1	T/T	G/C	A/A
case-344	1	78	1	0	0	T/T	C/C	A/A
case-345	2	78	0	0	0	C/T	G/G	A/A
case-346	1	78	0	0	0	C/T	G/C	A/A
case-347	1	78	1	1	0	T/T	C/C	A/A
case-348	1	78	1	1	1	C/T	G/C	A/A
case-349	1	78	0	0	0	C/C	G/G	G/A
case-350	1	78	1	1	0	T/T	G/C	A/A
case-351	1	78	1	1	0	C/T	G/C	G/A
case-352	1	78	1	1	0	C/T	G/C	A/A
case-353	1	78	1	1	0	T/T	C/C	A/A
case-354	2	78	0	0	1	C/T	C/C	A/A
case-355	2	78	0	0	0	C/C	G/C	A/A
case-356	1	78	0	0	0	C/T	G/G	A/A
case-357	2	78	0	0	0	C/C	G/C	A/A
case-358	1	78	1	1	0	C/T	G/C	G/A
case-359	1	78	0	0	0	C/T	G/G	A/A
case-360	1	78	1	0	1	T/T	G/C	A/A
case-361	1	78	1	0	1	C/T	G/G	A/A
case-362	2	78	0	0	1	T/T	G/C	A/A
case-363	2	78	0	0	0	C/T	G/C	A/A
case-364	1	78	1	0	1	C/T	G/C	G/A
case-365	1	78	1	0	0	C/T	C/C	A/A
case-366	1	78	0	0	0	C/T	G/C	A/A
case-367	1	78	1	0	0	C/T	C/C	G/A
case-368	1	78	0	0	0	C/T	C/C	G/G

case-369	1	78	1	0	0	C/C	C/C	G/A
case-370	2	78	0	0	0	T/T	C/C	A/A
case-371	2	78	0	0	0	C/C	G/G	G/A
case-372	2	78	0	0	0	T/T	C/C	A/A
case-373	1	78	1	0	0	C/T	G/C	G/A
case-374	1	78	0	0	1	T/T	G/C	G/G
case-375	1	78	1	1	0	C/T	G/C	A/A
case-376	1	78	1	0	0	T/T	G/G	A/A
case-377	2	78	0	0	0	C/C	G/G	G/G
case-378	1	78	1	1	0	T/T	C/C	G/A
case-379	1	78	1	0	0	C/T	G/C	G/A
case-380	1	78	1	0	1	T/T	C/C	A/A
case-381	1	78	1	1	0	C/T	G/C	A/A
case-382	1	78	0	0	1	C/C	C/C	A/A
case-383	2	78	0	0	0	C/T	G/C	A/A
case-384	2	78	0	0	1	C/T	C/C	G/G
case-385	1	78	1	1	0	C/T	G/C	A/A
case-386	2	78	0	0	0	T/T	G/G	G/A
case-387	1	78	1	0	0	T/T	C/C	A/A
case-388	1	78	1	1	0	C/T	C/C	A/A
case-389	1	78	1	0	0	T/T	G/C	A/A
case-390	1	78	1	0	0	C/T	G/C	G/A
case-391	1	78	1	0	0	C/T	G/G	A/A
case-392	2	78	0	0	0	T/T	C/C	A/A
case-393	2	78	0	0	0	C/T	G/C	A/A
case-394	2	78	0	0	0	C/T	G/G	A/A
case-395	1	78	1	1	1	C/T	C/C	A/A
case-396	1	78	1	0	1	C/T	G/C	A/A
case-397	1	78	1	0	0	T/T	C/C	A/A
case-398	1	78	1	0	0	C/T	G/C	A/A
case-399	1	78	1	0	0	C/C	G/C	A/A
case-400	1	78	1	0	0	T/T	G/C	A/A
case-401	1	78	1	0	0	T/T	G/C	G/A
case-402	1	78	0	0	0	C/T	C/C	A/A
case-403	1	78	1	1	0	T/T	G/C	G/A
case-404	1	78	1	1	0	T/T	G/C	A/A
case-405	1	78	1	0	1	C/C	G/C	A/A

case-406	1	78	1	1	0	T/T	C/C	A/A
case-407	2	78	0	0	0	C/T	G/C	G/A
case-408	1	78	1	0	0	C/C	C/C	A/A
case-409	1	78	1	0	1	C/T	C/C	G/A
case-410	2	78	0	0	1	T/T	C/C	A/A
case-411	1	78	1	0	0	C/T	G/C	A/A
case-412	1	78	1	0	0	C/T	C/C	G/A
case-413	1	78	1	0	0	C/C	C/C	A/A
case-414	1	78	1	1	0	C/T	G/C	A/A
case-415	1	78	0	0	0	C/C	G/C	A/A
case-416	1	78	1	1	0	C/T	G/G	A/A
case-417	1	78	1	0	0	C/C	G/C	G/A
case-418	1	78	1	1	0	C/T	C/C	A/A
case-419	1	78	1	1	0	T/T	C/C	A/A
case-420	1	78	1	1	0	T/T	C/C	A/A
case-421	2	78	0	0	0	C/T	G/G	A/A
case-422	2	78	0	0	0	C/C	C/C	G/A
case-423	1	78	1	1	0	T/T	G/G	G/G
case-424	2	78	0	0	0	C/T	C/C	A/A
case-425	1	78	1	0	0	C/T	G/G	G/A
case-426	1	78	1	1	0	C/T	G/G	A/A
case-427	1	78	1	0	0	C/T	G/C	A/A
case-428	1	78	1	0	0	C/T	C/C	A/A
case-429	1	78	1	0	0	C/T	C/C	G/A
case-430	1	78	1	0	0	T/T	C/C	A/A
case-431	2	78	0	0	0	T/T	G/C	G/A
case-432	1	78	0	1	0	C/C	C/C	A/A
case-433	1	78	1	1	0	C/T	G/G	A/A
case-434	2	78	0	0	1	T/T	G/C	A/A
case-435	1	78	0	1	0	C/T	G/C	G/A
case-436	2	78	0	0	0	T/T	G/G	A/A
case-437	1	78	1	1	0	C/T	C/C	A/A
case-438	1	78	1	1	0	C/T	G/C	A/A
case-439	1	78	1	1	0	C/C	G/C	A/A
case-440	1	78	1	1	0	T/T	C/C	A/A
case-441	2	78	0	0	1	C/T	G/C	A/A
case-442	1	78	1	1	0	T/T	G/C	A/A

case-443	1	78	0	0	1	C/T	C/C	G/G
case-444	1	78	0	0	1	C/C	G/C	G/A
case-445	1	78	0	0	0	C/T	G/C	A/A
case-446	1	78	1	1	0	T/T	G/C	A/A
case-447	1	78	0	0	1	C/T	G/C	A/A
case-448	1	78	1	0	0	C/T	G/G	G/A
case-449	2	78	0	0	1	T/T	G/C	A/A
case-450	1	78	1	1	0	C/T	G/C	A/A
case-451	2	78	0	0	0	C/T	G/C	A/A
case-452	1	78	1	1	0	C/T	G/G	A/A
case-453	1	78	0	0	1	T/T	C/C	A/A
case-454	1	78	1	0	0	T/T	G/C	A/A
case-455	1	78	1	1	0	C/C	C/C	A/A
case-456	1	78	0	1	0	C/T	G/C	A/A
case-457	1	78	1	1	1	C/C	G/C	A/A
case-458	2	78	0	0	1	C/C	G/C	G/A
case-459	2	78	0	0	0	C/C	G/G	G/A
case-460	1	78	1	1	0	C/C	C/C	A/A
case-461	1	78	1	0	1	C/C	C/C	G/A
case-462	1	78	1	0	1	C/T	G/C	A/A
case-463	1	78	1	0	1	C/C	G/G	G/A
case-464	1	78	1	1	1	C/T	G/C	A/A
case-465	2	78	0	0	0	C/T	G/C	A/A
case-466	2	78	0	0	0	C/T	G/C	A/A
case-467	2	78	0	0	1	C/C	G/C	A/A
case-468	1	78	0	0	0	T/T	G/G	A/A
case-469	1	78	0	0	0	T/T	G/C	A/A
case-470	1	78	1	1	0	C/C	G/C	A/A
case-471	1	78	1	1	0	C/T	C/C	A/A
case-472	1	78	1	1	0	C/T	C/C	A/A
case-473	1	78	1	0	0	C/C	G/C	G/A
case-474	1	78	0	0	0	T/T	G/C	A/A
case-475	1	78	1	1	1	C/T	G/C	A/A
case-476	1	78	1	1	0	C/T	C/C	A/A
case-477	1	78	0	0	0	C/T	C/C	A/A
case-478	1	78	1	1	0	C/C	G/C	A/A
case-479	1	78	0	0	0	T/T	G/G	A/A

case-480	2	78	0	0	0	C/T	G/C	A/A
case-481	2	78	0	0	0	C/T	C/C	A/A
case-482	2	78	0	0	1	C/T	G/G	A/A
case-483	2	78	0	0	0	C/C	G/C	G/A
case-484	2	78	0	0	0	T/T	G/C	G/A
case-485	2	78	0	0	0	C/T	C/C	G/A
case-486	2	78	0	0	0	C/T	G/C	A/A
case-487	1	78	0	0	0	T/T	G/C	A/A
case-488	2	78	0	0	0	T/T	G/C	A/A
case-489	1	78	1	1	1	C/T	C/C	A/A
case-490	1	78	1	0	0	C/T	G/C	A/A
case-491	1	78	0	0	0	T/T	C/C	A/A
case-492	1	78	0	0	1	C/C	G/C	A/A
case-493	2	78	0	0	0	C/T	C/C	A/A
case-494	2	78	0	0	0	C/T	C/C	A/A
case-495	1	78	1	1	0	C/T	G/C	A/A
case-496	1	78	1	1	0	C/T	G/C	A/A
case-497	1	78	1	1	1	C/T	G/C	A/A
case-498	1	78	0	0	0	T/T	G/C	A/A
case-499	2	78	0	0	0	C/C	C/C	A/A
case-500	1	78	1	1	0	T/T	G/C	G/A
case-501	1	78	0	0	0	C/T	C/C	G/A
case-502	1	78	0	0	1	C/T	C/C	A/A
case-503	1	78	0	0	1	C/C	G/C	A/A
case-504	1	78	0	0	0	C/T	G/C	A/A
case-505	1	78	1	1	0	C/T	C/C	A/A
case-506	1	78	1	1	0	C/T	G/G	G/G
case-507	1	78	1	1	0	C/C	G/C	A/A
case-508	2	78	0	0	1	T/T	C/C	G/A
case-509	2	78	0	0	0	C/T	G/G	G/A
case-510	1	78	0	0	0	C/C	G/G	G/A
case-511	2	78	0	0	1	T/T	G/C	G/A
case-512	1	78	1	0	0	C/T	G/C	A/A
case-513	1	78	1	0	1	C/T	G/C	G/A
case-514	2	78	0	0	1	T/T	G/C	A/A
case-515	1	78	1	1	0	T/T	G/C	G/A
case-516	1	78	1	0	1	C/C	G/C	A/A

case-517	2	78	0	0	1	C/C	G/C	A/A
case-518	1	78	0	0	0	C/C	C/C	A/A
case-519	1	78	1	0	1	C/T	G/G	A/A
case-520	1	78	0	0	1	C/T	G/C	A/A
case-521	1	78	1	1	0	C/T	G/G	G/A
case-522	2	78	0	0	0	T/T	G/G	A/A
case-523	2	78	0	0	1	T/T	G/C	A/A
case-524	1	78	0	0	0	C/C	G/C	A/A
case-525	1	78	0	0	1	C/T	G/C	A/A
case-526	1	78	0	0	1	C/T	G/G	A/A
case-527	1	78	1	1	0	C/T	G/C	A/A
case-528	1	78	0	0	0	C/C	C/C	G/A
case-529	1	78	1	1	0	T/T	G/C	A/A
case-530	1	78	0	0	1	T/T	G/G	A/A
case-531	2	78	0	0	1	C/T	G/C	A/A
case-532	1	78	0	1	1	C/C	G/C	G/A
case-533	1	78	1	0	1	C/T	G/C	G/G
case-534	1	78	1	0	0	T/T	C/C	A/A
case-535	1	78	0	0	0	T/T	G/C	A/A
case-536	1	78	1	0	1	T/T	G/C	G/A
case-537	1	69	1	1	1	C/T	G/G	A/A
case-538	1	71	1	0	1	C/T	G/C	G/A
case-539	1	70	0	0	1	C/T	C/C	A/A
case-540	1	52	0	0	0	C/T	G/C	G/A
case-541	1	53	1	1	0	T/T	G/C	A/A
case-542	1	71	1	1	0	C/T	G/C	A/A
case-543	2	66	0	0	0	C/C	G/G	G/A
case-544	2	69	1	0	0	T/T	G/C	G/A
case-545	1	68	1	0	1	C/T	G/G	A/A
case-546	2	67	0	0	0	T/T	G/C	A/A
case-547	1	59	1	1	0	T/T	C/C	A/A
case-548	2	68	0	0	0	C/C	G/C	A/A
case-549	1	65	1	1	1	C/T	G/C	A/A
case-550	1	65	0	0	0	C/T	G/G	A/A
case-551	1	53	0	0	0	C/T	G/C	A/A
case-552	1	73	0	0	0	C/T	G/C	A/A
case-553	2	58	0	0	0	T/T	G/C	A/A

case-554	1	51	0	0	0	C/T	G/C	A/A
case-555	1	74	0	0	0	T/T	G/C	A/A
case-556	2	83	0	0	1	C/T	G/C	G/A
case-557	1	73	0	0	0	C/C	C/C	A/A
case-558	2	74	0	0	0	C/T	G/C	A/A
case-559	1	80	0	0	0	C/T	G/G	A/A
case-560	2	66	0	0	1	C/C	C/C	G/A
case-561	1	68	1	1	0	T/T	G/C	A/A
case-562	2	76	0	0	0	C/T	C/C	G/A
case-563	1	75	0	0	0	C/C	G/C	A/A
case-564	1	60	0	0	0	T/T	G/G	A/A
case-565	1	69	1	1	1	C/T	G/C	A/A
case-566	1	51	1	1	0	C/T	G/G	G/A
case-567	2	62	0	0	0	T/T	C/C	A/A
case-568	2	63	0	0	1	C/T	G/C	A/A
case-569	1	69	0	0	0	T/T	G/G	A/A
case-570	1	73	0	0	0	T/T	G/C	A/A
case-571	1	69	1	1	0	C/T	G/C	A/A
case-572	1	64	0	1	1	C/T	G/C	A/A
case-573	1	56	1	0	1	C/C	G/C	A/A
case-574	1	60	1	1	0	T/T	G/C	A/A
case-575	1	70	0	0	0	C/T	C/C	G/A
case-576	2	77	0	0	0	C/C	G/C	A/A
case-577	2	78	0	0	0	C/T	C/C	A/A
case-578	1	68	1	1	0	C/T	C/C	A/A
case-579	1	65	1	1	0	C/T	C/C	A/A
case-580	1	47	1	1	0	T/T	G/C	A/A
case-581	2	66	0	0	1	C/T	C/C	A/A
case-582	2	69	0	0	1	C/T	G/C	A/A
case-583	1	60	0	0	0	T/T	C/C	A/A
case-584	1	57	1	1	1	C/T	G/C	G/A
case-585	1	74	0	0	0	C/C	C/C	A/A
case-586	1	66	1	0	1	T/T	C/C	A/A
case-587	1	65	1	0	0	C/T	C/C	A/A
case-588	2	66	0	0	0	T/T	G/C	G/A
case-589	1	66	1	1	1	C/T	G/G	A/A
case-590	1	61	1	1	1	C/C	C/C	A/A

case-591	1	68	1	0	0	C/C	C/C	G/A
case-592	1	66	1	1	0	C/C	C/C	A/A
case-593	1	76	0	0	0	C/T	C/C	G/A
case-594	2	53	0	0	0	C/T	C/C	A/A
case-595	2	64	0	0	1	C/T	C/C	A/A
case-596	2	73	0	0	1	T/T	G/G	A/A
case-597	1	77	0	0	0	C/T	G/G	A/A
case-598	1	58	1	1	0	T/T	C/C	A/A
case-599	2	63	0	0	0	T/T	G/C	G/A
case-600	1	54	1	1	0	T/T	G/C	A/A
case-601	1	51	0	1	0	C/T	G/C	G/A
case-602	1	52	1	0	0	C/T	C/C	G/G
case-603	1	56	0	0	1	C/C	C/C	A/A
case-604	1	52	1	1	0	C/T	G/C	A/A
case-605	1	70	0	0	0	T/T	C/C	A/A
case-606	1	52	1	1	1	T/T	G/C	A/A
case-607	1	74	1	1	0	T/T	C/C	A/A
case-608	1	52	0	0	0	?	?	?
case-609	1	76	1	1	1	?	?	?
case-610	1	65	1	1	0	C/C	G/C	A/A
case-611	1	57	1	0	1	C/T	C/C	A/A
case-612	1	72	1	0	0	T/T	G/C	G/A
case-613	1	49	1	1	0	T/T	C/C	G/A
case-614	1	57	1	1	0	C/T	C/C	A/A
case-615	1	53	0	0	0	?	?	?
case-616	1	62	1	0	0	C/T	C/C	G/G
case-617	2	61	0	0	0	C/C	G/C	A/A
case-618	1	53	1	1	0	C/T	G/G	A/A
case-619	1	50	1	1	0	?	?	?
case-620	1	54	1	1	0	?	?	?
case-621	2	69	0	0	0	T/T	G/C	A/A
case-622	1	62	1	1	1	C/T	C/C	A/A
case-623	1	65	1	0	1	T/T	C/C	G/A
case-624	1	59	1	0	0	C/T	G/C	G/A
case-625	2	66	0	0	0	C/T	G/C	A/A
case-626	1	54	1	0	0	?	?	?
case-627	1	68	0	1	0	T/T	G/C	A/A

case-628	1	62	0	0	1	C/C	G/C	A/A
case-629	1	66	1	0	1	C/T	G/C	A/A
case-630	1	53	1	1	0	C/C	C/C	A/A
case-631	1	64	0	1	0	C/T	G/G	G/A
case-632	1	70	1	0	1	C/C	C/C	A/A
case-633	1	55	1	1	0	C/T	G/C	A/A
case-634	1	54	1	0	1	C/T	C/C	A/A
case-635	1	64	1	1	0	C/C	G/C	G/A
case-636	1	54	1	0	0	C/C	G/G	A/A
case-637	1	65	1	0	1	C/C	C/C	A/A
case-638	2	64	0	0	1	C/T	G/C	A/A
case-639	1	65	1	0	0	C/T	G/C	G/A
case-640	1	54	1	0	0	C/T	G/C	G/A
case-641	1	62	1	0	0	C/C	C/C	G/A
case-642	1	50	1	0	0	T/T	G/C	A/A
case-643	1	64	1	1	0	C/C	C/C	A/A
case-644	1	57	1	1	0	C/T	C/C	A/A
case-645	2	50	0	0	0	C/T	G/C	A/A
case-646	2	65	0	0	0	C/T	C/C	A/A
case-647	1	52	1	0	1	C/T	C/C	G/A
case-648	1	64	0	0	0	T/T	C/C	A/A
case-649	1	62	1	1	0	C/T	C/C	G/A
case-650	1	70	1	0	0	T/T	C/C	A/A
case-651	1	68	1	1	1	C/T	G/C	A/A
case-652	1	63	1	0	0	?	?	?
case-653	1	54	0	1	0	C/T	C/C	A/A
case-654	1	69	1	1	0	C/T	G/C	A/A
case-655	1	63	1	1	0	T/T	G/C	G/A
case-656	1	52	0	1	0	C/T	G/C	G/A
case-657	1	83	1	0	0	C/T	G/C	A/A
case-658	2	56	0	0	0	T/T	C/C	A/A
case-659	1	64	1	1	0	C/T	G/C	G/G
case-660	1	62	1	1	0	C/T	G/C	A/A
case-661	2	62	1	0	0	C/C	C/C	A/A
case-662	2	53	0	0	1	T/T	G/C	G/A
case-663	2	63	1	0	0	C/T	G/C	A/A
case-664	2	50	0	0	0	C/T	C/C	A/A

case-665	1	63	0	0	0	C/T	G/C	G/A
case-666	1	59	0	0	0	T/T	G/C	A/A
case-667	1	51	1	0	0	T/T	C/C	A/A
case-668	1	69	1	0	1	C/T	G/C	G/A
case-669	2	64	0	0	1	T/T	C/C	G/A
case-670	2	72	0	0	1	C/T	G/C	A/A
case-671	1	67	1	0	0	T/T	G/C	A/A
case-672	1	53	0	0	0	C/T	G/G	G/G
case-673	1	57	1	1	0	C/T	C/C	A/A
case-674	1	67	1	1	0	C/T	C/C	G/A
case-675	1	48	1	0	0	C/T	G/C	A/A
case-676	1	69	1	1	0	C/C	G/C	G/A
case-677	1	65	1	1	0	T/T	G/C	A/A
case-678	1	53	0	1	0	T/T	C/C	A/A
case-679	1	65	1	0	0	T/T	C/C	A/A
case-680	1	54	1	0	1	C/T	G/C	A/A
case-681	1	62	0	0	0	C/T	G/C	A/A
case-682	1	47	1	0	1	T/T	G/C	A/A
case-683	1	59	1	1	0	C/C	G/C	A/A
case-684	1	76	1	0	0	C/T	G/C	A/A
case-685	1	50	1	0	0	C/C	G/C	G/A
case-686	1	49	1	1	0	C/T	G/G	G/A
case-687	1	76	1	0	1	T/T	C/C	A/A
case-688	1	76	0	0	0	C/T	C/C	A/A
case-689	1	55	1	0	0	C/C	G/C	A/A
case-690	1	69	0	1	0	C/T	G/C	A/A
case-691	1	61	1	0	0	C/T	G/G	A/A
case-692	2	50	0	0	0	C/T	G/C	G/A
case-693	1	57	1	0	0	C/T	C/C	A/A
case-694	1	67	1	0	1	T/T	G/C	A/A
case-695	1	72	0	0	0	C/C	G/C	A/A
case-696	1	65	0	1	0	C/T	C/C	A/A
case-697	1	49	1	0	0	T/T	G/C	A/A
case-698	1	48	0	0	0	C/T	C/C	A/A
case-699	2	64	0	0	1	C/C	C/C	A/A
case-700	2	63	0	0	0	T/T	G/C	A/A
case-701	1	74	1	0	0	C/T	G/C	G/A

case-702	1	68	0	0	0	T/T	G/C	A/A
case-703	1	62	0	0	0	C/T	G/C	G/A
case-704	1	49	1	1	0	C/T	C/C	A/A
case-705	1	72	0	0	0	T/T	G/C	A/A
case-706	1	50	1	1	0	C/C	C/C	A/A
case-707	1	69	1	1	0	C/C	G/C	G/A
case-708	2	67	0	0	0	C/C	C/C	G/A
case-709	1	58	1	0	0	T/T	G/C	A/A
case-710	1	51	1	0	0	C/C	G/C	A/A
case-711	1	71	0	0	0	C/T	G/C	G/A
case-712	1	75	0	0	0	T/T	C/C	A/A
case-713	1	58	1	1	1	C/T	C/C	A/A
case-714	1	68	1	0	0	C/C	G/C	A/A
case-715	1	57	1	0	0	C/T	G/G	A/A
case-716	1	59	0	0	1	T/T	G/C	A/A
case-717	1	62	1	1	0	T/T	C/C	A/A
case-718	1	47	1	0	1	T/T	G/C	A/A
case-719	1	55	1	1	1	C/T	G/C	A/A
case-720	1	42	1	1	0	C/T	G/C	A/A
case-721	2	54	0	0	0	C/T	C/C	G/A
case-722	1	51	1	0	0	T/T	G/C	A/A
case-723	1	63	1	1	0	C/T	G/C	A/A
case-724	1	79	1	0	0	C/T	C/C	G/A
case-725	1	52	1	1	0	T/T	G/C	A/A
case-726	1	67	0	0	0	C/C	G/C	A/A
case-727	2	55	0	0	0	C/T	G/C	A/A
case-728	1	62	1	0	0	T/T	G/C	A/A
case-729	1	55	0	0	0	C/T	G/C	A/A
case-730	1	66	1	0	1	T/T	G/C	A/A
case-731	2	64	0	0	1	T/T	C/C	G/A
case-732	1	56	1	0	0	C/C	C/C	G/A
case-733	1	76	1	0	1	?	?	?
case-734	1	58	1	0	0	T/T	C/C	A/A
case-735	1	71	1	0	0	C/T	G/C	G/G
case-736	2	68	0	0	0	C/T	C/C	A/A
case-737	1	67	1	0	0	C/C	G/C	G/A
case-738	1	50	1	1	0	C/T	C/C	A/A

case-739	1	53	1	0	1	C/T	C/C	A/A
case-740	1	60	1	0	0	C/C	C/C	A/A
case-741	1	53	1	1	0	C/T	C/C	A/A
case-742	1	71	1	0	0	T/T	C/C	A/A
case-743	1	63	0	0	0	C/C	G/G	A/A
case-744	1	67	1	0	0	C/T	G/C	A/A
case-745	1	53	1	1	1	C/C	C/C	A/A
case-746	1	51	1	0	0	C/C	G/C	A/A
case-747	1	61	1	1	1	T/T	G/C	A/A
case-748	1	57	1	0	0	C/T	G/C	A/A
case-749	1	86	1	1	0	C/T	C/C	G/A
case-750	1	66	1	0	0	T/T	C/C	A/A
case-751	1	60	1	0	0	C/C	G/G	A/A
case-752	1	50	1	0	0	T/T	G/C	A/A
case-753	1	64	0	0	1	T/T	G/C	G/A
case-754	1	72	1	1	0	C/T	G/C	A/A
case-755	1	62	1	0	0	T/T	C/C	A/A
case-756	1	76	0	0	0	C/C	G/C	A/A
case-757	1	67	0	0	0	C/T	G/C	A/A
case-758	1	63	1	1	0	T/T	G/C	G/A
case-759	2	47	0	0	0	C/T	C/C	A/A
case-760	1	45	1	1	0	C/T	C/C	A/A
case-761	1	67	1	0	0	?	?	?
case-762	1	49	1	1	0	C/T	G/C	A/A
case-763	1	68	0	0	1	C/T	C/C	G/A
case-764	1	64	1	0	1	T/T	G/G	G/A
case-765	1	59	0	0	1	T/T	C/C	A/A
case-766	2	63	0	0	0	C/T	C/C	A/A
case-767	1	54	1	1	0	C/T	G/G	A/A
case-768	1	60	0	0	1	C/C	G/C	G/A
case-769	1	62	1	0	0	C/T	G/C	A/A
case-770	1	60	1	1	0	C/C	G/C	G/G
case-771	1	67	0	0	0	T/T	G/C	G/A
case-772	1	55	1	0	0	C/T	C/C	G/A
case-773	1	63	1	0	0	C/T	G/C	A/A
case-774	1	60	1	0	0	?	?	?
case-775	2	55	0	0	1	C/T	G/C	A/A

case-776	1	52	0	1	1	T/T	C/C	A/A
case-777	2	67	0	0	0	C/T	C/C	A/A
case-778	1	71	1	1	0	?	?	?
case-779	2	57	0	0	0	T/T	G/C	G/A
case-780	1	69	0	0	1	C/T	G/C	G/A
case-781	1	50	0	0	1	T/T	G/G	A/A
case-782	1	55	1	0	0	C/T	G/C	G/G
case-783	1	54	1	1	0	T/T	G/C	A/A
case-784	2	68	0	0	0	C/T	G/G	A/A
case-785	1	46	1	0	0	C/T	G/C	A/A
case-786	2	61	0	0	0	C/T	G/C	G/A
case-787	1	61	0	0	0	C/T	C/C	A/A
case-788	1	62	0	0	0	C/T	C/C	A/A
case-789	1	47	0	0	0	C/T	C/C	G/A
case-790	1	44	1	1	0	C/T	G/C	A/A
case-791	1	66	1	0	0	C/C	G/G	G/A
case-792	1	59	1	1	0	C/C	C/C	G/A
case-793	1	64	1	1	0	?	?	?
case-794	1	69	1	1	1	T/T	C/C	A/A
case-795	1	59	1	0	0	T/T	C/C	G/G
case-796	2	77	0	0	0	?	?	?
case-797	1	58	1	1	0	C/T	G/C	A/A
case-798	1	64	0	0	0	C/T	C/C	G/A
case-799	1	76	0	0	0	C/T	C/C	A/A
case-800	1	50	1	0	0	?	?	?
case-801	1	69	0	0	0	C/T	G/C	A/A
case-802	1	51	1	0	0	C/C	G/G	G/A
case-803	1	68	1	0	1	T/T	G/C	A/A
case-804	1	78	1	0	0	T/T	C/C	A/A
case-805	1	54	1	1	0	C/T	C/C	A/A
case-806	1	59	1	1	0	T/T	G/C	G/G
case-807	1	70	1	0	0	?	?	?
case-808	1	48	1	1	0	C/T	C/C	A/A
case-809	1	67	1	0	0	C/C	C/C	A/A
case-810	1	64	0	0	0	C/T	G/C	A/A
case-811	1	62	0	0	0	C/T	G/C	A/A
case-812	1	63	1	0	0	T/T	G/C	G/A

case-813	1	41	1	0	1	?	?	?
case-814	1	50	1	1	0	?	?	?
case-815	2	64	1	0	1	C/T	G/C	G/G
case-816	2	48	0	0	0	C/T	G/G	A/A
case-817	1	79	0	0	0	?	?	?
case-818	1	61	0	0	1	?	?	?
case-819	1	57	1	0	1	C/T	C/C	G/G
case-820	1	59	1	0	0	T/T	G/C	A/A
case-821	2	64	0	0	1	T/T	C/C	G/G
case-822	1	59	1	0	0	T/T	G/C	A/A
case-823	1	59	1	0	0	C/T	C/C	A/A
case-824	1	59	1	0	0	T/T	G/C	A/A
case-825	1	49	1	0	0	?	?	?
case-826	1	67	0	0	0	C/T	C/C	A/A
case-827	1	49	1	0	0	T/T	C/C	G/A
case-828	1	72	0	0	0	T/T	C/C	G/A
case-829	2	66	0	0	0	C/C	G/C	G/A
control-001	1	73	0	0	0	C/T	G/G	A/A
control-002	1	57	0	1	0	C/T	G/C	A/A
control-003	1	67	0	0	0	C/T	C/C	G/G
control-004	1	60	1	0	0	C/C	C/C	A/A
control-005	1	61	0	1	0	T/T	G/C	A/A
control-006	1	62	0	0	1	C/T	C/C	G/A
control-007	1	64	1	1	0	T/T	G/G	G/A
control-008	1	60	0	0	0	C/C	C/C	G/A
control-009	1	71	1	0	0	C/C	G/C	A/A
control-010	1	55	0	0	0	T/T	G/C	G/A
control-011	1	58	0	0	1	C/C	G/G	A/A
control-012	1	58	0	0	1	T/T	C/C	A/A
control-013	1	76	0	0	1	T/T	G/C	A/A
control-014	1	45	0	0	1	T/T	G/C	A/A
control-015	1	52	0	0	1	C/T	G/C	A/A
control-016	1	46	0	0	1	C/T	C/C	A/A
control-017	1	53	0	0	0	C/T	G/C	A/A
control-018	1	58	0	1	0	C/T	G/C	G/A
control-019	1	69	0	0	1	C/T	G/C	A/A
control-020	1	74	0	0	0	T/T	G/C	A/A

control-021	1	49	0	0	0	T/T	C/C	A/A
control-022	1	80	0	0	0	C/T	G/C	A/A
control-023	1	72	1	0	1	T/T	G/G	A/A
control-024	1	67	0	0	1	C/T	C/C	G/A
control-025	1	58	1	1	1	C/C	G/C	A/A
control-026	1	65	1	1	1	C/C	G/C	G/A
control-027	1	52	0	0	0	T/T	G/G	G/A
control-028	1	67	0	0	1	T/T	G/C	A/A
control-029	1	51	1	1	1	C/T	G/C	A/A
control-030	1	57	0	0	1	C/T	G/C	G/A
control-031	1	62	1	0	0	T/T	C/C	A/A
control-032	1	54	0	0	1	C/T	C/C	A/A
control-033	1	52	1	0	0	T/T	C/C	A/A
control-034	1	50	0	0	1	T/T	C/C	A/A
control-035	1	49	1	1	0	C/T	C/C	A/A
control-036	1	44	0	0	1	C/T	G/C	A/A
control-037	1	61	0	0	0	T/T	G/G	G/G
control-038	1	46	1	0	1	C/T	C/C	A/A
control-039	1	64	1	0	0	C/T	G/C	A/A
control-040	1	75	0	0	1	C/T	G/C	G/G
control-041	1	52	0	1	0	C/C	C/C	A/A
control-042	1	44	0	0	1	C/C	G/C	A/A
control-043	1	50	0	0	0	C/T	G/C	A/A
control-044	1	52	0	0	1	C/C	C/C	A/A
control-045	2	51	0	0	1	C/T	G/G	A/A
control-046	1	75	0	0	1	T/T	G/C	G/A
control-047	2	66	0	0	0	T/T	G/C	G/A
control-048	2	53	0	0	0	C/T	G/C	A/A
control-049	1	63	0	0	0	C/T	G/C	A/A
control-050	1	75	1	1	0	?	?	?
control-051	1	43	0	0	1	C/C	G/G	A/A
control-052	1	52	0	0	0	T/T	G/C	A/A
control-053	1	65	1	0	1	C/C	G/C	A/A
control-054	1	52	0	0	1	T/T	G/G	A/A
control-055	2	66	0	0	1	C/T	G/G	G/A
control-056	1	61	1	0	1	T/T	G/C	A/A
control-057	2	59	0	0	0	T/T	G/C	A/A

control-058	1	81	1	0	0	T/T	G/C	A/A
control-059	1	56	0	0	1	T/T	C/C	A/A
control-060	2	47	0	0	1	C/T	G/C	A/A
control-061	1	67	0	0	0	C/T	C/C	A/A
control-062	1	55	1	1	0	C/T	G/C	A/A
control-063	2	57	0	0	0	T/T	C/C	A/A
control-064	2	68	0	0	0	C/T	C/C	G/A
control-065	1	68	0	0	0	C/T	C/C	A/A
control-066	1	61	0	0	0	T/T	C/C	A/A
control-067	2	67	0	0	1	C/T	C/C	G/A
control-068	2	70	0	0	0	T/T	G/C	G/A
control-069	2	60	0	0	1	C/T	G/C	A/A
control-070	1	67	1	1	0	T/T	C/C	A/A
control-071	2	52	0	0	1	C/T	G/C	G/A
control-072	2	47	0	0	1	C/C	G/C	G/A
control-073	1	66	0	0	0	C/T	G/C	A/A
control-074	2	59	0	0	1	C/C	G/C	G/A
control-075	2	70	0	0	1	C/T	G/C	G/A
control-076	1	67	0	0	0	C/T	C/C	A/A
control-077	1	53	0	0	1	C/T	C/C	G/A
control-078	2	65	0	0	1	C/T	C/C	A/A
control-079	2	61	0	0	0	C/T	G/C	G/A
control-080	2	66	0	0	1	C/C	C/C	G/A
control-081	1	58	0	0	0	T/T	C/C	A/A
control-082	2	66	0	0	1	T/T	C/C	A/A
control-083	1	49	0	0	0	C/C	G/C	A/A
control-084	2	67	0	0	0	C/T	G/C	A/A
control-085	2	66	0	0	0	C/C	G/C	A/A
control-086	2	76	0	0	1	C/T	C/C	A/A
control-087	1	53	0	0	0	T/T	G/C	A/A
control-088	2	60	0	0	0	C/T	G/C	G/A
control-089	1	65	0	0	1	C/T	C/C	G/A
control-090	1	46	0	0	1	C/T	C/C	A/A
control-091	1	55	0	0	0	T/T	C/C	A/A
control-092	1	56	1	1	1	T/T	G/G	A/A
control-093	2	61	0	0	1	C/T	G/G	A/A
control-094	1	53	0	0	1	C/T	G/C	A/A

control-095	1	67	0	0	1	C/T	G/G	G/A
control-096	1	74	0	0	1	T/T	G/G	G/A
control-097	1	52	0	0	1	C/T	C/C	G/A
control-098	2	62	0	0	0	C/T	C/C	G/A
control-099	1	74	0	0	1	C/T	G/C	A/A
control-100	1	64	0	0	1	C/T	C/C	A/A
control-101	1	68	0	0	0	T/T	G/C	A/A
control-102	2	59	0	0	0	C/T	G/C	A/A
control-103	2	61	0	0	0	C/T	C/C	A/A
control-104	2	59	0	0	1	C/T	C/C	A/A
control-105	1	58	1	0	0	T/T	G/C	A/A
control-106	2	61	0	0	0	C/T	C/C	A/A
control-107	1	61	0	1	1	T/T	G/C	A/A
control-108	1	57	0	0	0	C/C	G/G	G/A
control-109	2	63	0	0	1	C/T	G/G	A/A
control-110	1	69	0	0	1	C/T	G/C	A/A
control-111	1	53	0	0	0	C/T	C/C	A/A
control-112	1	57	0	0	0	C/T	C/C	G/G
control-113	1	60	0	0	1	C/T	G/C	G/A
control-114	1	69	0	0	1	T/T	C/C	A/A
control-115	1	58	0	0	1	C/T	G/C	A/A
control-116	2	53	0	0	0	C/C	G/C	A/A
control-117	1	63	0	0	0	T/T	C/C	A/A
control-118	1	66	1	0	0	T/T	G/C	A/A
control-119	1	65	1	1	0	T/T	G/C	G/G
control-120	1	68	0	0	0	T/T	G/C	G/A
control-121	2	63	0	0	0	T/T	C/C	A/A
control-122	2	67	0	0	1	C/T	G/G	A/A
control-123	2	70	0	0	0	C/T	G/C	A/A
control-124	1	76	0	0	0	C/T	G/C	A/A
control-125	2	57	0	0	0	C/C	C/C	A/A
control-126	2	50	0	0	1	T/T	G/C	A/A
control-127	1	60	0	0	0	C/T	G/C	A/A
control-128	1	56	0	0	1	C/T	G/C	A/A
control-129	2	71	0	0	1	C/T	G/C	A/A
control-130	1	53	0	0	0	T/T	G/C	A/A
control-131	2	52	0	0	1	T/T	G/C	G/A

control-132	1	59	1	1	0	C/T	G/C	G/A
control-133	1	52	0	0	1	C/T	C/C	A/A
control-134	2	70	0	0	1	T/T	G/C	A/A
control-135	2	62	0	0	0	T/T	G/C	G/A
control-136	2	68	0	0	1	C/C	G/C	A/A
control-137	2	68	0	0	0	C/T	G/G	G/A
control-138	1	73	0	0	1	C/T	G/G	A/A
control-139	2	53	0	0	1	T/T	G/G	A/A
control-140	2	63	0	0	1	C/T	C/C	A/A
control-141	1	52	0	0	1	C/T	G/C	A/A
control-142	1	56	0	0	1	T/T	G/C	A/A
control-143	1	56	0	0	1	T/T	G/C	G/A
control-144	1	59	0	0	0	C/T	G/G	A/A
control-145	1	66	0	0	0	C/T	C/C	A/A
control-146	1	59	0	0	0	T/T	G/C	A/A
control-147	2	53	0	0	0	C/C	C/C	A/A
control-148	1	59	1	0	0	C/T	G/G	A/A
control-149	1	71	0	0	1	C/T	G/C	A/A
control-150	1	69	0	0	1	T/T	C/C	A/A
control-151	1	68	0	0	0	C/T	G/G	G/G
control-152	1	50	0	0	0	C/C	G/G	A/A
control-153	1	60	0	0	0	C/T	G/C	A/A
control-154	1	67	0	0	1	C/C	G/C	A/A
control-155	1	53	1	0	1	C/C	G/C	G/A
control-156	1	56	0	0	0	C/C	G/C	A/A
control-157	1	57	0	0	1	T/T	G/C	G/A
control-158	1	74	0	0	1	C/T	G/G	A/A
control-159	1	56	0	0	1	C/T	C/C	A/A
control-160	2	71	0	0	0	C/T	G/C	A/A
control-161	1	62	0	0	1	C/C	G/C	A/A
control-162	1	55	1	0	0	C/T	G/C	A/A
control-163	1	67	0	0	1	C/T	G/C	A/A
control-164	2	70	0	0	1	C/T	C/C	A/A
control-165	1	60	1	0	1	T/T	C/C	A/A
control-166	1	76	0	0	0	T/T	G/C	A/A
control-167	1	63	1	0	1	C/T	G/C	A/A
control-168	1	60	1	0	1	C/T	C/C	G/A

control-169	1	81	0	0	1	C/C	G/C	G/A
control-170	1	65	0	0	1	T/T	C/C	A/A
control-171	2	63	0	0	1	C/C	C/C	A/A
control-172	1	57	1	0	0	C/C	C/C	A/A
control-173	2	59	0	0	1	T/T	G/C	G/A
control-174	2	68	0	0	1	T/T	C/C	G/A
control-175	1	65	0	0	0	C/T	G/C	A/A
control-176	1	72	0	0	0	C/T	G/C	A/A
control-177	1	63	1	0	0	C/T	G/C	A/A
control-178	1	65	0	0	0	C/C	C/C	G/A
control-179	2	60	0	0	1	C/T	C/C	A/A
control-180	1	64	0	0	0	C/C	G/C	A/A
control-181	1	66	1	0	1	C/T	G/C	A/A
control-182	2	51	0	0	1	C/T	C/C	A/A
control-183	1	60	1	0	0	T/T	C/C	G/A
control-184	1	55	1	0	1	C/T	G/G	A/A
control-185	1	66	1	0	1	C/T	G/C	A/A
control-186	1	63	0	0	1	C/C	G/C	G/A
control-187	1	69	1	1	0	C/C	G/C	G/A
control-188	1	78	1	0	0	C/T	C/C	A/A
control-189	1	62	1	0	0	C/T	C/C	A/A
control-190	2	51	0	0	0	C/T	C/C	G/A
control-191	1	68	0	0	1	C/C	G/C	A/A
control-192	2	63	0	0	0	T/T	G/C	A/A
control-193	2	60	0	0	0	T/T	G/C	A/A
control-194	1	74	0	0	0	T/T	G/C	G/A
control-195	1	74	0	0	1	C/T	C/C	A/A
control-196	1	61	0	0	1	C/T	C/C	A/A
control-197	1	65	0	0	0	C/C	C/C	A/A
control-198	1	76	0	0	1	C/C	G/C	A/A
control-199	2	65	1	0	1	C/T	C/C	G/A
control-200	1	58	0	0	0	?	?	?
control-201	1	71	0	0	0	C/T	G/C	A/A
control-202	1	81	0	0	0	C/T	C/C	A/A
control-203	1	65	1	0	1	C/T	G/C	A/A
control-204	1	68	0	0	0	C/T	C/C	A/A
control-205	1	64	1	0	1	T/T	G/C	A/A

control-206	1	70	0	0	0	C/C	G/C	A/A
control-207	1	61	0	0	0	C/T	G/C	A/A
control-208	1	86	0	0	1	C/T	G/C	G/A
control-209	1	48	1	0	1	C/T	G/C	G/A
control-210	1	59	1	0	1	?	?	?
control-211	1	62	1	0	1	T/T	G/C	A/A
control-212	2	63	0	0	1	T/T	G/C	G/A
control-213	2	71	0	0	0	T/T	G/G	A/A
control-214	1	61	1	0	0	C/T	G/C	A/A
control-215	2	66	0	0	1	T/T	G/C	G/A
control-216	1	63	1	1	1	C/T	G/G	A/A
control-217	1	55	0	0	0	C/T	C/C	A/A
control-218	1	52	1	0	1	C/C	G/C	A/A
control-219	2	81	0	0	1	T/T	C/C	G/A
control-220	1	53	1	0	1	C/C	G/G	A/A
control-221	1	64	1	0	0	T/T	C/C	A/A
control-222	1	70	1	1	1	C/T	G/G	A/A
control-223	1	60	0	0	0	T/T	C/C	A/A
control-224	2	64	0	0	0	C/C	C/C	A/A
control-225	1	67	0	0	1	C/T	G/C	G/A
control-226	1	77	1	0	1	C/T	C/C	A/A
control-227	1	74	0	0	0	C/C	G/C	G/A
control-228	1	55	1	0	0	C/C	C/C	A/A
control-229	1	72	1	0	0	T/T	G/C	A/A
control-230	1	81	0	0	1	C/T	G/G	A/A
control-231	1	64	0	1	0	C/C	G/C	A/A
control-232	1	52	0	0	0	C/T	G/G	A/A
control-233	1	59	1	0	1	C/T	C/C	A/A
control-234	1	53	0	0	1	C/T	G/C	G/A
control-235	1	55	0	0	1	C/T	G/C	A/A
control-236	1	73	0	0	1	C/C	C/C	G/A
control-237	1	79	0	0	0	C/T	G/C	A/A
control-238	1	60	1	0	1	T/T	C/C	A/A
control-239	1	68	1	0	0	C/T	G/G	A/A
control-240	1	52	1	0	0	C/T	G/C	A/A
control-241	1	61	0	0	1	C/C	G/C	G/G
control-242	1	68	1	0	0	C/T	G/C	A/A

control-243	1	62	0	0	1	C/C	C/C	A/A
control-244	1	62	1	0	1	C/T	C/C	A/A
control-245	1	59	0	0	1	C/T	C/C	A/A
control-246	1	66	1	0	1	C/C	G/C	A/A
control-247	1	58	0	0	1	C/T	G/C	G/A
control-248	1	68	0	0	1	T/T	G/C	A/A
control-249	1	63	1	1	1	C/T	G/C	A/A
control-250	1	63	0	0	1	C/T	G/C	A/A
control-251	1	65	1	0	1	C/T	C/C	G/A
control-252	1	59	1	0	1	T/T	G/C	A/A
control-253	1	40	0	0	1	C/T	G/C	A/A
control-254	1	46	1	0	1	C/C	C/C	A/A
control-255	1	63	0	0	0	T/T	C/C	A/A
control-256	1	78	0	0	1	C/T	C/C	A/A
control-257	1	60	0	0	1	C/T	G/C	G/A
control-258	2	68	0	0	1	C/T	G/C	G/A
control-259	1	49	1	0	0	T/T	G/C	A/A
control-260	1	66	0	0	1	C/T	C/C	G/A
control-261	1	75	1	0	1	T/T	C/C	A/A
control-262	2	65	0	0	1	C/T	G/C	A/A
control-263	1	73	1	0	0	C/T	G/C	G/A
control-264	1	61	1	0	1	T/T	G/C	A/A
control-265	1	55	1	0	1	C/T	G/C	A/A
control-266	1	68	0	0	0	T/T	G/C	A/A
control-267	1	52	1	0	0	T/T	G/C	A/A
control-268	1	58	0	1	1	C/T	G/G	A/A
control-269	2	60	0	0	1	T/T	G/C	A/A
control-270	1	53	1	0	0	T/T	G/C	G/A
control-271	1	64	0	0	0	C/C	G/C	A/A
control-272	1	63	0	0	1	C/T	C/C	A/A
control-273	1	56	1	0	0	?	?	?
control-274	1	63	1	0	1	C/T	G/C	G/A
control-275	1	49	1	0	1	C/C	G/C	G/A
control-276	1	56	0	0	0	C/T	C/C	G/A
control-277	1	64	1	0	0	C/C	G/C	A/A
control-278	1	59	1	0	1	C/T	C/C	A/A
control-279	1	45	1	0	0	C/T	G/G	A/A

control-280	1	60	0	0	1	C/C	G/G	G/A
control-281	1	60	1	0	0	T/T	C/C	A/A
control-282	1	68	0	0	0	C/T	G/C	G/A
control-283	1	56	1	0	1	C/T	G/C	G/A
control-284	1	51	0	0	1	T/T	C/C	A/A
control-285	1	66	1	0	1	C/T	G/G	A/A
control-286	1	68	0	0	1	T/T	C/C	A/A
control-287	1	54	1	0	1	C/T	C/C	A/A
control-288	1	64	0	0	0	C/T	C/C	A/A
control-289	1	58	1	0	1	C/C	G/C	A/A
control-290	1	59	0	0	1	C/C	G/G	A/A
control-291	1	87	0	0	1	C/C	G/G	G/A
control-292	1	60	1	0	1	C/T	C/C	G/A
control-293	1	46	0	0	1	C/T	G/C	A/A
control-294	1	51	1	0	1	T/T	C/C	G/A
control-295	1	63	0	0	0	T/T	G/C	A/A
control-296	1	63	0	0	0	C/T	G/C	G/A
control-297	1	80	0	0	0	C/C	G/C	A/A
control-298	1	49	1	1	0	T/T	G/C	G/A
control-299	1	74	0	1	0	T/T	G/C	G/A
control-300	1	61	0	0	1	C/T	G/C	A/A
control-301	1	65	0	0	1	C/T	C/C	A/A
control-302	1	51	1	0	1	C/T	G/C	G/A
control-303	1	53	1	0	1	C/T	G/C	A/A
control-304	2	69	0	0	1	C/T	C/C	A/A
control-305	1	64	0	0	0	C/T	G/C	A/A
control-306	2	48	0	0	1	C/T	G/C	A/A
control-307	1	53	1	0	0	T/T	G/C	A/A
control-308	2	67	0	0	0	C/T	C/C	A/A
control-309	1	68	0	0	0	C/T	C/C	A/A
control-310	1	61	0	0	0	C/T	C/C	A/A
control-311	1	60	1	0	0	C/T	C/C	G/A
control-312	2	56	0	0	1	C/T	G/C	A/A
control-313	1	60	1	0	1	T/T	G/C	A/A
control-314	1	63	1	0	0	C/T	G/C	A/A
control-315	1	50	0	0	1	C/C	C/C	A/A
control-316	1	63	1	0	1	C/T	G/C	G/A

control-317	2	51	0	0	0	C/T	G/G	A/A
control-318	2	51	0	0	0	C/C	C/C	A/A
control-319	1	59	0	0	1	C/T	G/C	A/A
control-320	2	61	0	0	0	C/T	G/C	A/A
control-321	1	57	0	0	1	T/T	C/C	A/A
control-322	1	71	1	0	0	T/T	C/C	A/A
control-323	2	68	0	0	0	T/T	G/C	A/A
control-324	1	67	0	0	0	C/C	C/C	A/A
control-325	1	61	1	0	0	C/C	G/C	G/A
control-326	2	54	0	0	0	C/T	C/C	A/A
control-327	1	64	0	1	0	C/T	G/G	A/A
control-328	2	67	0	0	0	C/C	C/C	A/A
control-329	1	71	1	1	1	C/T	G/C	G/A
control-330	2	59	0	0	0	C/T	G/G	A/A
control-331	2	68	0	0	1	C/C	G/C	G/A
control-332	1	60	1	0	0	C/T	C/C	G/A
control-333	2	72	0	0	1	C/T	C/C	A/A
control-334	1	65	0	0	0	T/T	G/G	G/A
control-335	1	61	1	0	1	T/T	G/G	A/A
control-336	2	63	0	0	1	T/T	C/C	A/A
control-337	1	59	1	0	0	T/T	G/C	G/A
control-338	1	63	1	0	0	C/C	G/C	A/A
control-339	1	65	1	0	1	C/C	G/G	A/A
control-340	1	53	1	0	0	C/T	G/G	A/A
control-341	1	67	0	0	0	C/T	C/C	A/A
control-342	1	60	1	0	1	T/T	G/C	G/A
control-343	1	64	1	0	1	C/T	C/C	A/A
control-344	2	69	0	0	0	T/T	G/C	A/A
control-345	2	66	0	0	0	C/T	G/C	G/A
control-346	2	59	0	0	0	C/C	C/C	A/A
control-347	1	73	0	0	1	C/T	G/G	G/G
control-348	2	70	0	0	0	C/T	C/C	G/A
control-349	2	74	0	0	1	C/T	G/C	G/A
control-350	1	62	1	0	0	C/T	G/G	A/A
control-351	2	66	0	0	0	C/T	C/C	A/A
control-352	1	74	1	0	1	C/T	C/C	A/A
control-353	1	62	1	0	0	C/T	G/C	A/A

control-354	1	69	1	0	0	C/T	G/G	A/A
control-355	2	61	0	0	0	C/T	C/C	G/A
control-356	1	47	0	0	0	T/T	G/G	G/A
control-357	1	63	0	0	0	C/T	G/C	A/A
control-358	1	60	0	0	0	C/T	C/C	G/A
control-359	2	63	0	0	1	C/T	G/C	G/A
control-360	1	71	1	0	1	C/T	G/G	G/A
control-361	1	71	0	0	1	C/C	G/C	G/A
control-362	1	64	0	0	0	T/T	G/C	A/A
control-363	1	60	1	0	1	C/C	C/C	A/A
control-364	1	58	1	0	0	C/T	C/C	A/A
control-365	1	55	1	1	0	C/T	G/C	A/A
control-366	1	58	1	0	0	T/T	G/C	G/A
control-367	1	57	0	0	1	C/T	G/C	A/A
control-368	2	49	0	0	1	C/T	G/C	G/A
control-369	2	69	0	0	0	C/C	G/C	A/A
control-370	1	60	0	0	0	T/T	G/C	G/A
control-371	1	56	1	0	0	C/T	C/C	A/A
control-372	1	66	0	0	0	C/T	G/C	A/A
control-373	1	56	0	0	0	C/C	C/C	G/A
control-374	1	62	0	0	0	T/T	G/C	A/A
control-375	1	62	0	0	1	C/T	C/C	A/A
control-376	2	65	0	0	1	C/C	G/C	A/A
control-377	2	66	0	0	0	C/T	G/C	G/A
control-378	1	61	0	0	0	C/T	C/C	A/A
control-379	1	63	0	0	0	T/T	G/C	G/A
control-380	1	66	1	0	0	C/T	G/C	G/A
control-381	1	54	1	0	1	C/T	G/G	G/A
control-382	1	69	0	0	1	C/T	C/C	A/A
control-383	1	63	0	0	0	T/T	G/C	A/A
control-384	1	46	0	0	1	C/C	G/C	A/A
control-385	1	68	0	0	0	C/T	G/G	G/A
control-386	1	69	0	0	0	T/T	G/C	G/A
control-387	1	51	1	0	1	C/T	C/C	A/A
control-388	1	66	0	0	0	T/T	G/C	A/A
control-389	1	53	1	0	0	C/C	G/C	G/A
control-390	1	65	0	0	0	T/T	G/G	A/A

control-391	1	81	0	0	1	T/T	G/C	A/A
control-392	1	72	0	0	0	C/T	G/C	G/A
control-393	1	56	0	1	1	C/T	G/C	A/A
control-394	1	57	1	0	1	T/T	G/C	A/A
control-395	1	65	1	0	0	C/T	G/C	G/A
control-396	2	63	0	0	1	C/T	G/C	A/A
control-397	2	61	0	0	0	C/T	G/C	A/A
control-398	2	71	0	0	0	C/C	C/C	A/A
control-399	1	74	0	0	0	C/C	G/C	A/A
control-400	2	53	0	0	0	C/T	G/C	A/A
control-401	1	54	1	0	0	T/T	C/C	G/A
control-402	1	64	0	0	0	C/T	G/C	A/A
control-403	2	54	0	0	0	C/T	G/C	G/A
control-404	2	57	0	0	0	C/T	C/C	A/A
control-405	2	54	0	0	1	C/C	G/C	A/A
control-406	1	63	0	0	0	C/T	C/C	A/A
control-407	1	43	0	0	0	C/C	G/G	A/A
control-408	1	59	0	0	1	C/C	C/C	A/A
control-409	2	54	0	0	0	C/T	G/C	G/A
control-410	1	58	0	0	0	C/T	C/C	A/A
control-411	2	60	0	0	0	T/T	C/C	A/A
control-412	1	67	1	0	0	T/T	C/C	A/A
control-413	2	60	0	0	1	C/T	G/G	A/A
control-414	2	72	0	0	0	C/T	G/C	A/A
control-415	1	63	1	0	1	C/T	G/C	A/A
control-416	1	60	0	0	0	C/T	C/C	G/A
control-417	2	65	0	0	1	C/T	C/C	A/A
control-418	1	58	0	0	0	T/T	G/C	A/A
control-419	1	61	0	0	1	C/T	C/C	A/A
control-420	1	58	1	0	1	C/T	C/C	A/A
control-421	1	73	1	0	0	C/T	G/G	A/A
control-422	1	84	0	0	0	C/T	C/C	A/A
control-423	1	64	0	1	0	C/T	C/C	A/A
control-424	2	70	0	0	1	C/T	C/C	G/A
control-425	1	56	0	0	1	C/T	G/C	A/A
control-426	2	56	0	0	1	C/T	G/C	G/A
control-427	2	52	0	0	1	T/T	G/C	A/A

control-428	1	58	0	0	0	T/T	G/C	A/A
control-429	1	60	0	0	0	C/T	G/C	A/A
control-430	1	62	1	0	0	C/T	G/C	A/A
control-431	1	60	1	0	0	C/T	C/C	A/A
control-432	1	79	0	0	1	C/T	G/C	A/A
control-433	1	59	1	0	0	T/T	G/C	G/A
control-434	2	71	0	0	1	T/T	G/C	A/A
control-435	1	49	0	0	0	C/T	C/C	G/A
control-436	2	64	0	0	1	C/C	G/C	G/A
control-437	1	61	1	0	0	T/T	C/C	A/A
control-438	1	63	0	0	1	T/T	C/C	A/A
control-439	2	55	0	0	0	C/T	C/C	A/A
control-440	2	59	0	0	1	C/T	C/C	A/A
control-441	1	56	0	0	0	C/T	G/C	A/A
control-442	1	58	0	0	0	C/C	G/C	A/A
control-443	2	67	0	0	0	C/T	G/G	G/A
control-444	1	74	0	1	1	T/T	C/C	A/A
control-445	2	56	0	0	0	C/T	G/G	A/A
control-446	1	60	1	0	1	T/T	C/C	G/A
control-447	2	67	0	0	0	C/T	C/C	G/A
control-448	1	59	1	0	1	C/T	C/C	A/A
control-449	1	79	1	1	0	T/T	C/C	G/A
control-450	1	64	0	0	0	C/T	G/G	A/A
control-451	1	57	0	0	0	T/T	C/C	A/A
control-452	1	60	0	0	0	C/T	C/C	A/A
control-453	1	64	0	0	1	C/T	C/C	A/A
control-454	1	47	1	0	1	C/T	G/C	G/A
control-455	1	56	1	0	1	C/C	C/C	A/A
control-456	1	51	0	0	0	C/C	C/C	G/G
control-457	1	59	0	0	1	T/T	G/G	A/A
control-458	1	80	0	0	0	C/T	G/C	A/A
control-459	1	78	0	0	1	C/T	G/C	G/A
control-460	1	60	0	0	1	C/T	C/C	G/A
control-461	2	72	0	0	0	C/T	G/C	G/A
control-462	1	53	1	0	0	C/T	C/C	A/A
control-463	1	71	0	0	1	T/T	G/C	G/A
control-464	1	64	0	0	1	T/T	G/G	A/A

control-465	1	59	0	0	1	T/T	G/C	A/A
control-466	1	71	1	0	0	C/C	C/C	G/A
control-467	1	56	1	0	0	C/T	C/C	G/A
control-468	1	59	0	0	0	C/T	G/C	A/A
control-469	2	71	0	0	0	C/C	G/C	A/A
control-470	1	70	0	0	0	C/C	C/C	A/A
control-471	2	68	0	0	1	T/T	C/C	A/A
control-472	1	81	0	0	0	C/C	G/C	A/A
control-473	1	74	0	0	0	C/T	G/C	A/A
control-474	1	60	1	1	0	C/T	G/C	A/A
control-475	1	60	1	0	1	C/T	G/C	A/A
control-476	1	62	0	0	1	T/T	C/C	A/A
control-477	1	52	0	0	0	C/T	C/C	A/A
control-478	1	55	0	0	1	T/T	G/C	G/A
control-479	1	77	0	0	0	C/T	C/C	A/A
control-480	2	70	0	0	0	C/T	G/C	G/G
control-481	1	57	0	0	0	T/T	C/C	G/A
control-482	1	65	1	0	0	T/T	G/C	A/A
control-483	1	50	0	1	1	T/T	C/C	A/A
control-484	2	52	0	0	0	C/T	G/C	A/A
control-485	1	76	1	0	0	C/C	C/C	G/G
control-486	1	66	0	0	1	C/T	G/G	A/A
control-487	2	65	0	0	0	C/T	G/G	A/A
control-488	1	57	0	0	0	C/C	G/G	A/A
control-489	2	67	0	0	1	C/C	C/C	A/A
control-490	1	62	0	0	0	C/T	C/C	A/A
control-491	1	64	1	0	1	C/T	G/C	G/A
control-492	1	76	1	0	0	C/T	G/C	A/A
control-493	1	58	0	0	0	C/T	C/C	A/A
control-494	1	64	0	0	0	C/C	C/C	G/A
control-495	1	62	0	0	1	C/T	C/C	A/A
control-496	1	66	0	0	1	C/C	C/C	A/A
control-497	1	61	0	0	1	C/C	C/C	A/A
control-498	2	49	0	0	0	C/C	G/C	A/A
control-499	1	63	1	0	0	C/T	C/C	G/G
control-500	2	53	0	0	0	C/T	G/C	A/A
control-501	1	62	1	0	1	C/T	C/C	G/A

control-502	2	59	0	0	0	C/T	C/C	A/A
control-503	2	51	0	0	1	C/C	G/C	G/A
control-504	1	69	0	0	0	C/T	C/C	A/A
control-505	1	77	1	0	0	C/T	G/C	A/A
control-506	1	48	0	0	1	T/T	C/C	A/A
control-507	1	75	0	0	1	C/T	G/G	A/A
control-508	2	65	0	0	0	C/C	G/C	A/A
control-509	2	65	0	0	0	T/T	G/G	G/A
control-510	1	62	0	0	1	C/T	C/C	A/A
control-511	2	69	0	0	1	C/T	G/C	A/A
control-512	2	65	0	0	1	C/C	G/G	A/A
control-513	1	56	1	0	0	T/T	G/C	A/A
control-514	1	53	0	0	1	C/C	C/C	G/A
control-515	1	50	1	0	0	T/T	G/C	G/A
control-516	1	58	0	0	0	T/T	C/C	A/A
control-517	2	61	0	0	0	C/T	C/C	A/A
control-518	2	59	0	0	1	C/T	G/C	A/A
control-519	2	61	0	0	0	C/T	C/C	A/A
control-520	1	46	0	0	0	T/T	G/G	A/A
control-521	1	65	0	0	0	T/T	G/C	A/A
control-522	1	44	1	0	1	C/T	C/C	A/A
control-523	1	77	0	0	0	T/T	G/C	G/A
control-524	1	74	1	0	0	T/T	G/C	G/A
control-525	1	55	0	0	1	C/T	G/G	A/A
control-526	2	67	0	0	1	C/T	G/G	A/A
control-527	2	66	0	0	0	C/T	G/C	G/A
control-528	1	58	0	0	0	T/T	C/C	A/A
control-529	1	84	0	0	0	C/T	G/C	G/G
control-530	1	63	0	0	1	C/T	G/C	A/A
control-531	1	74	0	0	1	C/C	C/C	A/A
control-532	1	76	0	0	0	C/T	C/C	G/A
control-533	2	57	0	0	0	C/T	C/C	G/A
control-534	1	73	1	0	1	C/T	G/C	A/A
control-535	1	51	0	0	1	T/T	C/C	G/A
control-536	1	69	0	0	1	T/T	G/G	A/A
control-537	1	46	0	0	0	C/T	G/G	A/A
control-538	1	59	0	0	1	C/C	C/C	A/A

control-539	1	76	0	0	0	T/T	C/C	A/A
control-540	1	64	0	0	1	T/T	G/C	A/A
control-541	2	72	0	0	0	C/C	G/C	A/A
control-542	1	52	0	0	1	C/T	C/C	G/A
control-543	1	52	1	0	0	C/T	G/C	A/A
control-544	1	80	0	0	1	C/T	G/C	G/A
control-545	1	59	0	0	0	C/T	G/C	A/A
control-546	1	70	0	0	1	C/T	C/C	A/A
control-547	1	60	0	0	1	C/T	G/C	A/A
control-548	1	84	0	0	0	T/T	G/C	A/A
control-549	1	55	0	0	1	C/C	G/C	G/A
control-550	1	63	0	0	0	C/T	C/C	A/A
control-551	1	43	1	0	0	C/T	G/C	A/A
control-552	1	68	0	0	0	T/T	C/C	A/A
control-553	2	62	0	0	0	C/T	C/C	A/A
control-554	1	41	1	0	1	C/T	G/C	A/A
control-555	1	60	1	0	0	C/T	C/C	A/A
control-556	1	41	0	0	1	C/T	C/C	A/A
control-557	1	75	1	0	1	C/T	C/C	A/A
control-558	2	62	0	0	1	T/T	C/C	A/A
control-559	2	53	0	0	1	T/T	G/C	A/A
control-560	1	69	0	0	1	T/T	G/C	A/A
control-561	2	52	0	0	1	T/T	C/C	A/A
control-562	1	73	1	0	0	C/T	G/C	A/A
control-563	2	56	0	0	0	C/T	C/C	A/A
control-564	1	56	0	0	1	C/T	G/C	A/A
control-565	1	71	1	0	1	C/C	G/G	A/A
control-566	1	71	0	0	0	C/C	G/C	A/A
control-567	1	65	1	1	0	C/T	G/C	A/A
control-568	1	62	0	0	1	C/C	G/C	G/A
control-569	2	56	0	0	0	C/C	G/G	A/A
control-570	1	56	0	0	0	T/T	G/G	A/A
control-571	1	62	1	0	1	C/T	G/C	A/A
control-572	2	51	0	0	0	C/C	G/C	A/A
control-573	1	45	0	0	1	C/T	G/G	A/A
control-574	1	64	1	0	0	C/C	C/C	A/A
control-575	2	52	0	0	1	C/C	C/C	A/A

control-576	1	53	1	0	1	T/T	G/C	A/A
control-577	1	64	1	1	1	C/T	C/C	A/A
control-578	1	71	1	1	0	C/T	G/C	A/A
control-579	1	48	1	0	1	C/T	C/C	A/A
control-580	1	57	0	0	0	C/C	C/C	G/A
control-581	1	56	0	0	1	T/T	G/C	A/A
control-582	1	55	0	0	1	C/T	G/C	A/A
control-583	1	60	0	0	1	C/T	G/G	A/A
control-584	1	61	1	0	0	C/C	C/C	A/A
control-585	2	61	0	0	0	C/T	G/C	A/A
control-586	1	58	0	0	0	C/T	C/C	A/A
control-587	1	46	1	0	1	T/T	C/C	G/A
control-588	2	51	0	0	1	C/T	G/C	A/A
control-589	1	50	1	0	1	C/T	C/C	A/A
control-590	1	64	1	1	1	T/T	G/G	A/A
control-591	2	61	0	0	0	C/C	G/C	A/A
control-592	1	74	0	0	1	C/T	G/C	A/A
control-593	1	62	0	0	0	C/C	G/C	A/A
control-594	1	59	0	1	0	C/T	C/C	A/A
control-595	1	69	0	0	0	C/T	C/C	A/A
control-596	1	62	0	0	0	C/T	G/G	A/A
control-597	1	57	0	0	1	C/C	C/C	A/A
control-598	1	56	1	0	1	C/T	G/C	A/A
control-599	1	56	1	0	0	C/C	C/C	A/A
control-600	1	53	0	0	1	T/T	G/C	A/A
control-601	2	52	0	0	0	T/T	G/C	A/A
control-602	1	55	1	0	0	C/C	G/C	A/A
control-603	1	74	1	0	1	C/C	G/C	A/A
control-604	2	61	0	0	1	T/T	C/C	A/A
control-605	2	57	0	0	1	C/C	C/C	A/A
control-606	1	64	0	0	1	T/T	G/C	A/A
control-607	1	60	0	0	1	T/T	C/C	G/A
control-608	1	43	0	0	1	C/T	G/C	A/A
control-609	1	79	0	0	1	C/C	C/C	G/A
control-610	1	65	0	1	0	C/T	C/C	A/A
control-611	1	75	0	0	1	C/T	C/C	A/A
control-612	1	52	0	0	1	T/T	C/C	A/A

control-613	2	59	0	0	0	T/T	C/C	G/A
control-614	1	48	0	0	1	C/T	C/C	A/A
control-615	1	69	0	0	1	C/T	G/C	A/A
control-616	1	68	0	0	1	C/T	G/G	A/A
control-617	1	71	0	0	0	C/T	C/C	A/A
control-618	2	55	0	0	1	T/T	C/C	A/A
control-619	1	57	1	0	1	C/T	G/C	A/A
control-620	1	62	0	0	1	C/T	C/C	A/A
control-621	1	54	0	0	0	T/T	G/G	A/A
control-622	1	59	0	0	0	C/C	G/C	A/A
control-623	2	62	0	0	1	T/T	G/C	A/A
control-624	1	59	1	1	0	T/T	C/C	A/A
control-625	1	61	1	0	1	C/T	G/C	G/A
control-626	1	66	0	0	1	C/C	C/C	A/A
control-627	1	62	0	0	0	C/T	G/C	A/A
control-628	1	44	0	0	1	C/T	C/C	G/A
control-629	1	63	0	0	1	C/T	G/C	G/A
control-630	1	43	0	0	1	C/T	C/C	A/A
control-631	2	51	0	0	1	C/T	C/C	A/A
control-632	1	59	1	0	1	T/T	C/C	A/A
control-633	1	42	0	0	1	T/T	G/C	A/A
control-634	1	66	0	0	0	T/T	C/C	G/A
control-635	1	68	0	1	0	C/T	G/C	A/A
control-636	1	77	0	0	1	T/T	G/C	A/A
control-637	2	61	0	0	1	C/T	G/C	A/A
control-638	2	61	0	0	1	C/T	G/C	A/A
control-639	1	73	0	0	1	C/C	G/C	A/A
control-640	1	47	1	0	1	C/T	C/C	A/A
control-641	1	44	1	0	1	T/T	C/C	G/A
control-642	1	66	0	0	0	T/T	C/C	G/A
control-643	2	53	0	0	1	C/T	G/C	A/A
control-644	2	53	0	0	1	C/T	G/C	A/A
control-645	1	53	0	0	1	C/T	C/C	A/A
control-646	1	76	0	0	1	C/C	G/C	A/A
control-647	1	44	0	0	0	T/T	G/G	A/A
control-648	1	61	0	0	1	C/C	G/C	G/A
control-649	1	63	0	0	0	C/T	G/C	A/A

control-650	2	52	0	0	1	T/T	G/G	A/A
control-651	2	56	0	0	1	C/C	G/G	A/A
control-652	2	56	0	0	1	C/C	G/C	A/A
control-653	1	41	0	0	0	T/T	C/C	A/A
control-654	1	74	1	0	0	T/T	C/C	G/A
control-655	2	55	0	0	0	C/C	C/C	A/A
control-656	2	48	0	0	0	C/C	G/C	A/A
control-657	1	52	0	0	0	T/T	G/C	A/A
control-658	1	57	0	0	0	C/T	C/C	A/A
control-659	1	62	1	0	0	C/T	G/C	A/A
control-660	1	67	1	0	1	T/T	G/C	A/A
control-661	1	49	1	0	1	C/T	G/C	G/A
control-662	1	60	0	1	1	C/C	G/C	G/A
control-663	1	69	1	0	0	C/T	C/C	A/A
control-664	1	59	1	0	1	T/T	G/G	A/A
control-665	1	54	1	0	0	C/C	G/C	A/A
control-666	1	48	0	0	1	C/T	G/C	A/A
control-667	1	58	0	0	0	C/C	C/C	A/A
control-668	1	58	0	0	1	T/T	C/C	G/A
control-669	1	52	0	0	0	T/T	G/C	A/A
control-670	1	51	0	0	0	T/T	G/C	A/A
control-671	1	50	0	0	1	T/T	G/C	A/A
control-672	1	57	0	0	0	T/T	G/G	A/A
control-673	1	74	1	1	0	T/T	G/C	A/A
control-674	2	54	0	0	1	C/T	C/C	A/A
control-675	2	53	0	0	0	C/T	C/C	A/A
control-676	1	55	0	0	0	C/T	G/C	A/A
control-677	2	54	0	0	0	C/T	C/C	A/A
control-678	1	59	0	0	1	T/T	G/C	G/A
control-679	1	63	1	1	1	T/T	G/C	G/A
control-680	1	48	1	0	1	T/T	G/C	A/A
control-681	1	56	1	0	0	C/T	G/C	G/A
control-682	2	61	0	0	0	C/T	G/G	A/A
control-683	2	62	0	0	0	C/T	C/C	A/A
control-684	2	60	0	0	1	C/T	G/C	A/A
control-685	1	50	1	0	1	C/T	C/C	A/A
control-686	1	55	0	0	1	C/T	C/C	A/A

control-687	1	55	1	0	1	C/T	G/C	A/A
control-688	2	61	0	0	1	C/T	C/C	A/A
control-689	2	61	0	0	0	C/T	G/C	G/A
control-690	1	67	0	0	1	C/T	C/C	G/A
control-691	2	51	0	0	1	C/T	G/G	A/A
control-692	1	69	0	0	1	T/T	C/C	A/A
control-693	2	52	0	0	1	T/T	G/C	A/A
control-694	2	56	0	0	0	T/T	G/C	A/A
control-695	1	52	0	0	1	C/T	G/C	G/A
control-696	1	75	0	0	0	T/T	G/C	A/A
control-697	1	58	0	0	0	C/C	C/C	G/A
control-698	1	66	0	0	0	T/T	G/C	G/A
control-699	1	70	1	0	1	T/T	C/C	G/A
control-700	1	49	1	0	0	C/T	G/C	A/A
control-701	1	78	0	0	0	T/T	G/G	G/A
control-702	1	62	0	0	0	C/C	G/C	A/A
control-703	1	72	0	0	0	C/T	C/C	A/A
control-704	1	65	1	0	0	T/T	C/C	A/A
control-705	1	51	0	0	0	C/T	G/C	A/A
control-706	2	49	0	0	0	C/T	C/C	G/A
control-707	1	53	0	0	0	T/T	G/C	A/A
control-708	1	54	0	0	0	C/T	C/C	A/A
control-709	1	70	1	0	1	C/T	G/C	A/A
control-710	1	41	0	0	0	C/C	C/C	A/A
control-711	1	62	1	0	0	T/T	G/C	A/A
control-712	2	61	0	0	1	C/C	C/C	G/A
control-713	1	62	0	0	0	T/T	C/C	A/A
control-714	1	52	0	0	0	C/T	G/C	A/A
control-715	1	49	0	0	0	C/C	C/C	A/A
control-716	1	48	1	0	0	T/T	C/C	G/A
control-717	1	60	0	0	0	T/T	C/C	G/A
control-718	1	53	0	1	0	T/T	G/C	G/A
control-719	1	59	1	0	1	C/C	C/C	A/A
control-720	1	71	1	0	1	T/T	C/C	A/A
control-721	1	47	0	0	0	T/T	C/C	G/A
control-722	1	52	0	0	0	C/C	C/C	A/A
control-723	1	66	0	0	1	C/T	C/C	A/A

control-724	1	59	0	0	1	C/T	G/C	A/A
control-725	1	51	0	0	0	C/T	G/C	A/A
control-726	2	53	0	0	1	T/T	C/C	A/A
control-727	1	62	0	0	1	T/T	C/C	A/A
control-728	1	78	0	0	0	C/T	G/C	G/A
control-729	1	55	1	0	0	T/T	C/C	A/A
control-730	1	69	0	0	1	C/C	C/C	A/A
control-731	1	50	1	0	0	C/T	C/C	A/A
control-732	1	71	1	0	0	C/T	G/C	A/A
control-733	1	68	1	0	1	T/T	C/C	G/A
control-734	1	71	0	0	0	C/C	C/C	A/A
control-735	1	43	0	0	0	C/C	G/C	A/A
control-736	1	66	0	0	1	C/T	G/C	A/A
control-737	2	53	0	0	0	C/T	C/C	A/A
control-738	2	61	0	0	1	C/T	G/G	A/A
control-739	1	67	0	0	1	T/T	G/C	G/A
control-740	1	73	1	0	0	C/T	G/C	A/A
control-741	2	59	0	0	0	C/C	C/C	A/A
control-742	1	55	1	0	0	C/C	G/C	A/A
control-743	2	60	0	0	0	C/T	G/G	A/A
control-744	1	63	1	0	1	C/T	G/G	A/A
control-745	1	61	1	0	1	C/T	G/C	A/A
control-746	1	62	0	0	1	T/T	G/G	G/A
control-747	1	57	1	0	1	C/T	G/C	G/A
control-748	1	68	0	0	1	C/T	C/C	A/A
control-749	1	63	1	0	0	C/T	G/C	G/A
control-750	1	62	1	0	0	C/T	G/C	A/A
control-751	1	76	0	0	0	T/T	G/C	A/A
control-752	1	66	0	0	1	T/T	C/C	A/A
control-753	1	79	1	0	0	C/T	C/C	A/A
control-754	1	66	0	0	1	C/C	G/G	A/A
control-755	1	60	1	0	0	T/T	C/C	G/A
control-756	1	52	0	0	1	C/C	C/C	G/A
control-757	1	63	0	0	1	C/T	C/C	A/A
control-758	1	59	1	0	0	C/T	G/C	G/A
control-759	1	72	1	1	0	C/C	G/C	A/A
control-760	1	52	0	0	0	C/C	C/C	A/A

control-761	2	58	0	0	1	C/C	G/C	A/A
control-762	1	79	0	0	1	C/T	C/C	A/A
control-763	1	59	0	0	0	C/T	G/C	G/A
control-764	1	67	0	0	0	C/T	C/C	A/A
control-765	1	59	0	0	1	T/T	C/C	G/A
control-766	1	78	0	0	0	C/T	C/C	G/A
control-767	1	59	0	0	1	C/T	G/C	A/A
control-768	1	47	1	0	1	C/T	G/C	A/A
control-769	1	58	0	0	0	C/T	C/C	G/A
control-770	1	70	0	0	0	C/T	C/C	A/A
control-771	1	74	1	0	0	C/C	G/G	A/A
control-772	1	58	0	0	1	C/T	G/C	G/A
control-773	2	50	0	0	0	T/T	G/C	A/A
control-774	2	50	0	0	1	C/T	G/C	G/A
control-775	1	65	1	0	1	C/C	G/C	G/A
control-776	1	59	0	0	0	C/C	G/C	A/A
control-777	2	51	0	0	1	T/T	C/C	A/A
control-778	1	77	0	0	1	C/T	G/C	A/A
control-779	2	65	0	0	1	C/T	C/C	G/A
control-780	1	73	0	0	0	C/T	C/C	A/A
control-781	2	63	0	0	0	C/T	G/C	A/A
control-782	2	53	0	0	1	C/T	C/C	A/A
control-783	1	53	0	0	1	C/C	G/C	G/A
control-784	2	59	0	0	0	C/T	G/C	G/A
control-785	1	53	0	0	0	C/C	G/C	A/A
control-786	1	68	0	0	0	T/T	G/C	A/A
control-787	1	62	0	0	1	T/T	G/C	G/A
control-788	1	55	0	0	0	T/T	G/C	A/A
control-789	1	62	0	0	1	C/T	C/C	A/A
control-790	1	57	0	0	1	C/T	C/C	A/A
control-791	1	63	0	0	0	C/T	C/C	A/A
control-792	2	68	0	0	1	C/T	G/C	A/A
control-793	1	76	0	0	1	T/T	C/C	G/A
control-794	1	66	0	0	1	T/T	G/G	G/A
control-795	1	62	0	0	1	C/T	G/C	A/A
control-796	1	69	1	0	0	C/T	G/C	G/A
control-797	1	61	1	0	1	T/T	C/C	A/A

control-798	2	78	0	0	1	C/T	C/C	A/A
control-799	2	58	0	0	0	C/T	G/C	A/A
control-800	1	73	0	0	0	C/T	G/C	G/A
control-801	2	66	0	0	1	C/T	G/C	A/A
control-802	1	46	1	1	1	C/T	G/C	G/A
control-803	1	76	1	0	0	C/T	G/C	G/A
control-804	1	57	1	0	1	C/C	C/C	G/A
control-805	1	57	1	0	0	C/T	G/C	A/A
control-806	2	65	0	0	0	C/T	G/C	G/A
control-807	1	62	0	0	0	T/T	C/C	A/A
control-808	1	60	1	1	0	C/T	G/G	A/A
control-809	1	66	1	0	0	C/T	G/C	A/A
control-810	1	84	0	0	0	C/T	C/C	A/A
control-811	2	71	0	0	0	C/T	G/C	G/A
control-812	2	74	0	0	1	C/C	G/G	G/A
control-813	2	74	0	0	0	T/T	C/C	A/A
control-814	1	54	1	0	0	T/T	C/C	G/A
control-815	1	71	1	0	1	C/T	C/C	A/A
control-816	1	53	0	0	0	T/T	C/C	A/A
control-817	2	71	0	0	0	C/T	C/C	A/A
control-818	1	78	0	0	0	C/T	C/C	A/A
control-819	2	71	0	0	1	C/C	C/C	A/A
control-820	2	64	0	0	1	C/T	G/C	A/A
control-821	1	55	0	0	1	T/T	C/C	A/A
control-822	2	60	0	0	0	T/T	G/C	A/A
control-823	1	73	1	0	1	C/T	C/C	A/A
control-824	1	64	1	1	1	C/C	G/C	A/A
control-825	2	68	0	0	1	T/T	C/C	A/A
control-826	1	68	0	0	0	C/T	G/C	A/A
control-827	1	59	1	0	1	C/T	G/C	G/A
control-828	1	76	0	0	1	C/T	G/C	A/A
control-829	1	59	1	0	0	C/T	C/C	A/A
control-830	1	75	1	0	0	C/C	G/C	A/A
control-831	1	63	1	0	0	T/T	C/C	A/A
control-832	1	53	0	0	1	T/T	G/C	A/A
control-833	1	66	1	0	1	C/T	C/C	A/A
control-834	1	62	0	1	1	T/T	C/C	A/A

control-835	1	52	0	0	0	C/C	G/C	A/A
control-836	1	81	0	0	0	C/C	G/G	A/A
control-837	1	67	0	0	0	C/T	G/C	G/A
control-838	1	54	1	0	1	C/T	G/C	A/A
control-839	2	74	0	0	0	T/T	C/C	A/A
control-840	1	55	0	0	0	C/T	C/C	A/A
control-841	2	76	0	0	1	C/T	G/C	G/A
control-842	2	69	0	0	1	C/T	G/C	G/A
control-843	1	48	1	0	1	T/T	C/C	G/A
control-844	1	51	1	0	1	C/T	C/C	A/A
control-845	2	60	0	0	1	C/T	G/G	G/A
control-846	1	58	0	1	1	C/T	C/C	G/A
control-847	1	66	0	0	1	C/T	G/C	A/A
control-848	2	54	0	0	0	C/C	G/C	A/A
control-849	1	68	0	0	0	C/T	G/G	G/A
control-850	1	67	1	0	0	C/T	G/C	G/A
control-851	1	51	0	0	0	C/C	C/C	A/A
control-852	1	59	1	1	1	C/T	G/C	G/A
control-853	1	65	0	0	0	T/T	C/C	A/A
control-854	1	78	0	0	1	C/T	G/C	A/A
control-855	2	59	0	0	0	C/T	G/C	A/A
control-856	1	60	0	0	0	T/T	C/C	A/A
control-857	1	58	1	0	0	C/T	C/C	A/A
control-858	1	56	1	0	0	C/T	G/C	A/A
control-859	1	73	0	0	0	C/T	G/C	A/A
control-860	1	57	1	0	1	T/T	G/C	A/A
control-861	1	64	1	0	0	C/C	G/C	A/A
control-862	1	62	1	0	1	T/T	G/C	G/A
control-863	1	66	1	0	0	C/C	G/C	G/A
control-864	1	60	0	0	1	C/T	G/C	A/A
control-865	1	52	1	0	0	C/T	G/C	G/A
control-866	1	60	1	0	0	C/T	C/C	G/A
control-867	1	61	0	0	0	C/T	C/C	A/A
control-868	1	56	1	0	0	C/C	C/C	A/A
control-869	1	70	1	0	0	T/T	G/C	A/A
control-870	1	61	1	0	1	T/T	G/G	A/A
control-871	1	53	1	0	1	C/C	G/C	A/A

control-872	1	61	1	0	1	C/T	C/C	A/A
control-873	1	68	1	0	0	C/T	G/C	G/A
control-874	1	74	0	0	0	C/T	C/C	G/G
control-875	1	66	1	1	0	C/T	C/C	A/A
control-876	1	68	1	0	1	C/T	G/G	A/A
control-877	1	61	0	1	0	C/T	C/C	A/A
control-878	1	76	0	0	0	C/T	G/C	A/A
control-879	2	74	0	0	0	C/T	G/C	A/A
control-880	1	71	0	0	0	T/T	G/C	A/A
control-881	2	65	0	0	1	C/T	G/C	G/A
control-882	2	69	0	0	0	C/T	C/C	A/A
control-883	1	58	1	1	1	C/C	G/C	A/A
control-884	2	57	0	0	1	C/T	C/C	A/A
control-885	2	61	0	0	0	C/T	C/C	G/A
control-886	2	55	0	0	1	T/T	C/C	A/A
control-887	1	49	1	1	1	T/T	G/C	G/A
control-888	2	56	0	0	1	C/C	G/C	A/A
control-889	1	51	1	1	0	C/T	G/C	A/A
control-890	1	78	0	0	0	C/T	G/C	G/A
control-891	1	77	0	0	1	T/T	C/C	A/A
control-892	2	71	0	0	1	T/T	C/C	G/A
control-893	1	70	0	0	0	T/T	G/C	A/A
control-894	1	82	0	1	1	T/T	C/C	A/A
control-895	1	78	0	0	0	C/T	G/G	A/A
control-896	1	77	0	0	1	C/C	G/C	A/A
control-897	1	74	0	0	0	C/T	C/C	A/A
control-898	1	73	1	0	0	C/T	G/G	A/A
control-899	1	72	0	0	1	C/T	C/C	A/A
control-900	1	70	0	0	0	C/T	C/C	A/A
control-901	1	61	1	1	0	T/T	G/G	A/A
control-902	1	77	0	0	0	T/T	C/C	A/A
control-903	1	77	0	0	1	C/T	C/C	A/A
control-904	2	66	0	0	1	C/T	C/C	A/A
control-905	1	82	0	0	1	C/T	C/C	G/A
control-906	1	78	0	0	0	C/C	C/C	G/A
control-907	2	71	0	0	0	C/T	G/G	A/A
control-908	1	70	0	0	1	C/C	C/C	A/A

control-909	1	69	0	0	0	C/T	C/C	A/A
control-910	1	77	0	0	0	T/T	G/C	A/A
control-911	2	71	0	0	1	T/T	G/G	A/A
control-912	1	70	0	0	0	C/T	C/C	G/A
control-913	2	61	0	0	0	C/C	G/C	A/A
control-914	2	66	0	0	1	C/T	C/C	A/A
control-915	1	77	1	0	0	T/T	G/C	A/A
control-916	1	82	1	0	0	C/T	C/C	A/A
control-917	1	77	0	0	0	C/C	G/C	G/A
control-918	1	73	0	0	1	C/T	G/G	G/A
control-919	1	71	1	0	0	C/T	C/C	G/A
control-920	1	75	0	0	0	T/T	G/C	G/A
control-921	1	73	1	0	0	C/T	G/C	G/A
control-922	1	77	1	1	1	C/T	G/G	A/A
control-923	1	73	1	1	1	C/C	G/C	A/A
control-924	2	65	0	0	0	C/T	C/C	A/A
control-925	1	65	0	0	0	T/T	G/C	A/A
control-926	2	61	0	0	1	T/T	G/G	A/A
control-927	1	60	1	0	1	T/T	C/C	A/A
control-928	1	61	1	1	0	C/T	G/C	A/A
control-929	1	68	1	0	1	T/T	G/C	A/A
control-930	1	73	0	0	0	T/T	G/C	A/A
control-931	1	70	0	0	0	C/T	G/G	A/A
control-932	1	69	0	0	0	C/T	C/C	A/A
control-933	2	66	0	0	0	C/T	G/G	A/A
control-934	1	83	0	0	0	T/T	G/G	A/A
control-935	1	81	0	0	0	C/T	G/C	A/A
control-936	1	71	0	0	1	C/C	C/C	A/A
control-937	1	68	0	0	0	T/T	G/C	A/A
control-938	2	77	0	0	0	T/T	C/C	G/A
control-939	1	69	1	1	1	C/T	C/C	A/A
control-940	2	69	0	0	1	C/T	G/C	A/A
control-941	2	65	0	0	0	C/T	G/C	A/A
control-942	1	65	1	0	0	C/T	G/C	G/A
control-943	1	61	0	0	0	C/T	G/C	G/A
control-944	1	75	0	1	0	T/T	G/C	G/A
control-945	1	75	0	0	0	T/T	G/C	G/A

control-946	1	76	0	0	0	T/T	C/C	A/A
control-947	1	75	0	0	0	C/T	C/C	G/G
control-948	2	74	0	0	1	C/T	G/G	A/A
control-949	1	78	0	0	1	C/T	G/C	A/A
control-950	1	76	0	0	1	T/T	G/C	A/A
control-951	2	68	0	0	0	C/T	G/C	A/A
control-952	1	82	1	0	0	C/C	G/G	A/A
control-953	1	70	1	0	1	T/T	G/G	G/A
control-954	1	67	0	0	1	C/T	G/C	A/A
control-955	1	75	0	0	1	T/T	G/C	G/A
control-956	1	72	1	1	0	T/T	G/C	A/A
control-957	1	81	0	0	1	T/T	G/C	A/A
control-958	1	76	0	1	0	C/C	G/C	G/A
control-959	1	60	1	0	0	C/T	C/C	A/A
control-960	1	60	1	0	1	T/T	C/C	G/A
control-961	1	59	0	0	1	T/T	C/C	G/A
control-962	1	82	1	0	0	C/T	G/C	A/A
control-963	1	79	0	0	0	C/T	G/C	A/A
control-964	1	65	0	0	1	T/T	G/C	A/A
control-965	1	75	0	1	0	T/T	G/C	A/A
control-966	1	74	0	0	0	C/T	C/C	A/A
control-967	1	66	0	0	0	C/T	C/C	A/A
control-968	2	61	0	0	0	C/C	C/C	A/A
control-969	2	60	0	0	1	C/T	C/C	A/A
control-970	1	62	0	0	1	C/T	C/C	A/A
control-971	1	78	0	0	0	T/T	G/G	A/A
control-972	1	72	0	1	1	C/T	C/C	A/A
control-973	1	61	0	1	1	T/T	G/G	G/A
control-974	1	70	0	0	1	T/T	G/C	A/A
control-975	1	58	0	1	1	T/T	G/C	A/A
control-976	1	59	0	0	0	T/T	G/C	A/A
control-977	1	73	1	0	1	T/T	G/C	A/A
control-978	1	69	1	1	0	C/T	G/C	A/A
control-979	1	76	0	0	1	C/T	G/C	A/A
control-980	1	77	0	1	0	C/T	G/C	G/A
control-981	1	77	1	0	1	C/T	G/G	A/A
control-982	1	69	0	0	0	T/T	C/C	A/A

control-983	1	80	0	0	0	C/T	G/C	A/A
control-984	1	72	1	0	1	C/T	G/G	A/A
control-985	1	74	0	0	1	C/T	C/C	A/A
control-986	1	74	0	1	0	C/T	G/C	A/A
control-987	1	65	1	1	1	C/T	C/C	A/A
control-988	1	72	1	0	1	T/T	C/C	A/A
control-989	1	73	0	1	1	T/T	C/C	A/A
control-990	1	74	1	1	1	C/C	G/C	A/A
control-991	1	71	0	1	0	C/T	G/C	G/A
control-992	1	71	0	0	1	C/T	G/C	G/A
control-993	1	68	0	0	1	C/C	G/C	A/A
control-994	1	58	0	1	1	T/T	G/C	A/A
control-995	1	64	0	1	1	C/T	C/C	G/A
control-996	1	68	0	0	0	T/T	G/G	A/A
control-997	1	80	0	0	1	C/T	G/G	G/G
control-998	1	66	0	1	1	C/T	C/C	G/A
control-999	1	77	0	1	0	T/T	G/C	A/A
control-1000	1	70	0	0	0	C/C	G/C	A/A
control-1001	1	71	0	1	1	T/T	G/G	A/A
control-1002	1	85	0	0	1	C/C	G/C	A/A
control-1003	1	77	0	0	0	C/T	G/C	A/A
control-1004	1	74	0	0	0	C/T	C/C	A/A
control-1005	1	60	1	1	0	T/T	G/C	A/A
control-1006	1	70	1	1	0	C/C	C/C	G/A
control-1007	1	60	1	1	0	C/T	G/C	A/A
control-1008	2	65	0	0	0	C/T	G/C	A/A
control-1009	1	66	1	0	1	C/T	C/C	A/A
control-1010	1	77	0	0	0	T/T	G/C	A/A
control-1011	1	70	1	1	1	T/T	C/C	A/A
control-1012	1	67	1	1	0	T/T	C/C	G/A
control-1013	1	61	1	1	1	C/C	G/C	G/A
control-1014	1	60	0	0	0	C/T	G/G	A/A
control-1015	1	70	0	1	0	T/T	C/C	G/A
control-1016	1	69	0	0	1	C/T	C/C	A/A
control-1017	2	61	0	0	0	C/C	G/C	A/A
control-1018	1	78	1	0	1	C/C	G/C	G/A
control-1019	1	75	0	0	0	C/T	G/C	A/A

control-1020	1	78	0	0	0	C/C	C/C	G/A
control-1021	1	62	1	1	1	C/T	C/C	G/A
control-1022	1	59	1	1	0	C/T	G/C	A/A
control-1023	1	72	1	0	0	C/T	C/C	A/A
control-1024	1	69	1	0	0	C/T	G/C	G/A
control-1025	1	70	0	0	0	C/T	G/C	A/A
control-1026	1	75	0	0	1	T/T	C/C	A/A
control-1027	1	71	0	0	1	C/T	C/C	A/A
control-1028	1	57	1	0	1	C/T	C/C	A/A
control-1029	2	61	0	0	1	C/T	G/G	A/A
control-1030	1	60	1	0	0	C/T	G/G	A/A
control-1031	1	76	0	0	0	C/T	C/C	A/A
control-1032	1	62	0	1	0	C/C	G/G	A/A
control-1033	1	68	1	1	0	C/T	C/C	A/A
control-1034	2	69	0	0	0	C/C	G/C	A/A
control-1035	2	59	0	0	0	C/T	G/C	G/A
control-1036	2	66	0	0	0	T/T	G/C	A/A
control-1037	1	80	0	0	0	C/T	G/C	A/A
control-1038	1	72	0	0	0	T/T	G/C	G/A
control-1039	1	71	0	0	1	C/T	G/G	A/A
control-1040	1	69	1	1	0	T/T	G/G	A/A
control-1041	1	69	0	0	0	C/C	G/G	A/A
control-1042	1	78	0	0	0	T/T	G/C	G/A
control-1043	1	71	1	1	0	C/C	G/C	A/A
control-1044	1	61	1	1	0	C/T	C/C	G/A
control-1045	1	71	1	1	1	C/T	G/C	A/A
control-1046	1	71	0	0	1	C/C	G/C	G/A
control-1047	1	74	0	0	1	C/T	G/C	A/A
control-1048	1	78	0	1	1	C/T	G/G	A/A
control-1049	1	77	0	0	0	C/T	G/C	G/A
control-1050	1	77	1	1	0	C/C	C/C	A/A
control-1051	1	74	0	1	1	T/T	G/C	A/A
control-1052	1	73	0	1	1	T/T	G/G	A/A
control-1053	1	70	0	0	1	T/T	G/G	G/A
control-1054	1	66	1	0	0	C/T	G/C	A/A
control-1055	2	61	0	0	1	T/T	G/G	G/A
control-1056	1	63	1	0	0	C/T	C/C	A/A

control-1057	2	62	0	0	0	T/T	G/C	A/A
control-1058	2	64	0	0	0	C/C	G/C	A/A
control-1059	1	61	0	0	1	C/T	G/G	A/A
control-1060	2	59	0	0	0	T/T	C/C	G/A
control-1061	2	68	0	0	0	C/C	G/C	A/A
control-1062	2	67	0	0	0	T/T	G/C	A/A
control-1063	2	70	0	0	1	C/T	C/C	A/A
control-1064	1	81	1	0	0	C/T	G/C	A/A
control-1065	1	78	0	0	1	C/T	C/C	A/A
control-1066	1	77	0	0	1	C/C	G/G	A/A
control-1067	1	77	0	0	0	C/C	G/G	A/A
control-1068	1	75	1	1	0	C/T	C/C	A/A
control-1069	1	75	1	0	1	T/T	G/C	A/A
control-1070	1	75	0	0	0	C/T	G/C	A/A
control-1071	1	74	0	0	0	T/T	C/C	A/A
control-1072	1	74	1	1	0	C/C	G/C	G/A
control-1073	1	73	0	0	1	C/T	G/C	A/A
control-1074	1	69	1	0	1	C/C	G/C	G/A
control-1075	1	71	0	0	0	C/T	C/C	A/A
control-1076	2	60	0	0	0	T/T	G/G	A/A
control-1077	1	63	1	0	1	C/C	G/G	G/A
control-1078	1	70	0	0	1	C/T	G/C	A/A
control-1079	1	74	0	0	1	C/T	C/C	G/A
control-1080	1	70	1	0	1	C/T	G/G	A/A
control-1081	2	64	0	0	0	C/T	G/C	A/A
control-1082	1	78	0	0	1	C/T	G/C	A/A
control-1083	1	78	1	1	0	C/T	G/C	A/A
control-1084	1	73	1	0	0	C/C	C/C	G/A
control-1085	1	70	1	1	0	T/T	G/G	A/A
control-1086	1	68	0	0	1	C/T	G/C	A/A
control-1087	2	61	0	0	0	C/T	C/C	G/A
control-1088	2	69	0	0	1	C/T	C/C	A/A
control-1089	1	69	1	0	0	C/C	C/C	A/A
control-1090	1	72	0	0	1	C/T	G/C	A/A
control-1091	1	62	0	0	1	C/T	G/G	A/A
control-1092	2	63	0	0	1	C/T	C/C	A/A
control-1093	1	69	0	1	1	C/T	G/G	A/A

control-1094	1	67	0	0	1	T/T	G/C	A/A
control-1095	1	72	0	0	1	T/T	C/C	A/A
control-1096	1	65	0	1	1	C/T	C/C	A/A
control-1097	1	64	0	1	1	C/C	G/C	A/A
control-1098	1	64	0	0	0	T/T	C/C	A/A
control-1099	1	78	0	0	1	C/T	C/C	A/A
control-1100	1	76	0	0	1	T/T	G/G	A/A
control-1101	2	74	0	0	0	C/C	G/C	G/A
control-1102	1	77	0	0	1	C/C	G/C	A/A
control-1103	1	73	0	1	1	C/T	G/C	G/G
control-1104	1	65	1	0	0	C/T	G/C	A/A
control-1105	2	68	0	0	1	C/T	C/C	A/A
control-1106	2	68	0	0	0	C/T	G/G	A/A
control-1107	1	73	0	0	0	C/T	G/C	A/A
control-1108	2	75	0	0	1	C/T	C/C	A/A
control-1109	1	85	0	0	1	C/T	G/C	A/A
control-1110	1	80	0	1	1	C/T	G/C	A/A
control-1111	1	80	0	0	1	C/T	G/C	A/A
control-1112	1	64	1	0	0	C/C	G/C	A/A
control-1113	1	69	0	0	1	T/T	G/C	A/A
control-1114	2	62	0	0	1	T/T	G/G	G/A
control-1115	1	65	0	0	1	T/T	G/C	G/A
control-1116	1	50	0	1	0	C/C	C/C	A/A
control-1117	2	58	0	0	1	C/T	C/C	G/A
control-1118	2	51	0	0	1	C/C	G/G	A/A
control-1119	1	64	0	0	1	T/T	G/C	G/A
control-1120	1	45	1	1	0	C/T	G/C	G/A
control-1121	1	66	0	1	1	T/T	C/C	G/A
control-1122	1	70	1	0	1	C/T	G/C	A/A
control-1123	1	51	1	0	0	C/T	C/C	A/A
control-1124	1	44	1	0	0	T/T	C/C	G/A
control-1125	2	46	0	0	0	T/T	C/C	A/A
control-1126	1	47	1	1	1	C/T	G/C	A/A
control-1127	2	58	0	0	1	T/T	C/C	A/A
control-1128	1	50	0	1	0	C/C	G/C	G/A
control-1129	1	61	1	1	1	T/T	G/C	A/A
control-1130	1	58	1	0	1	C/T	G/G	G/G

control-1131	2	56	0	0	0	C/C	G/C	A/A
control-1132	1	59	0	0	1	C/T	G/G	A/A
control-1133	1	51	1	1	1	C/T	G/C	A/A
control-1134	2	51	0	0	0	C/C	C/C	A/A
control-1135	1	58	1	1	0	C/T	G/G	A/A
control-1136	1	58	1	1	1	C/T	G/C	G/A
control-1137	1	63	0	0	1	C/T	G/C	G/A
control-1138	2	56	0	0	0	T/T	G/C	A/A
control-1139	1	58	1	1	0	C/T	C/C	A/A
control-1140	1	59	1	0	0	C/T	G/C	A/A
control-1141	1	54	1	1	1	T/T	C/C	G/A
control-1142	1	57	1	0	1	C/C	C/C	A/A
control-1143	1	57	1	0	1	C/T	C/C	A/A
control-1144	1	58	1	1	0	T/T	G/C	A/A
control-1145	1	56	1	1	0	C/T	G/C	A/A
control-1146	1	60	0	1	1	T/T	C/C	A/A
control-1147	2	58	0	0	0	T/T	G/C	A/A
control-1148	1	59	1	0	0	C/T	G/G	G/A
control-1149	1	64	0	1	1	C/T	C/C	A/A
control-1150	2	59	0	0	1	C/C	G/G	A/A
control-1151	1	75	1	0	1	T/T	G/G	G/A
control-1152	1	68	0	0	1	T/T	G/C	A/A
control-1153	1	71	0	0	1	T/T	C/C	A/A
control-1154	1	70	0	0	0	T/T	G/C	A/A
control-1155	1	67	1	1	1	C/T	C/C	G/A
control-1156	1	64	1	0	0	C/T	C/C	G/A
control-1157	1	63	0	1	0	C/C	G/C	G/A
control-1158	1	61	1	1	0	C/C	C/C	A/A
control-1159	2	61	0	0	1	C/C	G/G	G/A
control-1160	1	52	1	0	1	T/T	G/C	A/A
control-1161	1	59	1	0	1	C/T	G/C	A/A
control-1162	1	68	1	1	1	C/T	G/C	G/A
control-1163	1	68	1	1	0	C/T	C/C	G/A
control-1164	1	70	1	1	1	C/T	C/C	A/A
control-1165	1	78	1	0	1	C/T	G/C	A/A
control-1166	1	67	1	0	1	C/C	G/G	A/A
control-1167	1	65	1	1	0	C/T	G/C	G/A

control-1168	1	63	1	0	0	T/T	C/C	G/A
control-1169	1	64	1	1	0	C/C	G/C	A/A
control-1170	1	63	1	0	1	C/T	G/C	G/A
control-1171	1	61	0	0	1	C/T	C/C	G/A
control-1172	2	63	0	0	0	T/T	C/C	G/A
control-1173	2	59	0	0	1	T/T	G/G	A/A
control-1174	1	65	0	0	1	C/T	C/C	A/A
control-1175	2	62	0	0	0	C/C	G/C	G/A
control-1176	1	66	0	0	0	T/T	G/C	A/A
control-1177	1	74	1	0	1	C/T	C/C	A/A
control-1178	1	78	1	1	0	C/T	G/C	A/A
control-1179	2	66	0	0	1	C/C	G/C	A/A
control-1180	1	70	1	0	1	T/T	G/C	A/A
control-1181	1	61	1	1	1	C/T	C/C	G/A
control-1182	2	56	0	0	1	T/T	G/C	A/A
control-1183	1	55	1	1	1	C/T	G/C	A/A
control-1184	2	64	0	0	1	C/C	C/C	A/A
control-1185	1	50	0	1	1	C/C	C/C	G/A
control-1186	2	66	0	0	1	C/T	G/C	A/A
control-1187	2	61	0	0	0	C/T	G/C	A/A
control-1188	1	51	1	0	1	C/T	G/C	A/A
control-1189	1	51	0	0	1	C/T	G/G	A/A
control-1190	1	54	0	0	1	C/T	G/G	G/A
control-1191	1	51	0	1	1	C/C	G/C	G/A
control-1192	1	52	1	0	1	C/T	C/C	A/A
control-1193	1	50	0	0	0	C/T	G/G	A/A
control-1194	1	54	0	0	0	C/T	G/G	G/A
control-1195	1	53	1	1	0	C/T	G/C	G/A
control-1196	1	59	1	1	0	C/C	G/C	G/A
control-1197	1	65	0	0	1	C/T	C/C	A/A
control-1198	1	56	1	1	0	C/T	G/G	A/A
control-1199	1	52	1	1	0	C/C	G/C	A/A
control-1200	2	53	0	0	1	C/T	G/C	A/A
control-1201	1	56	1	1	1	C/T	C/C	A/A
control-1202	2	65	0	0	0	T/T	G/C	A/A
control-1203	1	62	1	1	1	T/T	C/C	G/A
control-1204	2	59	0	0	1	C/T	G/C	A/A

control-1205	1	55	1	0	1	T/T	G/C	G/G
control-1206	1	69	0	0	1	C/T	G/C	A/A
control-1207	1	55	0	0	1	C/T	G/G	A/A
control-1208	2	50	0	0	1	T/T	G/C	G/A
control-1209	2	56	0	0	1	C/T	G/G	A/A
control-1210	1	53	1	1	0	C/T	C/C	A/A
control-1211	2	55	0	0	0	T/T	G/C	A/A
control-1212	1	80	1	0	1	C/C	C/C	G/A
control-1213	1	62	0	0	1	C/T	G/C	A/A
control-1214	2	64	0	0	1	T/T	C/C	A/A
control-1215	1	49	0	0	1	T/T	G/G	A/A
control-1216	1	63	0	0	0	T/T	C/C	A/A
control-1217	2	61	0	0	1	T/T	G/C	A/A
control-1218	1	51	0	0	1	T/T	G/C	A/A
control-1219	1	47	0	0	0	C/T	G/G	A/A
control-1220	1	49	0	0	0	T/T	G/C	G/A
control-1221	2	62	0	0	1	C/T	G/G	A/A
control-1222	2	68	0	0	1	C/T	C/C	A/A
control-1223	1	40	0	0	0	C/T	C/C	A/A
control-1224	1	73	1	0	0	T/T	G/C	G/A
control-1225	1	59	0	0	0	T/T	G/C	G/A
control-1226	1	49	0	0	0	C/C	C/C	G/A
control-1227	1	63	0	0	0	C/C	C/C	G/A
control-1228	1	49	0	0	1	C/T	C/C	G/A
control-1229	2	67	0	0	1	C/T	C/C	G/A
control-1230	1	66	0	0	0	C/T	G/C	G/A
control-1231	1	42	1	1	0	T/T	G/G	A/A
control-1232	1	41	0	0	0	T/T	G/G	A/A
control-1233	2	56	0	0	1	T/T	G/G	G/A
control-1234	1	60	1	1	0	C/T	G/C	G/A
control-1235	1	65	1	0	0	C/C	C/C	A/A
control-1236	2	64	0	0	1	C/C	C/C	A/A
control-1237	1	55	0	0	0	T/T	C/C	A/A
control-1238	1	63	1	1	0	T/T	C/C	A/A
control-1239	1	75	0	0	1	C/C	G/G	G/A
control-1240	1	50	0	1	0	C/C	C/C	A/A
control-1241	1	50	1	0	0	C/T	G/C	A/A

control-1242	1	40	0	0	1	C/T	C/C	A/A
control-1243	1	43	0	0	0	C/C	C/C	G/G
control-1244	1	45	0	0	0	C/T	G/G	G/G
control-1245	1	48	0	0	0	C/C	G/C	G/A
control-1246	1	48	0	0	0	T/T	G/G	A/A
control-1247	1	49	0	0	0	C/T	C/C	A/A
control-1248	1	49	0	0	1	T/T	G/C	A/A
control-1249	1	50	0	0	0	C/T	C/C	A/A
control-1250	1	51	0	0	1	C/C	C/C	A/A
control-1251	1	51	0	0	1	C/T	G/C	G/G
control-1252	1	52	0	0	0	C/T	G/G	A/A
control-1253	2	52	0	0	0	T/T	G/G	A/A
control-1254	1	52	0	0	1	C/T	C/C	G/A
control-1255	1	52	0	0	1	C/T	C/C	A/A
control-1256	2	52	0	0	1	C/T	G/C	A/A
control-1257	1	52	0	0	0	?	?	?
control-1258	1	52	0	0	0	C/T	G/C	A/A
control-1259	1	53	0	0	0	C/C	G/G	A/A
control-1260	2	53	0	0	0	C/C	G/C	A/A
control-1261	2	53	0	0	0	C/T	C/C	A/A
control-1262	1	53	0	0	1	C/T	G/C	G/A
control-1263	1	53	0	0	1	C/C	G/C	G/G
control-1264	1	54	0	0	1	T/T	C/C	A/A
control-1265	2	54	0	0	0	C/T	G/C	G/A
control-1266	1	54	0	0	0	C/T	G/C	A/A
control-1267	1	54	0	0	1	?	?	?
control-1268	1	54	0	0	0	C/C	G/C	A/A
control-1269	1	54	0	0	0	T/T	G/C	A/A
control-1270	1	54	0	0	1	T/T	C/C	G/G
control-1271	1	55	0	0	0	C/T	G/C	A/A
control-1272	1	55	0	0	1	C/T	G/C	A/A
control-1273	1	55	0	0	0	T/T	G/C	A/A
control-1274	2	55	0	0	1	C/T	C/C	A/A
control-1275	1	56	0	0	1	C/T	G/C	G/A
control-1276	1	56	0	0	1	T/T	G/C	A/A
control-1277	2	56	0	0	0	T/T	G/C	A/A
control-1278	2	56	0	0	0	T/T	C/C	G/A

control-1279	1	56	0	0	1	T/T	C/C	A/A
control-1280	1	57	0	0	0	T/T	G/C	A/A
control-1281	1	57	0	0	1	C/T	G/C	G/A
control-1282	1	57	1	0	0	T/T	C/C	A/A
control-1283	2	57	0	0	0	C/T	C/C	A/A
control-1284	1	57	0	0	1	C/T	G/C	A/A
control-1285	1	57	0	0	0	C/T	G/C	A/A
control-1286	1	58	0	0	0	T/T	G/C	A/A
control-1287	1	58	1	0	1	T/T	G/G	A/A
control-1288	2	58	0	0	0	T/T	G/C	G/A
control-1289	2	58	0	0	0	T/T	C/C	A/A
control-1290	1	58	0	0	1	C/C	G/G	G/G
control-1291	1	59	0	0	0	T/T	G/C	A/A
control-1292	1	59	0	0	1	T/T	C/C	A/A
control-1293	2	59	0	0	0	C/T	G/C	A/A
control-1294	1	59	1	0	1	T/T	G/C	A/A
control-1295	2	59	0	0	1	C/T	G/G	A/A
control-1296	2	59	0	0	0	C/C	G/C	G/A
control-1297	2	59	0	0	0	C/T	C/C	G/G
control-1298	2	59	0	0	0	T/T	G/C	A/A
control-1299	1	59	1	0	1	C/T	C/C	A/A
control-1300	2	59	0	0	1	T/T	G/C	G/A
control-1301	2	59	0	0	1	C/C	C/C	A/A
control-1302	1	59	0	0	1	C/T	G/C	A/A
control-1303	1	59	0	0	0	C/T	C/C	A/A
control-1304	1	60	0	1	0	T/T	G/C	A/A
control-1305	2	60	0	0	1	C/C	G/C	A/A
control-1306	1	60	0	0	0	C/C	G/C	G/G
control-1307	1	61	0	0	0	T/T	G/C	G/A
control-1308	1	61	1	0	0	C/C	C/C	A/A
control-1309	2	61	0	0	0	C/T	G/C	G/A
control-1310	1	61	1	1	0	T/T	C/C	A/A
control-1311	2	61	0	0	0	C/T	G/C	G/A
control-1312	1	61	0	1	0	T/T	G/G	G/A
control-1313	1	61	1	0	0	C/T	C/C	A/A
control-1314	1	61	0	0	1	C/C	G/G	A/A
control-1315	1	62	0	0	1	C/T	G/G	A/A

control-1316	2	62	0	0	0	T/T	G/G	G/A
control-1317	1	62	0	0	1	C/T	G/G	G/A
control-1318	1	62	0	0	0	T/T	G/G	A/A
control-1319	2	62	0	0	0	C/T	C/C	G/A
control-1320	2	62	0	0	0	C/T	G/C	G/G
control-1321	2	62	0	0	1	C/T	C/C	A/A
control-1322	1	62	0	0	0	C/T	G/C	A/A
control-1323	1	63	0	0	0	C/C	G/C	A/A
control-1324	1	63	0	0	0	T/T	G/G	G/A
control-1325	1	63	0	0	1	C/C	G/C	A/A
control-1326	1	63	1	0	0	C/T	G/C	A/A
control-1327	2	63	0	0	1	C/T	G/C	G/A
control-1328	1	63	1	0	1	C/C	C/C	A/A
control-1329	2	63	0	0	1	C/T	G/C	A/A
control-1330	1	63	1	1	1	C/T	C/C	A/A
control-1331	2	63	0	0	1	C/T	G/C	A/A
control-1332	2	63	0	0	0	C/T	G/C	A/A
control-1333	2	63	0	0	1	C/T	G/C	A/A
control-1334	1	63	1	1	0	T/T	G/C	A/A
control-1335	1	64	0	0	0	C/T	G/C	G/A
control-1336	2	64	0	0	1	C/T	C/C	A/A
control-1337	1	64	1	1	0	T/T	G/C	G/A
control-1338	2	64	0	0	0	C/C	C/C	A/A
control-1339	1	64	0	1	1	C/C	C/C	A/A
control-1340	1	64	0	0	1	C/T	C/C	G/A
control-1341	1	64	0	0	1	T/T	C/C	A/A
control-1342	2	64	0	0	0	C/C	C/C	A/A
control-1343	1	65	0	0	1	C/T	G/C	G/A
control-1344	1	65	0	0	0	C/C	C/C	A/A
control-1345	2	65	0	0	0	C/T	G/C	A/A
control-1346	1	65	1	0	1	C/C	G/C	A/A
control-1347	1	65	1	0	0	C/T	C/C	A/A
control-1348	2	65	0	0	1	C/T	C/C	G/A
control-1349	1	65	0	0	1	T/T	G/C	A/A
control-1350	1	65	0	0	0	T/T	G/C	A/A
control-1351	1	65	1	0	0	C/T	G/C	G/A
control-1352	2	**66**	0	**0**	0	C/C	C/C	A/A

control-1353	1	66	0	0	0	C/T	G/G	A/A
control-1354	2	66	0	0	0	C/T	G/G	A/A
control-1355	1	67	0	0	0	C/T	C/C	G/A
control-1356	1	67	0	0	0	C/T	G/C	A/A
control-1357	2	67	0	0	0	C/T	G/C	A/A
control-1358	1	67	0	0	1	C/C	C/C	G/A
control-1359	2	67	0	0	0	C/T	G/C	A/A
control-1360	1	67	1	0	1	T/T	G/C	A/A
control-1361	1	67	0	0	1	C/C	G/C	A/A
control-1362	1	68	0	0	1	C/T	C/C	A/A
control-1363	2	68	0	0	1	C/T	C/C	A/A
control-1364	2	68	0	0	0	C/T	G/G	A/A
control-1365	2	68	0	0	0	C/C	G/C	G/A
control-1366	2	68	0	0	1	T/T	G/C	A/A
control-1367	1	68	1	0	1	C/T	C/C	A/A
control-1368	1	68	0	0	0	C/C	G/C	A/A
control-1369	1	69	1	0	0	T/T	G/C	A/A
control-1370	1	69	0	0	0	C/T	G/G	A/A
control-1371	1	69	1	0	0	C/T	C/C	A/A
control-1372	1	69	0	0	1	T/T	C/C	A/A
control-1373	1	69	0	0	1	T/T	G/C	A/A
control-1374	1	69	0	0	1	T/T	G/C	A/A
control-1375	1	69	1	1	0	C/T	G/C	A/A
control-1376	1	70	1	1	0	T/T	G/G	A/A
control-1377	2	70	0	0	1	T/T	C/C	A/A
control-1378	1	70	0	0	0	C/T	C/C	A/A
control-1379	1	70	1	1	0	T/T	C/C	A/A
control-1380	2	71	0	0	0	C/C	C/C	A/A
control-1381	1	71	1	1	0	C/T	G/C	A/A
control-1382	1	71	0	0	1	C/C	C/C	A/A
control-1383	2	71	0	0	1	C/T	G/G	G/A
control-1384	1	71	0	1	1	C/T	C/C	A/A
control-1385	2	71	0	0	1	C/T	G/G	A/A
control-1386	1	71	0	0	0	T/T	G/C	A/A
control-1387	1	71	1	0	0	C/T	C/C	G/A
control-1388	1	71	1	0	0	T/T	C/C	G/G
control-1389	1	72	0	0	0	C/T	G/C	A/A

control-1390	1	72	0	0	0	C/C	G/C	A/A
control-1391	1	72	0	0	0	T/T	G/C	A/A
control-1392	1	72	0	0	1	C/T	G/C	G/A
control-1393	2	73	0	0	1	C/T	C/C	G/A
control-1394	1	73	1	1	0	C/T	C/C	A/A
control-1395	1	73	0	0	1	C/C	G/C	A/A
control-1396	2	73	0	0	0	C/C	G/G	G/A
control-1397	2	73	0	0	0	C/T	G/C	G/A
control-1398	2	73	0	0	1	C/C	G/G	A/A
control-1399	2	73	0	0	0	C/C	G/C	A/A
control-1400	1	73	0	0	1	T/T	C/C	G/A
control-1401	1	74	1	0	1	T/T	G/G	A/A
control-1402	1	74	1	1	0	C/C	G/C	A/A
control-1403	1	74	1	1	0	C/T	C/C	G/A
control-1404	1	74	0	0	0	C/C	C/C	G/A
control-1405	1	74	0	0	0	C/T	G/G	A/A
control-1406	1	75	0	0	0	C/T	G/C	G/A
control-1407	1	75	1	1	0	C/T	G/C	A/A
control-1408	2	75	0	0	1	C/T	G/C	A/A
control-1409	1	75	0	0	0	T/T	C/C	A/A
control-1410	1	75	0	0	0	C/T	G/C	G/A
control-1411	1	75	0	0	1	T/T	G/C	A/A
control-1412	1	76	0	0	0	C/C	G/G	A/A
control-1413	1	76	0	0	0	C/T	C/C	A/A
control-1414	2	77	0	0	0	C/T	C/C	A/A
control-1415	2	77	0	0	1	C/C	C/C	A/A
control-1416	1	77	0	0	1	C/T	C/C	A/A
control-1417	1	77	0	0	0	C/T	G/C	A/A
control-1418	1	78	1	0	0	T/T	G/C	A/A
control-1419	1	78	0	0	0	C/T	C/C	A/A
control-1420	1	79	0	0	0	C/T	C/C	A/A
control-1421	1	79	0	0	0	C/T	G/C	G/A
control-1422	1	79	0	0	0	C/T	G/C	A/A
control-1423	2	80	0	0	1	C/T	C/C	G/A
control-1424	1	69	0	0	0	C/T	C/C	A/A
control-1425	2	79	0	0	1	C/T	G/C	G/A
control-1426	1	63	0	0	1	C/T	G/G	A/A

control-1427	1	61	0	0	0	C/C	C/C	G/A
control-1428	1	55	0	0	1	C/T	G/C	A/A
control-1429	1	77	0	0	0	C/C	C/C	A/A
control-1430	1	63	0	0	0	T/T	G/G	G/G
control-1431	2	61	0	0	1	C/T	G/C	A/A
control-1432	1	92	0	0	0	C/C	G/G	A/A
control-1433	2	69	0	0	0	C/T	C/C	A/A
control-1434	2	47	0	0	0	C/T	C/C	A/A
control-1435	1	46	0	0	0	T/T	C/C	G/A
control-1436	1	65	1	1	0	C/T	G/C	G/A
control-1437	1	50	0	0	1	C/C	G/C	G/A
control-1438	2	76	0	0	1	T/T	G/C	A/A
control-1439	2	53	0	0	1	C/T	G/C	A/A
control-1440	1	65	0	0	1	C/T	G/C	A/A
control-1441	1	38	0	0	1	C/C	C/C	A/A
control-1442	2	46	0	0	0	C/T	G/G	A/A
control-1443	2	69	0	0	0	C/T	C/C	G/A
control-1444	1	74	0	0	0	C/T	G/G	A/A
control-1445	1	58	1	0	1	C/T	C/C	A/A
control-1446	2	45	0	0	0	C/T	G/C	A/A
control-1447	1	61	1	0	1	T/T	G/C	A/A
control-1448	1	68	0	0	0	C/T	G/C	A/A
control-1449	2	47	0	0	1	C/C	G/C	G/A
control-1450	1	87	0	0	0	C/C	C/C	A/A
control-1451	1	57	0	1	0	C/T	G/C	A/A
control-1452	2	46	0	0	1	T/T	C/C	A/A
control-1453	2	58	0	0	0	C/T	C/C	A/A
control-1454	1	44	1	1	1	T/T	G/G	G/A
control-1455	1	58	0	1	1	?	?	?
control-1456	1	61	1	0	0	C/T	C/C	G/A
control-1457	1	57	0	0	0	T/T	G/C	G/A
control-1458	2	63	0	0	1	C/T	G/C	G/A
control-1459	2	49	1	1	0	T/T	G/C	A/A
control-1460	2	64	0	0	0	C/T	C/C	A/A
control-1461	1	59	0	0	0	C/T	G/G	A/A
control-1462	2	44	0	0	0	C/T	G/C	G/A
control-1463	2	62	0	0	0	C/C	C/C	A/A

control-1464	2	73	0	0	1	C/T	G/C	G/A
control-1465	1	60	1	0	0	T/T	C/C	A/A
control-1466	2	62	0	0	0	C/T	G/C	G/A
control-1467	1	51	1	0	1	C/T	C/C	A/A
control-1468	1	73	0	0	1	C/T	C/C	G/A
control-1469	1	75	0	0	0	C/C	C/C	A/A
control-1470	2	47	0	0	1	C/C	G/C	G/A
control-1471	1	44	0	0	0	C/C	C/C	A/A
control-1472	1	79	0	0	0	T/T	C/C	G/A
control-1473	2	65	0	0	1	C/T	C/C	A/A
control-1474	1	68	0	0	0	C/T	C/C	A/A
control-1475	2	43	0	0	0	C/C	G/C	A/A
control-1476	1	79	0	0	1	C/T	G/C	A/A
control-1477	2	51	0	0	0	T/T	G/C	A/A
control-1478	1	39	0	0	1	C/T	C/C	A/A
control-1479	2	68	0	0	0	T/T	G/C	A/A
control-1480	2	63	0	0	0	T/T	G/C	G/A
control-1481	2	44	0	0	1	C/T	G/C	A/A
control-1482	1	60	0	0	0	T/T	C/C	A/A
control-1483	1	86	0	0	0	C/T	G/C	A/A
control-1484	1	47	0	0	0	C/C	C/C	A/A
control-1485	2	79	0	0	0	C/T	G/G	A/A
control-1486	1	67	1	1	0	C/T	C/C	A/A
control-1487	1	46	0	0	0	C/C	G/C	A/A
control-1488	2	56	0	0	1	C/T	G/C	G/A
control-1489	2	74	0	0	0	C/C	C/C	G/G
control-1490	1	76	0	0	0	T/T	C/C	G/A
control-1491	1	44	1	0	0	C/T	G/C	G/A
control-1492	1	43	0	0	1	T/T	C/C	G/A
control-1493	1	74	1	1	1	C/C	G/C	G/A
control-1494	2	68	0	0	0	C/C	G/C	A/A
control-1495	1	82	0	0	0	T/T	C/C	G/A
control-1496	2	78	0	0	1	C/C	C/C	A/A
control-1497	1	64	1	0	0	C/C	C/C	A/A
control-1498	1	63	0	0	0	T/T	G/C	G/A
control-1499	2	74	0	0	0	C/T	G/C	A/A
control-1500	2	63	0	0	0	C/T	G/C	A/A

control-1501	2	70	0	0	1	C/C	C/C	A/A
control-1502	1	63	0	0	0	C/T	G/C	A/A
control-1503	1	65	0	0	0	C/T	G/C	A/A
control-1504	1	80	0	0	1	T/T	G/C	G/A
control-1505	1	74	1	1	0	C/T	C/C	G/A
control-1506	1	55	0	0	1	C/C	G/C	A/A
control-1507	2	71	0	0	0	C/T	G/C	A/A
control-1508	1	58	0	0	1	C/T	G/C	G/A
control-1509	1	53	1	1	0	C/T	G/C	G/G
control-1510	2	60	0	0	0	C/T	G/C	A/A
control-1511	1	78	0	0	1	C/C	C/C	A/A
control-1512	1	69	1	1	0	T/T	G/C	A/A
control-1513	1	53	0	0	1	T/T	G/G	A/A
control-1514	1	49	0	0	0	T/T	C/C	A/A
control-1515	2	78	0	0	1	C/T	G/G	A/A
control-1516	1	38	0	0	0	C/T	C/C	G/A
control-1517	1	47	1	1	0	C/T	G/C	A/A
control-1518	1	77	0	0	0	C/T	G/G	A/A
control-1519	2	62	0	0	0	C/T	C/C	A/A
control-1520	2	67	0	0	0	T/T	G/G	A/A
control-1521	1	54	1	0	1	C/C	C/C	A/A
control-1522	1	64	0	0	1	C/C	G/G	G/G

Table S2 data of lymph node metastases in ESCC cases

Subjects	sex (male:1, female:2)	age (year)	smoking (yes:1; no:2)	drinking (yes:1, no:0)	BMI (≥24:1, <24:0)	**N(0-3)**	rs11614913 genotypes	rs2910164 genotypes	rs3746444 genotypes
Positive-001	1	51	1	1	0	N3	C/T	G/C	A/A
Positive-002	1	76	1	0	0	N2	T/T	G/C	A/A
Positive-003	1	75	0	0	0	N2	T/T	G/C	G/A
Positive-004	1	64	0	0	0	N2	C/T	C/C	A/A
Positive-005	2	62	0	0	1	N1	C/C	C/C	G/A
Positive-006	2	62	0	0	0	N1	T/T	G/C	G/A
Positive-007	1	50	0	0	0	N3	C/T	C/C	A/A
Positive-008	1	61	1	1	0	N1	C/C	C/C	G/A
Positive-009	1	87	0	0	0	N2	C/T	G/C	G/G
Positive-010	2	59	0	0	0	N1	T/T	C/C	G/A
Positive-011	1	66	1	0	1	N1	C/T	G/C	A/A
Positive-012	1	52	1	1	0	N2	C/C	C/C	A/A
Positive-013	2	71	0	0	0	N2	T/T	G/G	G/A
Positive-014	1	71	1	1	0	N1	C/T	C/C	A/A
Positive-015	1	64	0	0	1	N1	C/T	C/C	A/A
Positive-016	1	70	0	0	0	N1	C/C	C/C	G/A
Positive-017	2	45	0	0	1	N2	C/T	C/C	A/A
Positive-018	1	53	0	1	0	N2	C/C	G/C	A/A
Positive-019	2	62	0	0	0	N2	T/T	G/C	A/A
Positive-020	1	58	1	1	0	N1	T/T	C/C	A/A
Positive-021	1	56	1	1	0	N1	C/C	C/C	A/A
Positive-022	1	47	1	1	0	N1	C/T	G/C	A/A
Positive-023	1	45	0	0	0	N2	C/T	C/C	A/A
Positive-024	1	60	1	1	0	N2	C/T	C/C	A/A
Positive-025	1	60	0	0	1	N2	C/T	G/C	A/A
Positive-026	1	65	1	0	1	N1	C/T	G/C	G/A
Positive-027	2	66	0	0	1	N3	C/T	G/C	A/A
Positive-028	2	67	0	0	1	N2	T/T	C/C	A/A
Positive-029	1	63	1	0	0	N2	T/T	G/C	A/A
Positive-030	1	63	1	0	0	N3	T/T	G/G	A/A
Positive-031	1	60	0	0	1	N2	C/T	G/C	A/A
Positive-032	1	68	0	0	0	N1	T/T	C/C	A/A

Positive-033	1	44	1	1	0	N3	C/C	C/C	G/A
Positive-034	1	71	1	0	0	N1	C/C	G/G	A/A
Positive-035	1	63	0	0	1	N2	C/T	G/C	G/A
Positive-036	1	56	1	0	0	N1	C/T	C/C	A/A
Positive-037	2	63	0	0	0	N2	C/T	G/C	A/A
Positive-038	1	63	1	1	0	N3	T/T	C/C	G/A
Positive-039	1	59	1	1	0	N3	C/C	G/C	G/A
Positive-040	1	51	1	1	0	N2	T/T	G/C	A/A
Positive-041	1	63	0	0	1	N2	C/T	G/C	G/A
Positive-042	1	52	1	0	0	N1	T/T	G/C	A/A
Positive-043	1	55	0	0	0	N1	C/T	C/C	G/G
Positive-044	2	62	0	0	0	N1	C/T	G/G	G/A
Positive-045	2	67	0	0	1	N3	C/C	G/C	A/A
Positive-046	1	57	1	0	0	N1	T/T	C/C	A/A
Positive-047	2	74	0	0	0	N2	T/T	G/C	A/A
Positive-048	1	57	1	1	0	N1	T/T	G/G	A/A
Positive-049	1	61	1	1	0	N3	T/T	G/C	A/A
Positive-050	1	59	1	1	0	N2	C/T	C/C	A/A
Positive-051	1	57	1	0	1	N3	T/T	G/C	G/A
Positive-052	1	57	1	1	0	N3	T/T	C/C	A/A
Positive-053	1	59	1	1	0	N3	C/T	G/C	A/A
Positive-054	2	66	0	0	0	N2	C/T	G/G	A/A
Positive-055	2	75	0	0	1	N1	C/C	C/C	A/A
Positive-056	1	56	1	0	1	N1	C/T	G/C	A/A
Positive-057	1	49	1	1	0	N1	T/T	G/C	A/A
Positive-058	1	54	0	0	0	N2	T/T	G/G	G/G
Positive-059	1	44	1	0	1	N2	T/T	G/C	A/A
Positive-060	1	64	1	0	0	N1	C/C	C/C	A/A
Positive-061	2	77	0	0	0	N2	C/C	C/C	A/A
Positive-062	1	63	1	0	0	N1	C/T	G/C	G/G
Positive-063	1	66	1	0	1	N2	C/C	G/C	G/A
Positive-064	2	58	0	0	0	N2	C/T	C/C	G/G
Positive-065	1	51	1	1	0	N3	C/T	C/C	A/A
Positive-066	1	64	1	0	1	N1	T/T	G/C	A/A

Positive-067	1	52	1	0	0	N 3	C/T	C/C	A/A
Positive-068	1	45	1	1	1	N 3	C/T	G/G	G/A
Positive-069	2	52	0	0	0	N 1	C/C	G/C	G/A
Positive-070	1	55	0	1	0	N 1	C/T	G/C	A/A
Positive-071	1	79	0	0	0	N 2	C/T	C/C	A/A
Positive-072	1	58	1	0	1	N 1	C/T	G/C	A/A
Positive-073	1	56	1	1	0	N 1	C/T	C/C	A/A
Positive-074	1	48	0	0	0	N 1	C/T	G/G	A/A
Positive-075	1	76	1	1	0	N 2	C/C	G/C	G/A
Positive-076	1	83	0	0	0	N 2	C/T	G/C	A/A
Positive-077	1	57	1	0	1	N 1	T/T	G/C	A/A
Positive-078	1	51	1	1	0	N 2	T/T	C/C	A/A
Positive-079	1	73	1	0	0	N 1	T/T	G/G	A/A
Positive-080	1	55	1	0	1	N 1	T/T	G/C	A/A
Positive-081	2	61	0	0	1	N 1	C/T	G/G	A/A
Positive-082	1	57	1	1	0	N 2	T/T	C/C	A/A
Positive-083	2	65	0	0	0	N 2	C/T	C/C	A/A
Positive-084	1	67	1	1	1	N 2	C/T	C/C	A/A
Positive-085	2	70	0	0	0	N 1	T/T	G/C	A/A
Positive-086	1	71	1	0	1	N 2	T/T	G/C	A/A
Positive-087	1	55	1	1	0	N 1	C/C	G/C	G/A
Positive-088	1	56	1	1	1	N 1	C/C	C/C	A/A
Positive-089	1	66	0	0	0	N 1	T/T	G/C	A/A
Positive-090	1	53	1	1	0	N 1	T/T	G/C	A/A
Positive-091	2	62	0	0	0	N 2	T/T	C/C	A/A
Positive-092	1	65	1	1	0	N 2	C/C	G/C	A/A
Positive-093	1	75	0	0	0	N 3	C/T	G/C	A/A
Positive-094	1	60	1	0	0	N 1	C/T	C/C	A/A
Positive-095	1	79	1	1	0	N 1	C/T	G/C	A/A
Positive-096	1	50	0	0	0	N 2	C/C	G/C	G/A
Positive-097	2	76	0	0	0	N 2	C/T	G/C	G/A
Positive-098	2	63	0	0	0	N 3	C/T	G/C	G/A
Positive-099	1	80	1	0	0	N 2	C/T	C/C	G/A
Positive-100	2	59	0	0	1	N1	C/T	G/G	G/G

Positive-101	1	64	0	0	1	N 2	C/T	G/C	A/A
Positive-102	1	60	0	0	0	N 2	C/T	G/G	G/A
Positive-103	1	48	0	0	0	N 2	T/T	G/C	A/A
Positive-104	1	65	1	0	0	N 2	T/T	G/C	G/A
Positive-105	1	60	0	0	1	N 2	C/T	G/G	A/A
Positive-106	1	53	0	1	0	N 3	C/C	C/C	A/A
Positive-107	1	72	0	1	0	N 1	C/C	G/G	A/A
Positive-108	1	58	1	0	1	N 2	T/T	C/C	A/A
Positive-109	1	65	0	0	0	N 1	C/T	G/C	A/A
Positive-110	1	76	0	0	1	N 2	C/T	G/C	A/A
Positive-111	1	56	1	1	1	N 2	C/T	G/C	A/A
Positive-112	1	62	1	1	1	N 1	T/T	G/G	A/A
Positive-113	1	69	1	1	0	N 1	C/T	G/C	A/A
Positive-114	1	53	1	1	0	N 2	T/T	C/C	A/A
Positive-115	1	62	0	0	1	N 1	T/T	C/C	A/A
Positive-116	1	68	1	1	0	N 3	C/T	C/C	A/A
Positive-117	1	69	1	1	0	N 1	C/C	G/C	A/A
Positive-118	1	82	1	0	0	N 2	C/C	G/C	A/A
Positive-119	1	58	1	1	1	N 2	C/T	C/C	A/A
Positive-120	1	62	1	1	0	N 2	C/T	G/C	A/A
Positive-121	1	64	1	1	0	N 2	C/C	C/C	A/A
Positive-122	1	70	1	1	0	N 1	T/T	G/C	A/A
Positive-123	1	68	0	0	0	N 3	T/T	C/C	A/A
Positive-124	1	60	0	0	0	N 1	T/T	C/C	A/A
Positive-125	1	67	1	0	0	N 2	C/T	G/C	A/A
Positive-126	1	59	1	0	0	N 1	C/C	G/C	G/A
Positive-127	1	61	1	1	0	N 1	C/C	C/C	A/A
Positive-128	2	68	0	0	0	N 2	C/T	C/C	G/G
Positive-129	1	55	1	0	1	N 1	C/T	G/G	G/A
Positive-130	1	65	0	1	0	N 1	C/T	G/C	A/A
Positive-131	2	63	0	0	1	N 1	C/T	G/G	A/A
Positive-132	2	83	0	0	0	N 1	T/T	G/C	A/A
Positive-133	1	62	0	0	0	N 1	T/T	C/C	A/A
Positive-134	1	65	1	0	0	N1	T/T	G/G	A/A

Positive-135	2	63	0	0	1	N 2	C/T	G/G	A/A
Positive-136	2	67	0	0	0	N 1	T/T	G/C	A/A
Positive-137	2	64	0	0	1	N 1	C/T	G/C	A/A
Positive-138	1	58	1	1	0	N 1	T/T	G/C	A/A
Positive-139	1	62	0	0	0	N 2	C/T	G/G	A/A
Positive-140	2	77	0	0	1	N 1	C/T	G/C	A/A
Positive-141	1	50	1	1	0	N 2	T/T	G/C	A/A
Positive-142	1	62	0	0	0	N 1	C/T	C/C	A/A
Positive-143	1	65	1	1	0	N 1	C/T	G/C	G/A
Positive-144	1	70	1	1	0	N 1	T/T	C/C	A/A
Positive-145	1	75	0	0	0	N 2	C/T	G/C	A/A
Positive-146	1	61	0	0	0	N 2	C/C	G/G	G/A
Positive-147	1	64	1	1	0	N 1	T/T	G/C	A/A
Positive-148	1	50	1	1	0	N 1	C/T	G/C	A/A
Positive-149	2	72	0	0	0	N 1	C/C	G/C	A/A
Positive-150	1	63	1	1	0	N 1	C/T	G/C	G/A
Positive-151	1	70	1	0	1	N 2	T/T	G/C	A/A
Positive-152	2	63	0	0	1	N 2	T/T	G/C	A/A
Positive-153	1	72	1	0	1	N 1	C/T	G/C	G/A
Positive-154	1	48	0	0	0	N 2	C/T	G/C	A/A
Positive-155	1	51	0	0	0	N 3	C/T	C/C	G/G
Positive-156	1	65	1	0	0	N 1	C/C	C/C	G/A
Positive-157	2	63	0	0	0	N 2	T/T	C/C	A/A
Positive-158	1	45	1	0	0	N 2	C/T	G/C	G/A
Positive-159	1	49	0	0	1	N 2	T/T	G/C	G/G
Positive-160	1	48	1	1	0	N 1	C/T	G/C	A/A
Positive-161	1	72	1	1	0	N 1	T/T	C/C	G/A
Positive-162	1	58	1	1	0	N 3	C/T	G/C	A/A
Positive-163	2	77	0	0	0	N 2	C/T	G/C	A/A
Positive-164	2	51	0	0	1	N 1	C/T	C/C	G/G
Positive-165	1	61	1	1	0	N 2	C/T	G/C	A/A
Positive-166	1	61	1	0	0	N 1	T/T	C/C	A/A
Positive-167	1	47	1	1	0	N 2	C/T	C/C	A/A
Positive-168	1	74	1	0	0	N3	T/T	G/C	A/A

Positive-169	1	50	1	1	1	N3	C/T	C/C	A/A
Positive-170	1	63	1	0	1	N1	C/T	G/C	A/A
Positive-171	1	63	1	0	0	N2	T/T	G/C	G/A
Positive-172	1	58	0	0	0	N2	C/T	C/C	A/A
Positive-173	2	69	0	0	0	N3	C/T	G/C	G/A
Positive-174	1	60	1	1	0	N2	C/T	G/C	A/A
Positive-175	1	51	1	1	0	N1	C/T	G/G	A/A
Positive-176	1	57	1	0	0	N2	C/C	G/C	G/A
Positive-177	1	56	1	1	0	N3	C/T	C/C	A/A
Positive-178	2	58	0	0	0	N2	C/T	G/G	A/A
Positive-179	2	82	0	0	0	N3	C/C	C/C	G/A
Positive-180	1	60	1	1	0	N2	T/T	G/G	G/G
Positive-181	2	68	0	0	0	N1	C/T	C/C	A/A
Positive-182	1	58	1	0	0	N2	C/T	G/G	G/A
Positive-183	1	62	1	1	0	N2	C/T	G/G	A/A
Positive-184	1	47	1	0	0	N2	C/T	G/C	A/A
Positive-185	1	57	1	0	0	N1	C/T	C/C	A/A
Positive-186	1	60	1	0	0	N1	C/T	C/C	G/A
Positive-187	1	69	1	0	0	N3	T/T	C/C	A/A
Positive-188	1	57	1	1	0	N1	C/T	G/G	A/A
Positive-189	1	67	0	1	0	N2	C/T	G/C	G/A
Positive-190	1	61	1	1	0	N2	C/T	C/C	A/A
Positive-191	1	81	1	1	0	N2	C/T	G/C	A/A
Positive-192	1	63	1	1	0	N3	T/T	G/C	A/A
Positive-193	1	64	1	0	0	N2	C/T	G/G	G/A
Positive-194	1	65	1	1	0	N1	C/T	G/C	A/A
Positive-195	1	70	0	0	1	N1	T/T	C/C	A/A
Positive-196	1	69	1	0	0	N2	T/T	G/C	A/A
Positive-197	1	67	1	1	0	N2	C/C	C/C	A/A
Positive-198	1	64	1	1	1	N1	C/C	G/C	A/A
Positive-199	2	70	0	0	1	N2	C/C	G/C	G/A
Positive-200	1	54	1	0	1	N1	C/T	G/C	A/A
Positive-201	1	47	1	1	1	N2	C/T	G/C	A/A
Positive-202	1	54	0	0	0	N2	T/T	G/G	A/A

Positive-203	1	62	0	0	0	N 1	T/T	G/C	A/A
Positive-204	1	66	1	1	0	N 1	C/T	C/C	A/A
Positive-205	1	62	1	0	0	N 1	C/C	G/C	G/A
Positive-206	1	67	0	0	0	N 1	T/T	G/C	A/A
Positive-207	1	51	1	1	1	N 2	C/T	G/C	A/A
Positive-208	1	67	1	1	0	N 2	C/T	C/C	A/A
Positive-209	1	61	1	1	0	N 2	C/C	G/C	A/A
Positive-210	1	61	0	0	0	N 2	T/T	G/G	A/A
Positive-211	2	75	0	0	0	N 2	C/T	C/C	A/A
Positive-212	2	73	0	0	0	N 2	C/T	C/C	G/A
Positive-213	1	73	0	0	0	N 2	T/T	G/C	A/A
Positive-214	1	62	1	1	1	N 1	C/T	C/C	A/A
Positive-215	1	69	0	0	0	N 1	T/T	C/C	A/A
Positive-216	2	58	0	0	0	N 2	C/T	C/C	A/A
Positive-217	1	65	1	1	0	N 1	C/T	G/C	A/A
Positive-218	1	61	1	1	1	N 2	C/T	G/C	A/A
Positive-219	2	71	0	0	0	N 1	C/C	C/C	A/A
Positive-220	1	68	1	1	0	N 2	T/T	G/C	G/A
Positive-221	1	64	0	0	0	N 1	C/T	C/C	G/A
Positive-222	1	68	0	0	1	N 2	C/T	C/C	A/A
Positive-223	1	69	0	0	0	N 1	C/T	G/C	A/A
Positive-224	2	62	0	0	0	N 2	C/T	G/G	G/A
Positive-225	1	70	1	0	0	N 2	C/T	G/C	A/A
Positive-226	1	59	1	1	0	N 1	T/T	G/C	G/A
Positive-227	1	65	1	0	1	N 3	C/C	G/C	A/A
Positive-228	2	72	0	0	1	N 1	C/C	G/C	A/A
Positive-229	1	56	1	1	0	N 1	C/T	G/G	G/A
Positive-230	2	71	0	0	0	N 1	T/T	G/G	A/A
Positive-231	2	62	0	0	1	N 2	T/T	G/C	A/A
Positive-232	1	72	0	0	0	N 1	C/C	G/C	A/A
Positive-233	1	62	0	0	1	N 2	C/T	G/G	A/A
Positive-234	1	60	1	1	0	N 1	C/T	G/C	A/A
Positive-235	1	61	0	0	0	N 2	C/C	C/C	G/A
Positive-236	1	65	0	0	1	N1	T/T	G/G	A/A

Positive-237	1	66	0	1	1	N2	C/C	G/C	G/A
Positive-238	1	75	0	0	0	N1	T/T	G/C	A/A
Positive-239	1	71	1	0	1	N1	C/T	G/C	G/A
Positive-240	1	70	0	0	1	N2	C/T	C/C	A/A
Positive-241	1	52	0	0	0	N3	C/T	G/C	G/A
Positive-242	1	53	1	1	0	N3	T/T	G/C	A/A
Positive-243	1	71	1	1	0	N1	C/T	G/C	A/A
Positive-244	2	69	1	0	0	N1	T/T	G/C	G/A
Positive-245	1	68	1	0	1	N1	C/T	G/G	A/A
Positive-246	2	67	0	0	0	N1	T/T	G/C	A/A
Positive-247	1	59	1	1	0	N2	T/T	C/C	A/A
Positive-248	2	68	0	0	0	N1	C/C	G/C	A/A
Positive-249	1	53	0	0	0	N2	C/T	G/C	A/A
Positive-250	2	58	0	0	0	N2	T/T	G/C	A/A
Positive-251	1	51	0	0	0	N2	C/T	G/C	A/A
Positive-252	2	83	0	0	1	N2	C/T	G/C	G/A
Positive-253	1	80	0	0	0	N1	C/T	G/G	A/A
Positive-254	2	66	0	0	1	N1	C/C	C/C	G/A
Positive-255	1	68	1	1	0	N1	T/T	G/C	A/A
Positive-256	2	76	0	0	0	N2	C/T	C/C	G/A
Positive-257	1	75	0	0	0	N2	C/C	G/C	A/A
Positive-258	1	60	0	0	0	N1	T/T	G/G	A/A
Positive-259	1	69	0	0	0	N1	T/T	G/G	A/A
Positive-260	1	56	1	0	1	N2	C/C	G/C	A/A
Positive-261	1	70	0	0	0	N1	C/T	C/C	G/A
Positive-262	2	77	0	0	0	N1	C/C	G/C	A/A
Positive-263	1	68	1	1	0	N1	C/T	C/C	A/A
Positive-264	1	65	1	1	0	N2	C/T	C/C	A/A
Positive-265	1	47	1	1	0	N1	T/T	G/C	A/A
Positive-266	2	69	0	0	1	N2	C/T	G/C	A/A
Positive-267	1	57	1	1	1	N2	C/T	G/C	G/A
Positive-268	1	68	1	0	0	N1	C/C	C/C	G/A
Positive-269	1	66	1	1	0	N2	C/C	C/C	A/A
Positive-270	2	73	0	0	1	N2	T/T	G/G	A/A

Positive-271	1	58	1	1	0	N1	T/T	C/C	A/A
Positive-272	1	54	1	1	0	N1	T/T	G/C	A/A
Positive-273	1	51	0	1	0	N2	C/T	G/C	G/A
Positive-274	1	52	1	0	0	N1	C/T	C/C	G/G
Positive-275	1	56	0	0	1	N1	C/C	C/C	A/A
Positive-276	1	52	1	1	0	N2	C/T	G/C	A/A
Positive-277	1	70	0	0	0	N3	T/T	C/C	A/A
Positive-278	1	52	1	1	1	N2	T/T	G/C	A/A
Positive-279	1	74	1	1	0	N1	T/T	C/C	A/A
Positive-280	1	76	1	1	1	N2	?	?	?
Positive-281	1	65	1	1	0	N1	C/C	G/C	A/A
Positive-282	1	57	1	0	1	N2	C/T	C/C	A/A
Positive-283	1	72	1	0	0	N2	T/T	G/C	G/A
Positive-284	1	49	1	1	0	N2	T/T	C/C	G/A
Positive-285	1	62	1	0	0	N1	C/T	C/C	G/G
Positive-286	2	61	0	0	0	N1	C/C	G/C	A/A
Positive-287	1	53	1	1	0	N1	C/T	G/G	A/A
Positive-288	1	54	1	1	0	N2	?	?	?
Positive-289	2	69	0	0	0	N1	T/T	G/C	A/A
Positive-290	1	54	1	0	0	N2	?	?	?
Positive-291	1	68	0	1	0	N2	T/T	G/C	A/A
Positive-292	1	66	1	0	1	N2	C/T	G/C	A/A
Positive-293	1	53	1	1	0	N1	C/C	C/C	A/A
Positive-294	1	64	0	1	0	N2	C/T	G/G	G/A
Positive-295	1	54	1	0	1	N3	C/T	C/C	A/A
Positive-296	1	54	1	0	0	N1	C/C	G/G	A/A
Positive-297	1	65	1	0	1	N2	C/C	C/C	A/A
Positive-298	2	64	0	0	1	N2	C/T	G/C	A/A
Positive-299	1	54	1	0	0	N1	C/T	G/C	G/A
Positive-300	1	50	1	0	0	N1	T/T	G/C	A/A
Positive-301	1	57	1	1	0	N2	C/T	C/C	A/A
Positive-302	2	50	0	0	0	N1	C/T	G/C	A/A
Positive-303	1	64	0	0	0	N2	T/T	C/C	A/A
Positive-304	1	62	1	1	0	N2	C/T	C/C	G/A

Positive-305	1	70	1	0	0	N1	T/T	C/C	A/A
Positive-306	1	68	1	1	1	N2	C/T	G/C	A/A
Positive-307	1	63	1	0	0	N1	?	?	?
Positive-308	1	54	0	1	0	N3	C/T	C/C	A/A
Positive-309	1	69	1	1	0	N2	C/T	G/C	A/A
Positive-310	1	63	1	1	0	N2	T/T	G/C	G/A
Positive-311	1	52	0	1	0	N2	C/T	G/C	G/A
Positive-312	1	83	1	0	0	N1	C/T	G/C	A/A
Positive-313	2	56	0	0	0	N1	T/T	C/C	A/A
Positive-314	1	64	1	1	0	N1	C/T	G/C	G/G
Positive-315	1	62	1	1	0	N2	C/T	G/C	A/A
Positive-316	2	62	1	0	0	N1	C/C	C/C	A/A
Positive-317	2	63	1	0	0	N1	C/T	G/C	A/A
Positive-318	1	63	0	0	0	N1	C/T	G/C	G/A
Positive-319	1	59	0	0	0	N1	T/T	G/C	A/A
Positive-320	1	51	1	0	0	N1	T/T	C/C	A/A
Positive-321	2	64	0	0	1	N1	T/T	C/C	G/A
Positive-322	1	67	1	0	0	N1	T/T	G/C	A/A
Positive-323	1	53	0	0	0	N3	C/T	G/G	G/G
Positive-324	1	57	1	1	0	N3	C/T	C/C	A/A
Positive-325	1	67	1	1	0	N3	C/T	C/C	G/A
Positive-326	1	48	1	0	0	N1	C/T	G/C	A/A
Positive-327	1	69	1	1	0	N2	C/C	G/C	G/A
Positive-328	1	53	0	1	0	N2	T/T	C/C	A/A
Positive-329	1	65	1	0	0	N3	T/T	C/C	A/A
Positive-330	1	54	1	0	1	N1	C/T	G/C	A/A
Positive-331	1	62	0	0	0	N1	C/T	G/C	A/A
Positive-332	1	59	1	1	0	N2	C/C	G/C	A/A
Positive-333	1	76	1	0	0	N2	C/T	G/C	A/A
Positive-334	1	50	1	0	0	N1	C/C	G/C	G/A
Positive-335	1	76	1	0	1	N1	T/T	C/C	A/A
Positive-336	1	76	0	0	0	N2	C/T	C/C	A/A
Positive-337	1	55	1	0	0	N1	C/C	G/C	A/A
Positive-338	1	69	0	1	0	N2	C/T	G/C	A/A

Positive-339	1	61	1	0	0	N1	C/T	G/G	A/A
Positive-340	1	72	0	0	0	N2	C/C	G/C	A/A
Positive-341	1	65	0	1	0	N2	C/T	C/C	A/A
Positive-342	1	48	0	0	0	N2	C/T	C/C	A/A
Positive-343	1	74	1	0	0	N2	C/T	G/C	G/A
Positive-344	1	68	0	0	0	N1	T/T	G/C	A/A
Positive-345	1	50	1	1	0	N1	C/C	C/C	A/A
Positive-346	1	69	1	1	0	N2	C/C	G/C	G/A
Positive-347	2	67	0	0	0	N1	C/C	C/C	G/A
Positive-348	1	58	1	0	0	N1	T/T	G/C	A/A
Positive-349	1	71	0	0	0	N2	C/T	G/C	G/A
Positive-350	1	75	0	0	0	N1	T/T	C/C	A/A
Positive-351	1	58	1	1	1	N2	C/T	C/C	A/A
Positive-352	1	68	1	0	0	N1	C/C	G/C	A/A
Positive-353	1	57	1	0	0	N1	C/T	G/G	A/A
Positive-354	1	59	0	0	1	N1	T/T	G/C	A/A
Positive-355	1	62	1	1	0	N1	T/T	C/C	A/A
Positive-356	1	47	1	0	1	N2	T/T	G/C	A/A
Positive-357	1	42	1	1	0	N1	C/T	G/C	A/A
Positive-358	1	63	1	1	0	N2	C/T	G/C	A/A
Positive-359	1	79	1	0	0	N2	C/T	C/C	G/A
Positive-360	1	52	1	1	0	N1	T/T	G/C	A/A
Positive-361	1	62	1	0	0	N1	T/T	G/C	A/A
Positive-362	1	55	0	0	0	N2	C/T	G/C	A/A
Positive-363	1	56	1	0	0	N1	C/C	C/C	G/A
Positive-364	1	58	1	0	0	N3	T/T	C/C	A/A
Positive-365	1	71	1	0	0	N1	C/T	G/C	G/G
Positive-366	2	68	0	0	0	N2	C/T	C/C	A/A
Positive-367	1	67	1	0	0	N2	C/C	G/C	G/A
Positive-368	1	50	1	1	0	N2	C/T	C/C	A/A
Positive-369	1	60	1	0	0	N1	C/C	C/C	A/A
Positive-370	1	53	1	1	0	N1	C/T	C/C	A/A
Positive-371	1	71	1	0	0	N1	T/T	C/C	A/A
Positive-372	1	67	1	0	0	N2	C/T	G/C	A/A

Positive-373	1	51	1	0	0	N1	C/C	G/C	A/A
Positive-374	1	57	1	0	0	N3	C/T	G/C	A/A
Positive-375	1	86	1	1	0	N2	C/T	C/C	G/A
Positive-376	1	60	1	0	0	N1	C/C	G/G	A/A
Positive-377	1	50	1	0	0	N2	T/T	G/C	A/A
Positive-378	1	64	0	0	1	N2	T/T	G/C	G/A
Positive-379	1	72	1	1	0	N1	C/T	G/C	A/A
Positive-380	1	76	0	0	0	N1	C/C	G/C	A/A
Positive-381	1	63	1	1	0	N3	T/T	G/C	G/A
Positive-382	1	45	1	1	0	N1	C/T	C/C	A/A
Positive-383	1	68	0	0	1	N1	C/T	C/C	G/A
Positive-384	1	64	1	0	1	N1	T/T	G/G	G/A
Positive-385	2	63	0	0	0	N2	C/T	C/C	A/A
Positive-386	1	54	1	1	0	N1	C/T	G/G	A/A
Positive-387	1	60	0	0	1	N1	C/C	G/C	G/A
Positive-388	1	62	1	0	0	N2	C/T	G/C	A/A
Positive-389	1	60	1	1	0	N3	C/C	G/C	G/G
Positive-390	1	63	1	0	0	N2	C/T	G/C	A/A
Positive-391	1	60	1	0	0	N1	?	?	?
Positive-392	2	55	0	0	1	N1	C/T	G/C	A/A
Positive-393	2	67	0	0	0	N1	C/T	C/C	A/A
Positive-394	1	69	0	0	1	N2	C/T	G/C	G/A
Positive-395	1	55	1	0	0	N2	C/T	G/C	G/G
Positive-396	1	54	1	1	0	N1	T/T	G/C	A/A
Positive-397	2	68	0	0	0	N2	C/T	G/G	A/A
Positive-398	1	46	1	0	0	N1	C/T	G/C	A/A
Positive-399	1	62	0	0	0	N1	C/T	C/C	A/A
Positive-400	1	44	1	1	0	N2	C/T	G/C	A/A
Positive-401	1	59	1	1	0	N1	C/C	C/C	G/A
Positive-402	1	64	1	1	0	N1	?	?	?
Positive-403	1	59	1	0	0	N2	T/T	C/C	G/G
Positive-404	1	64	0	0	0	N3	C/T	C/C	G/A
Positive-405	1	76	0	0	0	N2	C/T	C/C	A/A
Positive-406	1	50	1	0	0	N1	?	?	?

Positive-407	1	69	0	0	0	N1	C/T	G/C	A/A
Positive-408	1	51	1	0	0	N2	C/C	G/G	G/A
Positive-409	1	68	1	0	1	N2	T/T	G/C	A/A
Positive-410	1	78	1	0	0	N1	T/T	C/C	A/A
Positive-411	1	54	1	1	0	N2	C/T	C/C	A/A
Positive-412	1	59	1	1	0	N3	T/T	G/C	G/G
Positive-413	1	70	1	0	0	N2	?	?	?
Positive-414	1	48	1	1	0	N2	C/T	C/C	A/A
Positive-415	1	64	0	0	0	N3	C/T	G/C	A/A
Positive-416	1	62	0	0	0	N1	C/T	G/C	A/A
Positive-417	1	41	1	0	1	N1	?	?	?
Positive-418	1	50	1	1	0	N2	?	?	?
Positive-419	2	64	1	0	1	N1	C/T	G/C	G/G
Positive-420	1	61	0	0	1	N1	?	?	?
Positive-421	1	59	1	0	0	N2	T/T	G/C	A/A
Positive-422	1	59	1	0	0	N2	T/T	G/C	A/A
Positive-423	1	49	1	0	0	N1	?	?	?
Positive-424	1	67	0	0	0	N1	C/T	C/C	A/A
Positive-425	1	49	1	0	0	N2	T/T	C/C	G/A
Positive-426	1	72	0	0	0	N1	T/T	C/C	G/A
Positive-427	2	66	0	0	0	N1	C/C	G/C	G/A
Negative-001	2	78	0	0	0	N0	C/C	G/C	A/A
Negative-002	1	63	1	1	0	N0	T/T	C/C	A/A
Negative-003	1	81	0	0	0	N0	T/T	C/C	A/A
Negative-004	1	50	1	0	0	N0	C/T	G/C	G/A
Negative-005	1	61	0	0	0	N0	C/T	C/C	G/A
Negative-006	1	59	1	1	0	N0	T/T	C/C	A/A
Negative-007	1	42	0	0	1	N0	T/T	G/C	A/A
Negative-008	2	79	0	0	0	N0	T/T	G/G	A/A
Negative-009	1	43	0	0	1	N0	C/T	G/C	A/A
Negative-010	1	59	1	1	0	N0	T/T	C/C	A/A
Negative-011	1	62	1	0	0	N0	C/T	G/C	G/A
Negative-012	1	46	1	1	1	N0	C/C	G/G	G/A
Negative-013	2	46	0	0	0	N0	C/T	G/C	A/A

Negative-014	1	73	1	1	0	N 0	C/T	C/C	A/A
Negative-015	1	64	1	0	1	N 0	C/T	C/C	G/A
Negative-016	1	70	1	0	0	N 0	C/T	G/C	G/A
Negative-017	2	71	0	0	0	N 0	C/T	G/C	A/A
Negative-018	2	52	0	0	0	N 0	T/T	C/C	A/A
Negative-019	1	63	1	1	0	N 0	C/T	C/C	G/A
Negative-020	1	60	1	0	1	N 0	C/C	C/C	A/A
Negative-021	1	41	1	1	0	N 0	C/T	G/C	A/A
Negative-022	1	60	1	1	0	N 0	T/T	G/C	A/A
Negative-023	1	62	0	0	0	N 0	C/T	G/G	G/A
Negative-024	1	61	1	1	0	N 0	C/T	C/C	G/A
Negative-025	1	64	1	1	1	N 0	C/T	G/C	A/A
Negative-026	1	67	0	0	0	N 0	C/T	C/C	A/A
Negative-027	2	59	0	0	0	N 0	T/T	G/C	A/A
Negative-028	1	64	1	0	0	N 0	C/T	G/C	A/A
Negative-029	1	58	1	0	1	N 0	C/T	G/C	G/G
Negative-030	1	61	1	1	0	N 0	T/T	C/C	A/A
Negative-031	1	60	1	1	0	N 0	C/T	G/G	A/A
Negative-032	1	63	1	0	0	N 0	C/C	C/C	A/A
Negative-033	1	64	1	1	0	N 0	T/T	G/C	A/A
Negative-034	1	74	1	0	0	N 0	T/T	G/C	A/A
Negative-035	1	60	1	0	0	N 0	C/T	C/C	A/A
Negative-036	1	59	1	0	1	N 0	C/T	G/C	A/A
Negative-037	1	57	0	0	1	N 0	T/T	G/C	G/A
Negative-038	1	58	0	0	0	N 0	T/T	C/C	A/A
Negative-039	2	64	0	0	0	N 0	C/T	G/C	A/A
Negative-040	2	69	0	0	0	N 0	C/T	C/C	A/A
Negative-041	2	62	0	0	0	N 0	C/C	G/G	A/A
Negative-042	1	66	0	0	0	N 0	T/T	G/G	G/A
Negative-043	1	61	0	0	0	N 0	C/T	G/C	A/A
Negative-044	2	61	0	0	0	N 0	C/C	G/C	A/A
Negative-045	1	66	0	0	0	N 0	C/T	G/C	A/A
Negative-046	2	63	0	0	0	N 0	C/C	G/C	A/A
Negative-047	1	67	0	0	1	N0	C/T	G/G	A/A

Negative-048	1	63	1	1	0	N 0	C/T	C/C	A/A
Negative-049	1	58	0	0	1	N 0	C/C	G/C	A/A
Negative-050	1	80	0	0	0	N 0	C/T	C/C	G/A
Negative-051	1	65	1	0	0	N 0	C/C	C/C	A/A
Negative-052	2	74	0	0	0	N 0	C/T	C/C	G/A
Negative-053	2	62	0	0	0	N 0	T/T	G/C	A/A
Negative-054	1	65	0	0	1	N 0	C/C	G/C	A/A
Negative-055	1	68	1	0	1	N0	T/T	G/C	A/A
Negative-056	2	46	0	0	0	N 0	C/T	G/C	G/A
Negative-057	2	61	0	0	1	N 0	C/T	G/C	A/A
Negative-058	1	58	1	1	0	N 0	C/T	G/C	A/A
Negative-059	1	67	0	0	0	N 0	C/T	G/G	A/A
Negative-060	2	66	0	0	0	N 0	T/T	G/C	A/A
Negative-061	2	66	0	0	0	N 0	?	?	?
Negative-062	1	69	0	0	0	N 0	?	?	?
Negative-063	2	69	0	0	0	N 0	C/T	C/C	A/A
Negative-064	1	64	0	0	1	N 0	C/C	C/C	A/A
Negative-065	1	61	0	0	1	N 0	C/C	G/C	A/A
Negative-066	2	64	0	0	0	N 0	C/T	G/C	A/A
Negative-067	1	65	1	0	0	N 0	C/T	C/C	A/A
Negative-068	1	66	1	1	0	N 0	C/C	G/C	A/A
Negative-069	2	71	0	0	0	N 0	C/T	C/C	G/A
Negative-070	1	64	0	1	0	N 0	T/T	G/C	A/A
Negative-071	1	64	1	1	0	N 0	C/C	C/C	A/A
Negative-072	1	47	1	0	1	N 0	T/T	C/C	A/A
Negative-073	1	65	0	0	1	N 0	T/T	G/C	A/A
Negative-074	1	64	0	0	1	N 0	C/C	C/C	A/A
Negative-075	2	60	0	0	1	N 0	C/T	G/C	G/A
Negative-076	2	62	0	0	0	N 0	T/T	G/C	A/A
Negative-077	1	65	1	1	0	N 0	C/C	G/C	A/A
Negative-078	2	62	0	0	0	N 0	C/T	C/C	G/A
Negative-079	1	65	1	1	0	N 0	C/T	G/G	G/A
Negative-080	1	54	1	1	1	N 0	C/T	C/C	A/A
Negative-081	1	60	0	0	0	N0	C/C	C/C	A/A

Negative-082	1	63	1	1	0	N0	C/C	C/C	G/A
Negative-083	1	65	0	0	1	N0	C/C	G/C	G/G
Negative-084	1	73	0	0	1	N0	C/T	G/G	G/A
Negative-085	1	48	1	1	1	N0	T/T	G/C	G/A
Negative-086	2	64	0	0	0	N0	C/T	C/C	A/A
Negative-087	1	71	1	1	0	N0	C/T	G/G	A/A
Negative-088	2	72	0	0	0	N0	C/C	C/C	A/A
Negative-089	1	67	1	0	1	N0	?	?	A/A
Negative-090	2	67	0	0	1	N0	C/T	C/C	A/A
Negative-091	1	63	0	0	1	N0	T/T	C/C	G/A
Negative-092	1	61	0	1	0	N0	T/T	G/C	A/A
Negative-093	1	53	1	1	0	N0	C/T	C/C	A/A
Negative-094	1	60	0	0	1	N0	C/T	G/C	A/A
Negative-095	2	62	0	0	0	N0	T/T	G/C	G/A
Negative-096	1	71	0	1	0	N0	T/T	G/G	A/A
Negative-097	1	62	1	0	0	N0	C/T	G/C	A/A
Negative-098	2	63	0	0	0	N0	C/T	G/C	A/A
Negative-099	1	60	0	1	0	N0	T/T	G/C	A/A
Negative-100	1	56	0	0	0	N0	C/T	G/G	A/A
Negative-101	1	59	1	0	0	N0	C/T	G/C	A/A
Negative-102	1	55	1	1	0	N0	C/C	G/C	G/A
Negative-103	1	72	1	0	0	N0	?	?	?
Negative-104	2	61	0	0	0	N0	C/T	C/C	A/A
Negative-105	2	84	0	0	0	N0	C/T	G/C	G/A
Negative-106	1	58	0	0	1	N0	C/T	G/C	A/A
Negative-107	1	63	1	0	0	N0	C/T	G/C	A/A
Negative-108	2	65	0	0	0	N0	C/C	G/G	A/A
Negative-109	2	79	0	0	0	N0	C/T	C/C	A/A
Negative-110	1	75	1	0	0	N0	T/T	C/C	G/A
Negative-111	2	74	0	0	1	N0	C/T	G/C	G/A
Negative-112	1	68	1	1	0	N0	C/C	G/G	A/A
Negative-113	1	64	1	1	0	N0	C/T	C/C	G/A
Negative-114	2	70	0	0	0	N0	C/C	G/C	G/A
Negative-115	2	63	0	0	0	N0	C/T	G/G	A/A

Negative-116	2	55	0	0	1	N 0	T/T	C/C	A/A
Negative-117	2	72	0	0	1	N 0	C/T	G/C	A/A
Negative-118	1	68	0	0	0	N 0	C/T	G/C	G/A
Negative-119	2	67	0	0	1	N 0	C/C	C/C	A/A
Negative-120	1	67	1	1	0	N 0	C/T	G/C	G/A
Negative-121	1	63	0	0	1	N 0	C/T	C/C	A/A
Negative-122	2	62	0	0	0	N 0	T/T	C/C	A/A
Negative-123	1	59	1	0	1	N 0	C/T	G/C	G/A
Negative-124	1	68	1	0	0	N 0	T/T	C/C	A/A
Negative-125	1	65	1	1	1	N 0	C/T	G/G	A/A
Negative-126	1	66	1	1	0	N 0	C/C	C/C	G/A
Negative-127	1	58	1	0	0	N 0	C/C	C/C	A/A
Negative-128	1	58	1	0	0	N 0	C/C	C/C	A/A
Negative-129	1	69	1	0	0	N 0	C/C	G/G	G/A
Negative-130	1	58	1	0	0	N 0	T/T	C/C	A/A
Negative-131	1	64	1	1	0	N 0	C/T	G/C	A/A
Negative-132	1	68	1	0	1	N 0	T/T	C/C	A/A
Negative-133	1	68	0	1	1	N 0	T/T	C/C	A/A
Negative-134	1	73	1	0	0	N 0	C/C	G/C	A/A
Negative-135	1	67	0	0	1	N 0	T/T	C/C	G/A
Negative-136	2	61	0	0	0	N 0	T/T	G/C	A/A
Negative-137	1	78	1	1	1	N 0	T/T	G/C	G/A
Negative-138	2	66	0	0	0	N 0	C/T	C/C	G/G
Negative-139	1	60	1	1	1	N 0	T/T	G/C	A/A
Negative-140	2	72	0	0	0	N 0	C/T	C/C	A/A
Negative-141	1	67	0	0	1	N 0	T/T	G/C	A/A
Negative-142	1	67	0	0	0	N 0	C/T	C/C	G/A
Negative-143	1	61	1	1	1	N 0	T/T	G/C	A/A
Negative-144	1	55	0	0	1	N 0	C/C	C/C	A/A
Negative-145	1	75	0	0	0	N 0	C/T	C/C	A/A
Negative-146	1	60	1	0	0	N 0	C/T	G/C	A/A
Negative-147	1	57	1	1	1	N 0	T/T	G/C	G/A
Negative-148	2	52	0	0	0	N 0	C/T	G/G	A/A
Negative-149	1	66	1	1	0	N0	C/C	C/C	A/A

Negative-150	1	67	1	0	0	N0	C/T	G/G	G/A
Negative-151	1	64	1	1	0	N0	C/T	G/C	G/A
Negative-152	1	70	0	0	1	N0	T/T	G/G	G/A
Negative-153	2	70	0	0	0	N0	C/T	G/C	A/A
Negative-154	1	72	1	0	0	N0	C/T	C/C	A/A
Negative-155	1	61	1	0	1	N0	T/T	G/C	A/A
Negative-156	2	63	0	0	0	N0	T/T	C/C	A/A
Negative-157	1	63	1	1	0	N0	C/T	C/C	G/A
Negative-158	1	47	1	1	0	N0	C/T	G/G	A/A
Negative-159	1	51	1	1	0	N0	C/T	G/C	A/A
Negative-160	2	63	0	0	1	N0	C/T	C/C	G/A
Negative-161	1	62	0	0	1	N0	C/T	G/C	A/A
Negative-162	1	61	1	1	1	N0	C/T	G/G	A/A
Negative-163	1	60	1	1	0	N0	T/T	C/C	A/A
Negative-164	1	64	0	0	0	N0	T/T	G/G	G/A
Negative-165	1	60	1	1	1	N0	C/C	G/C	A/A
Negative-166	1	55	0	0	0	N0	C/T	G/C	A/A
Negative-167	1	70	0	0	0	N0	T/T	C/C	A/A
Negative-168	1	68	1	1	0	N0	C/T	G/G	A/A
Negative-169	1	62	0	0	0	N0	C/C	G/G	A/A
Negative-170	1	58	1	1	1	N0	C/T	C/C	G/A
Negative-171	2	62	0	0	1	N0	C/C	G/C	A/A
Negative-172	2	68	0	0	0	N0	T/T	G/C	A/A
Negative-173	1	68	1	1	1	N0	C/T	G/C	G/G
Negative-174	1	64	1	0	0	N0	T/T	G/G	A/A
Negative-175	1	52	1	1	0	N0	C/T	C/C	A/A
Negative-176	1	56	1	1	0	N0	T/T	G/C	A/A
Negative-177	1	62	1	1	0	N0	C/T	G/C	A/A
Negative-178	1	51	1	1	0	N0	C/T	G/G	G/G
Negative-179	2	70	0	0	0	N0	C/T	G/G	A/A
Negative-180	1	74	1	0	0	N0	C/T	G/C	A/A
Negative-181	2	63	0	0	1	N0	T/T	C/C	A/A
Negative-182	2	65	0	0	0	N0	C/T	G/G	A/A
Negative-183	2	53	0	0	1	N0	C/C	C/C	G/G

Negative-184	1	68	1	1	0	N 0	C/C	G/C	A/A
Negative-185	1	52	1	1	0	N 0	C/T	G/C	A/A
Negative-186	1	44	1	1	1	N 0	C/T	G/C	A/A
Negative-187	1	56	1	1	0	N 0	T/T	G/G	A/A
Negative-188	2	48	0	0	1	N 0	T/T	C/C	A/A
Negative-189	1	66	1	0	0	N 0	T/T	C/C	A/A
Negative-190	2	61	0	0	0	N 0	T/T	C/C	A/A
Negative-191	2	69	0	0	0	N 0	T/T	G/G	A/A
Negative-192	1	78	1	0	0	N 0	T/T	G/C	G/A
Negative-193	1	61	1	1	1	N 0	C/T	C/C	A/A
Negative-194	1	56	1	1	0	N 0	C/T	G/G	A/A
Negative-195	1	60	1	1	0	N 0	C/C	G/C	A/A
Negative-196	1	66	0	0	0	N 0	C/T	G/G	G/G
Negative-197	1	67	0	1	1	N 0	T/T	G/C	A/A
Negative-198	1	71	0	0	0	N 0	C/T	C/C	G/A
Negative-199	2	57	0	0	1	N 0	T/T	G/C	A/A
Negative-200	1	66	1	0	0	N 0	T/T	C/C	A/A
Negative-201	2	64	0	0	0	N 0	C/T	G/G	A/A
Negative-202	1	55	1	1	0	N 0	T/T	C/C	A/A
Negative-203	1	65	1	1	1	N 0	C/T	G/C	A/A
Negative-204	1	58	1	1	0	N 0	C/T	G/C	G/A
Negative-205	1	56	1	1	0	N 0	T/T	C/C	A/A
Negative-206	2	55	0	0	1	N 0	C/T	C/C	A/A
Negative-207	1	66	0	0	0	N 0	C/T	G/G	A/A
Negative-208	2	66	0	0	0	N 0	C/C	G/C	A/A
Negative-209	1	64	0	0	0	N 0	C/T	G/G	A/A
Negative-210	1	65	1	0	1	N 0	C/T	G/G	A/A
Negative-211	2	51	0	0	0	N 0	C/T	G/C	A/A
Negative-212	1	53	1	0	0	N 0	C/T	C/C	A/A
Negative-213	1	60	1	0	0	N 0	C/T	C/C	G/A
Negative-214	2	67	0	0	0	N 0	T/T	C/C	A/A
Negative-215	2	52	0	0	0	N 0	C/C	G/G	G/A
Negative-216	1	53	1	0	0	N 0	T/T	G/G	A/A
Negative-217	2	71	0	0	0	N0	C/C	G/G	G/G

Negative-218	1	62	1	0	0	N 0	C/T	G/C	G/A
Negative-219	1	62	1	0	1	N 0	T/T	C/C	A/A
Negative-220	1	41	0	0	1	N 0	C/C	C/C	A/A
Negative-221	2	62	0	0	0	N 0	T/T	G/G	G/A
Negative-222	1	71	1	0	0	N 0	C/T	G/C	G/A
Negative-223	1	49	1	0	0	N 0	C/T	G/G	A/A
Negative-224	2	80	0	0	0	N 0	T/T	C/C	A/A
Negative-225	2	75	0	0	0	N 0	C/T	G/C	A/A
Negative-226	2	63	0	0	0	N 0	C/T	G/G	A/A
Negative-227	1	63	1	0	0	N 0	T/T	C/C	A/A
Negative-228	1	64	1	0	0	N 0	C/T	G/C	A/A
Negative-229	1	64	1	0	0	N 0	C/C	G/C	A/A
Negative-230	1	68	1	0	0	N 0	T/T	G/C	A/A
Negative-231	1	70	1	1	0	N 0	T/T	G/C	G/A
Negative-232	1	58	1	1	0	N 0	T/T	G/C	A/A
Negative-233	1	63	1	0	1	N 0	C/C	G/C	A/A
Negative-234	1	69	1	1	0	N 0	T/T	C/C	A/A
Negative-235	1	63	1	0	0	N 0	C/C	C/C	A/A
Negative-236	1	65	1	0	1	N 0	C/T	C/C	G/A
Negative-237	2	75	0	0	1	N 0	T/T	C/C	A/A
Negative-238	1	67	1	0	0	N 0	C/T	G/C	A/A
Negative-239	1	66	1	0	0	N 0	C/T	C/C	G/A
Negative-240	1	47	1	0	0	N 0	C/C	C/C	A/A
Negative-241	1	75	0	0	0	N 0	C/C	G/C	A/A
Negative-242	1	55	1	1	0	N 0	T/T	C/C	A/A
Negative-243	1	62	1	1	0	N 0	T/T	C/C	A/A
Negative-244	2	69	0	0	0	N 0	T/T	G/C	G/A
Negative-245	1	62	0	1	0	N 0	C/C	C/C	A/A
Negative-246	2	68	0	0	1	N 0	T/T	G/C	A/A
Negative-247	2	80	0	0	0	N 0	T/T	G/G	A/A
Negative-248	1	55	1	1	0	N 0	C/C	G/C	A/A
Negative-249	1	53	1	1	0	N 0	T/T	C/C	A/A
Negative-250	2	62	0	0	1	N 0	C/T	G/C	A/A
Negative-251	1	70	1	1	0	N0	T/T	G/C	A/A

Negative-252	1	61	0	0	1	N0	C/T	C/C	G/G
Negative-253	1	76	0	0	1	N0	C/C	G/C	G/A
Negative-254	1	70	0	0	0	N0	C/T	G/C	A/A
Negative-255	1	66	0	0	1	N0	C/T	G/C	A/A
Negative-256	2	67	0	0	1	N0	T/T	G/C	A/A
Negative-257	2	64	0	0	0	N0	C/T	G/C	A/A
Negative-258	1	67	1	1	0	N0	C/T	G/G	A/A
Negative-259	1	61	0	1	0	N0	C/T	G/C	A/A
Negative-260	2	69	0	0	0	N0	C/C	G/G	G/A
Negative-261	1	51	1	1	0	N0	C/C	C/C	A/A
Negative-262	1	53	1	0	1	N0	C/C	C/C	G/A
Negative-263	1	67	1	0	1	N0	C/C	G/G	G/A
Negative-264	2	65	0	0	0	N0	C/T	G/C	A/A
Negative-265	2	75	0	0	0	N0	C/T	G/C	A/A
Negative-266	2	63	0	0	1	N0	C/C	G/C	A/A
Negative-267	1	62	1	1	0	N0	C/C	G/C	A/A
Negative-268	1	59	1	1	0	N0	C/T	C/C	A/A
Negative-269	1	66	0	0	0	N0	C/T	C/C	A/A
Negative-270	2	57	0	0	0	N0	C/T	G/C	A/A
Negative-271	2	76	0	0	1	N0	C/T	G/G	A/A
Negative-272	2	66	0	0	0	N0	C/C	G/C	G/A
Negative-273	2	50	0	0	0	N0	T/T	G/C	G/A
Negative-274	2	66	0	0	0	N0	C/T	G/C	A/A
Negative-275	2	61	0	0	0	N0	T/T	G/C	A/A
Negative-276	1	47	1	0	0	N0	C/T	G/C	A/A
Negative-277	1	63	0	0	1	N0	C/C	G/C	A/A
Negative-278	2	72	0	0	0	N0	C/T	C/C	A/A
Negative-279	1	70	1	1	0	N0	C/T	G/C	A/A
Negative-280	1	62	0	0	0	N0	T/T	G/C	A/A
Negative-281	1	76	0	0	1	N0	C/C	G/C	A/A
Negative-282	1	70	1	1	0	N0	C/T	C/C	A/A
Negative-283	1	51	1	1	0	N0	C/T	G/G	G/G
Negative-284	1	64	1	1	0	N0	C/C	G/C	A/A
Negative-285	2	71	0	0	1	N0	T/T	C/C	G/A

Negative-286	1	71	0	0	0	N0	C/C	G/G	G/A
Negative-287	2	76	0	0	1	N0	T/T	G/C	G/A
Negative-288	1	66	1	0	1	N0	C/T	G/C	G/A
Negative-289	2	67	0	0	1	N0	T/T	G/C	A/A
Negative-290	1	70	0	0	0	N0	C/C	C/C	A/A
Negative-291	1	69	1	0	1	N0	C/T	G/G	A/A
Negative-292	1	45	0	0	1	N0	C/T	G/C	A/A
Negative-293	1	75	0	0	1	N0	C/T	G/C	A/A
Negative-294	1	63	1	1	0	N0	T/T	G/C	A/A
Negative-295	2	68	0	0	1	N0	C/T	G/C	A/A
Negative-296	1	60	1	0	1	N0	C/T	G/C	G/G
Negative-297	1	71	1	0	0	N0	T/T	C/C	A/A
Negative-298	1	66	1	0	1	N0	T/T	G/C	G/A
Negative-299	1	69	1	1	1	N0	C/T	G/G	A/A
Negative-300	2	66	0	0	0	N0	C/C	G/G	G/A
Negative-301	1	65	1	1	1	N0	C/T	G/C	A/A
Negative-302	1	65	0	0	0	N0	C/T	G/G	A/A
Negative-303	1	73	0	0	0	N0	C/T	G/C	A/A
Negative-304	1	74	0	0	0	N0	T/T	G/C	A/A
Negative-305	1	73	0	0	0	N0	C/C	C/C	A/A
Negative-306	2	74	0	0	0	N0	C/T	G/C	A/A
Negative-307	1	69	1	1	1	N0	C/T	G/C	A/A
Negative-308	1	51	1	1	0	N0	C/T	G/G	G/A
Negative-309	2	62	0	0	0	N0	T/T	C/C	A/A
Negative-310	2	63	0	0	1	N0	C/T	G/C	A/A
Negative-311	1	73	0	0	0	N0	T/T	G/C	A/A
Negative-312	1	69	1	1	0	N0	C/T	G/C	A/A
Negative-313	1	64	0	1	1	N0	C/T	G/C	A/A
Negative-314	1	60	1	1	0	N0	T/T	G/C	A/A
Negative-315	2	78	0	0	0	N0	C/T	C/C	A/A
Negative-316	2	66	0	0	1	N0	C/T	C/C	A/A
Negative-317	1	60	0	0	0	N0	T/T	C/C	A/A
Negative-318	1	74	0	0	0	N0	C/C	C/C	A/A
Negative-319	1	66	1	0	1	N0	T/T	C/C	A/A

Negative-320	1	65	1	0	0	N0	C/T	C/C	A/A
Negative-321	2	66	0	0	0	N0	T/T	G/C	G/A
Negative-322	1	66	1	1	1	N0	C/T	G/G	A/A
Negative-323	1	61	1	1	1	N0	C/C	C/C	A/A
Negative-324	1	76	0	0	0	N0	C/T	C/C	G/A
Negative-325	2	53	0	0	0	N0	C/T	C/C	A/A
Negative-326	2	64	0	0	1	N0	C/T	C/C	A/A
Negative-327	1	77	0	0	0	N0	C/T	G/G	A/A
Negative-328	2	63	0	0	0	N0	T/T	G/C	G/A
Negative-329	1	52	0	0	0	N0	?	?	?
Negative-330	1	57	1	1	0	N0	C/T	C/C	A/A
Negative-331	1	53	0	0	0	N0	?	?	?
Negative-332	1	50	1	1	0	N0	?	?	?
Negative-333	1	62	1	1	1	N0	C/T	C/C	A/A
Negative-334	1	65	1	0	1	N0	T/T	C/C	G/A
Negative-335	1	59	1	0	0	N0	C/T	G/C	G/A
Negative-336	2	66	0	0	0	N0	C/T	G/C	A/A
Negative-337	1	62	0	0	1	N0	C/C	G/C	A/A
Negative-338	1	70	1	0	1	N0	C/C	C/C	A/A
Negative-339	1	55	1	1	0	N0	C/T	G/C	A/A
Negative-340	1	64	1	1	0	N0	C/C	G/C	G/A
Negative-341	1	65	1	0	0	N0	C/T	G/C	G/A
Negative-342	1	62	1	0	0	N0	C/C	C/C	G/A
Negative-343	1	64	1	1	0	N0	C/C	C/C	A/A
Negative-344	2	65	0	0	0	N0	C/T	C/C	A/A
Negative-345	1	52	1	0	1	N0	C/T	C/C	G/A
Negative-346	2	53	0	0	1	N0	T/T	G/C	G/A
Negative-347	2	50	0	0	0	N0	C/T	C/C	A/A
Negative-348	1	69	1	0	1	N0	C/T	G/C	G/A
Negative-349	2	72	0	0	1	N0	C/T	G/C	A/A
Negative-350	1	65	1	1	0	N0	T/T	G/C	A/A
Negative-351	1	47	1	0	1	N0	T/T	G/C	A/A
Negative-352	1	49	1	1	0	N0	C/T	G/G	G/A
Negative-353	2	50	0	0	0	N0	C/T	G/C	G/A

Negative-354	1	57	1	0	0	N0	C/T	C/C	A/A
Negative-355	1	67	1	0	1	N0	T/T	G/C	A/A
Negative-356	1	49	1	0	0	N0	T/T	G/C	A/A
Negative-357	2	64	0	0	1	N0	C/C	C/C	A/A
Negative-358	2	63	0	0	0	N0	T/T	G/C	A/A
Negative-359	1	62	0	0	0	N0	C/T	G/C	G/A
Negative-360	1	49	1	1	0	N0	C/T	C/C	A/A
Negative-361	1	72	0	0	0	N0	T/T	G/C	A/A
Negative-362	1	51	1	0	0	N0	C/C	G/C	A/A
Negative-363	1	55	1	1	1	N0	C/T	G/C	A/A
Negative-364	2	54	0	0	0	N0	C/T	C/C	G/A
Negative-365	1	51	1	0	0	N0	T/T	G/C	A/A
Negative-366	1	67	0	0	0	N0	C/C	G/C	A/A
Negative-367	2	55	0	0	0	N0	C/T	G/C	A/A
Negative-368	1	66	1	0	1	N0	T/T	G/C	A/A
Negative-369	2	64	0	0	1	N0	T/T	C/C	G/A
Negative-370	1	76	1	0	1	N0	?	?	?
Negative-371	1	53	1	0	1	N0	C/T	C/C	A/A
Negative-372	1	63	0	0	0	N0	C/C	G/G	A/A
Negative-373	1	53	1	1	1	N0	C/C	C/C	A/A
Negative-374	1	61	1	1	1	N0	T/T	G/C	A/A
Negative-375	1	66	1	0	0	N0	T/T	C/C	A/A
Negative-376	1	62	1	0	0	N0	T/T	C/C	A/A
Negative-377	1	67	0	0	0	N0	C/T	G/C	A/A
Negative-378	2	47	0	0	0	N0	C/T	C/C	A/A
Negative-379	1	67	1	0	0	N0	?	?	?
Negative-380	1	49	1	1	0	N0	C/T	G/C	A/A
Negative-381	1	59	0	0	1	N0	T/T	C/C	A/A
Negative-382	1	67	0	0	0	N0	T/T	G/C	G/A
Negative-383	1	55	1	0	0	N0	C/T	C/C	G/A
Negative-384	1	52	0	1	1	N0	T/T	C/C	A/A
Negative-385	1	71	1	1	0	N0	?	?	?
Negative-386	2	57	0	0	0	N0	T/T	G/C	G/A
Negative-387	1	50	0	0	1	N0	T/T	G/G	A/A

Negative-388	2	61	0	0	0	N0	C/T	G/C	G/A
Negative-389	1	61	0	0	0	N0	C/T	C/C	A/A
Negative-390	1	47	0	0	0	N0	C/T	C/C	G/A
Negative-391	1	66	1	0	0	N0	C/C	G/G	G/A
Negative-392	1	69	1	1	1	N0	T/T	C/C	A/A
Negative-393	2	77	0	0	0	N0	?	?	?
Negative-394	1	58	1	1	0	N0	C/T	G/C	A/A
Negative-395	1	67	1	0	0	N0	C/C	C/C	A/A
Negative-396	1	63	1	0	0	N0	T/T	G/C	G/A
Negative-397	2	48	0	0	0	N0	C/T	G/G	A/A
Negative-398	1	79	0	0	0	N0	?	?	?
Negative-399	1	57	1	0	1	N0	C/T	C/C	G/G
Negative-400	2	64	0	0	1	N0	T/T	C/C	G/G
Negative-401	1	59	1	0	0	N0	T/T	G/C	A/A
Negative-402	1	59	1	0	0	N0	C/T	C/C	A/A

All steps of PCR:

1. For quality control and concentration evaluation, 1μl DNA samples were checked by using agarose gel electrophoresis, the concentration of most DNA samples was between 30-50ng/ul, so DNA samples were not diluted.

2. DNA lysis: 2μl DNA mixed with 2.5μl 4x DNA lysis Buffer, adding to 10μl with distilled H2O, at 98°C for 5 min after centrifuging, and then chilled the sample on ice.

3. DNA ligation reaction: 20-μl ligation mixture was first prepared (contain 10xLigase buffer 2μl, Ligase 0.5μl, ddH2O 7.5μl, Probe Mix 1μl and 10μl DNA sample) and then the ligation reaction was performed under the following cycling program: 95 °C for 5 min, 4 cycles x (94°C 1 min, 58°C for 4h), 94°C 2 min, 70°C forever.

4. Multiplex fluorescence PCR reaction: Two multiplex fluorescence PCR reactions were then performed for each ligation product. Each PCR mixture contain 2xPCR Master Mix 10μl, Primer Mix 1μl, ligation product 1μl, and ddH2O 8μl. The PCR program was described as follows: 95°C for 2 mins, 9x(94°C 20s, 62°C-0.5°C/cycle 40s, 72°C 1.5 min), 25x(94°C 20s, 57°C 40s, 72°C 1.5min), 60°C 1h, 4°C forever. We also cite the previously published article in our paper.

5. Dilute fluorescence PCR product 10 times, mixing 1μl product with 0.5μl Liz500 SIZE STANDARD, 8.5μl Hi-Di, and then denaturation at 95°C for 5min, genotyping by ABI3730XL.

6. Raw data were analyzed by GeneMapper 4.0 (AppliedBiosystems, USA) and genotypes for each locus were determined based on the information of allele specific ligation-PCR product's label- ing dye color and fragment size.

Primers were designed by software pp5 according to Tang *et al* [1], Jiang *et al* [2], Chen *et al* [3], Zhang *et al* [4], etc..

rs2910164:

rs2910164_CF (ATGGGTTGTGTCAGTGTCAGACATC)

rs2910164_GF (ATGGGTTGTGTCAGTGTCAGACATG)

rs2910164_3F (TGAAATTCAGTTCTTCAGCTGGGA)

rs11614913:

rs11614913_CF (GTTTTGAACTCGGCAACAAGAAACAGC)

rs11614913_TF (GTTTTGAACTCGGCAACAAGAAACAGT)

rs11614913_3F (CTGAGTTACATCAGTCGGTTTTCGTC)

rs3746444:

rs3746444_AR (GGAAGCAGCACAGACTTGCTGTCAT)

rs3746444_GR (GGAAGCAGCACAGACTTGCTGTTAC)

rs3746444_3R (GTTCACGTGGAGAGGAGTTAAACATC)

The SNPscanTM technology:

The SNPscanTM method was used to genotype the miRNA-499 rs3746444, miRNA-146a rs2910164, and miRNA-196a2 rs11614913 loci, which was based on double ligation and multiplex fluorescence PCR, you can get the information from the manufacturer's website

(http://www.geneskybiotech.com/en/index.php/Index/fuwuer/id/29),

or you can get the details from the previously published article written by Du *et al* [5].

[1] TANG W, WANG Y, PAN H, et al. Association of miRNA-499 rs3746444 A>G variants with adenocarcinoma of esophagogastric junction (AEG) risk and lymph node status [J]. Onco Targets Ther, 2019, 12: 6245-52.

[2] JIANG J, ZHANG S, TANG W, et al. Lack of association between miR-146a rs2910164 C/G locus and colorectal cancer: from a case-control study to a meta-analysis [J]. Biosci Rep, 2021, 41(1).

[3] CHEN Y, TANG W, LIU C, et al. miRNA-146a rs2910164 C>G polymorphism increased the risk of esophagogastric junction adenocarcinoma: a case-control study involving 2,740 participants [J]. Cancer Manag Res, 2018, 10: 1657-64.

[4] ZHANG S, CHEN L, WANG Y, et al. Investigation of the Association of miRNA-499, miRNA-146a, miRNA-196a2 Loci with Hepatocellular Carcinoma Risk: A Case-Control Study Involving 1507 Subjects [J]. DNA Cell Biol, 2020, 39(3): 379-88.

[5] DU W, CHENG J, DING H, et al. A rapid method for simultaneous multi-gene mutation screening in children with nonsyndromic hearing loss [J]. Genomics, 2014, 104(4): 264-70.
